# Quorum Sensing Coordinates Brute Force and Stealth Modes of Infection in the Plant Pathogen *Pectobacterium atrosepticum*

**DOI:** 10.1371/journal.ppat.1000093

**Published:** 2008-06-20

**Authors:** Hui Liu, Sarah J. Coulthurst, Leighton Pritchard, Peter E. Hedley, Michael Ravensdale, Sonia Humphris, Tom Burr, Gunnhild Takle, May-Bente Brurberg, Paul R. J. Birch, George P. C. Salmond, Ian K. Toth

**Affiliations:** 1 Plant Pathology Programme, SCRI, Invergowrie, Dundee, United Kingdom; 2 Department of Biochemistry, University of Cambridge, Cambridge, United Kingdom; 3 Bioforsk, Norwegian Institute for Agricultural and Environmental Research, Ås, Norway; The University of North Carolina at Chapel Hill, United States of America

## Abstract

Quorum sensing (QS) *in vitro* controls production of plant cell wall degrading enzymes (PCWDEs) and other virulence factors in the soft rotting enterobacterial plant pathogen *Pectobacterium atrosepticum* (*Pba*). Here, we demonstrate the genome-wide regulatory role of QS *in vivo* during the *Pba*–potato interaction, using a *Pba*-specific microarray. We show that 26% of the *Pba* genome exhibited differential transcription in a QS (*expI*-) mutant, compared to the wild-type, suggesting that QS may make a greater contribution to pathogenesis than previously thought. We identify novel components of the QS regulon, including the Type I and II secretion systems, which are involved in the secretion of PCWDEs; a novel Type VI secretion system (T6SS) and its predicted substrates Hcp and VgrG; more than 70 known or putative regulators, some of which have been demonstrated to control pathogenesis and, remarkably, the Type III secretion system and associated effector proteins, and coronafacoyl-amide conjugates, both of which play roles in the manipulation of plant defences. We show that the T6SS and a novel potential regulator, VirS, are required for full virulence in *Pba*, and propose a model placing QS at the apex of a regulatory hierarchy controlling the later stages of disease progression in *Pba*. Our findings indicate that QS is a master regulator of phytopathogenesis, controlling multiple other regulators that, in turn, co-ordinately regulate genes associated with manipulation of host defences in concert with the destructive arsenal of PCWDEs that manifest the soft rot disease phenotype.

## Author Summary

Many Gram-negative bacteria use a population density-dependent regulatory mechanism called quorum sensing (QS) to control the production of virulence factors during infection. In the bacterial plant pathogen *Pectobacterium atrosepticum* (formerly *Erwinia carotovora* subsp. *atroseptica*), an important model for QS, this mechanism regulates production of enzymes that physically attack the host plant cell wall. This study used a whole genome microarray-based approach to investigate the entire QS regulon during plant infection. Results demonstrate that QS regulates a much wider set of essential virulence factors than was previously appreciated. These include virulence factors similar to those in other plant and animal pathogens that have not previously been associated with QS, e.g., a Type VI secretion system (and its potential substrates), shown for the first time to be required for virulence in a plant pathogen; and the plant toxin coronafacic acid, known in other pathogens to play a role in manipulating plant defences. This study provides the first evidence that *Pectobacterium* may target host defences simultaneously with a physical attack on the plant cell wall. Moreover, the study demonstrates that a wide range of previously known and unknown virulence regulators lie within the QS regulon, revealing it to be the master regulator of virulence.

## Introduction

Quorum sensing (QS) is a population density-dependent regulatory mechanism, utilising freely diffusible chemical signal molecules, which controls a wide range of phenotypes in many different bacteria [1]. The best-studied QS systems are those utilising *N*-acyl-homoserine lactone (AHL) signal molecules, synthesised by LuxI homologues. AHL concentration increases with bacterial population growth until, at high cell density, a threshold level of signal is reached. This is detected by AHL binding to receptor proteins, LuxR-family transcriptional regulators, resulting in altered gene expression [2]. QS plays an essential role in the pathogenesis of many bacterial pathogens of both plants and animals. Amongst the best studied AHL QS systems are those of the soft rotting enterobacterial plant pathogens *Pectobacterium atrosepticum* (*Pba*) and *Pectobacterium carotovorum* subsp. *carotovorum* (*Pcc*; formerly *Erwinia carotovora* subsp. *atroseptica* and *E. c*. subsp. *carotovorum* respectively) [3]. These pathogens cause disease primarily through the coordinate and prolific production of a variety of plant cell wall degrading enzymes (PCWDEs), which are secreted to the extracellular environment through the Type I (protease) and Type II (pectinases and cellulases) secretion systems [4]. However, they also possess a Type III secretion system (T3SS) with cognate effector (DspA/E) and helper/harpin proteins (HrpN/HrpW), which is required for full virulence [5]. While the role of the T3SS in the soft rotting pathogens remains to be elucidated, in the closely-related *E. amylovora*, DspA/E has been reported to interact with leucine-rich repeat receptor-like protein kinases (LLR-RLKs) of apple plants, implying a role in the manipulation of host defences [6]. QS in pectobacteria has been reported to regulate PCWDEs [7], the Type III secreted harpin HrpN [8], and other virulence factors, including Nip and Svx [9]–[11], a very small number of virulence regulators (*expR, rsmA* and *virR*) [12]–[14], and the antibiotic carbapenem [15]. These are controlled by the AHL, *N*-(3-oxohexanoyl)-L-homoserine lactone (OHHL), synthesised by ExpI. Different strains of pectobacteria possess up to three homologues of LuxR [16] including: VirR, which plays a central role in the repression of QS-regulated virulence factors [12]; CarR, which regulates the production of carbapenem [15]; and ExpR, which activates transcription of the global repressor, *rsmA*, in the absence of AHL [13].

Until now, studies on QS in pectobacteria have largely been *in vitro* and have examined its role in the regulation of targeted virulence factors, particularly PCWDEs. Such virulence factors are thought to operate as part of a necrotrophic mode of action (where the invading organism causes death of host tissue and colonises dead substrate). As a consequence, this group of pathogens have been termed “brute force” in line with this physical attack on plant cell walls. This is in contrast to pathogens such as *Pseudomonas syringae*, which are hemibiotrophic (requiring living host tissue as part of the infection process, during which they actively manipulate host defences) and, due to their ability to manipulate plant defences as part of the infection process, have been termed “stealth” pathogens. In the pectobacteria, it has been hypothesised that QS acts to delay the onset of PCWDE production until sufficient numbers of cells are present to overcome plant defences, which are induced by the formation of cell wall breakdown products [17],[18]. However, in previous work we showed that premature addition of OHHL to potato plants infected with low numbers of *Pba* induced early disease development [19], suggesting that this hypothesis may be an over-simplification of a more complex process. In addition, the full genome sequence of *Pba* strain SCRI1043 (*Pba*1043) has revealed many additional putative virulence determinants, including coronafacoyl-amide conjugates and homologues of the hemolysin-co-regulated protein (Hcp) and Rhs accessory element VgrG [20]. In *Pseudomonas syringae*, coronafacoyl-amide conjugates promote disease development and, together with the T3SS, may act to suppress salicylic acid-based defences as part of this process [21],[22]. Hcp and VgrG have been associated with virulence in animal pathogens and are potential effector proteins delivered through a Type VI secretion system (T6SS) [23]–[26]. Hcp and VgrG homologues were recently detected in the secretome of *Pba*1043 and over-expression of *hcp1* increased *Pba* virulence, suggesting that this and other *hcp* family members are virulence determinants [27]. The presence of such determinants in *Pba* suggests that the pectobacteria may also act in a stealth-like manner by manipulating resistance during the infection process. However, whether these determinants are produced and act independently, or together with PCWDEs as part of a coordinated assault on the plant, is unknown. We developed a whole genome microarray for *Pba*1043 and report its use to study gene expression from an *expI* mutant of *Pba*1043 grown *in planta,* to determine global effects of QS on gene regulation during potato infection, with particular emphasis on the relationship between PCWDEs and possible stealth mechanisms.

## Results/Discussion

### A Mutation in *expI* Reduces Virulence and OHHL Production

**Figure 1:**
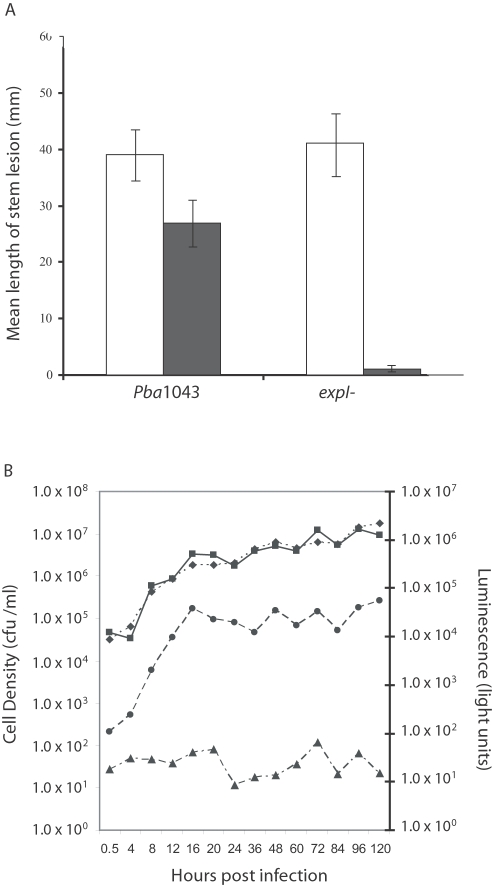
OHHL is Required for Virulence in Potato Stems and Peaks at 16 hpi in *P. atrosepticum*-infected Potato Tubers. (A) Lesion development on potato stems following inoculation of the wild type *Pba*1043 and *expI* mutant strains. Filled bars = potato cv Desiree control; open bars = OHHL-producing transgenic Desiree (YI5A) [19]. (B) Cell density and OHHL production (in light units) of wild type *P*. *atrosepticum* and *expI* mutant strains in potato tubers over 120 h. Wild type cell density (square); *expI* mutant cell density (diamond); wild type OHHL production (circle); mutant OHHL production (triangle). Bars show mean +/− standard error of the mean.

The *expI* gene and ExpI product, OHHL, are required for full virulence in *Pba* and *Pcc* [7],[12],[28]. The virulence of an *expI* (ECA0105) mutant was significantly reduced on both potato stems and tubers and was restored following complementation with the *expI* gene *in trans* (Fig. S1). To confirm that virulence could be restored *in planta* by the presence of OHHL, the *expI* mutant strain was inoculated at low cell densities into an OHHL-producing transgenic potato plant [19], where virulence was restored compared to inoculation on a non-transgenic control plant (Fig. 1A). This supports previous work where the presence of OHHL in these transgenic plants induced early disease development from low cell densities (10^2^ cells per inoculation site) of both WT and *expI* mutant strains [19].

A luminescence-based assay was used to monitor OHHL production during growth of the *expI* mutant and wild type strains in potato tubers (Fig. 1B). Both strains grew at comparable rates over a 120 h infection time course and reached similar population levels. Although the wild type and *expI* mutant strains would be expected to show differences in growth in natural condition during the course of disease development, the relatively short infection time (120 h) and the method of inoculation (onto the cut tuber surface), may account for the results observed. In the wild type, the level of OHHL rose sharply over the first 16 hours in line with log phase growth, before reaching a plateau at a concentration of approximately 80 µg/ml. At this plateau, bacterial cell density was approximately 5.0×10^6^ cfu/ml, which was similar to previous reports [15]. In the *expI* mutant, OHHL production remained at background levels. Based on the above data, time points at 12 and 20 hours post inoculation (hpi), *i.e*. just prior to and just following maximum OHHL synthesis *in planta*, were selected to study transcriptional changes during QS (Fig. 1B).

### Comparison of *Pba* Wild Type and *expI* Mutant Transcriptomes *in planta*

**Figure 2:**
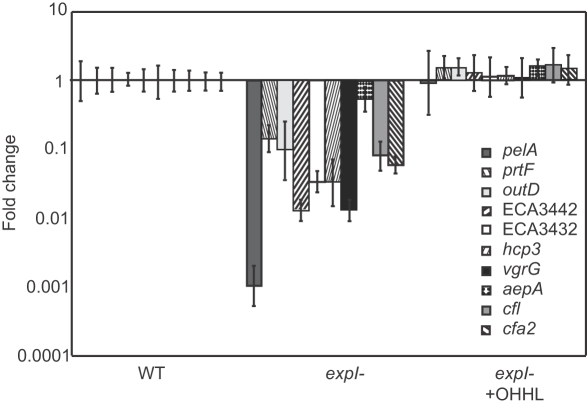
OHHL Complementation of Selected Quorum Sensing-regulated Genes. RT-PCR analysis of selected genes after 18h growth of wild type *P. atrosepticum* in Pel Minimal Medium (PMM), and growth of the *expI* mutant in PMM with and without the addition of OHHL (5 µM final concentration). *vgrG* = ECA2867. Bars show mean +/− standard deviation.

Differential expression of genes (*pelA* [ECA4067], *pelC* [ECA4069], *celV* [ECA1981], *prtW* [ECA2785], *pehA* [ECA1095], ECA2220, *svx* [ECA0931] and *nip* [ECA3087]) previously shown to be under QS control [9],[10],[12] was investigated using quantitative real-time PCR (qRT-PCR) at 12 and 20 hpi in the *expI* mutant and wild type strains. In all cases, significant up-regulation of these genes was observed in the wild type only (Table S1). cDNA from the wild type and *expI* mutant at 12 and 20 hpi was hybridised to the *Pba* microarray. 1167 coding sequences (CDSs) (approx. 26% of the genome) showed statistically significant differences (P≤0.05) in expression between the *expI* mutant and wild type (Table S2). 498 CDSs showed reduced transcript abundance (421 at 12 hpi, 169 at 20 hpi, 92 at both time points) and 687 CDSs exhibited increased transcript abundance (551 at 12 hpi, 180 at 20 hpi, 44 at both time points) in the *expI* mutant compared to the wild type. Microarray comparison of mutant and wild type cDNAs from cells in buffer solution prepared for tuber inoculation following overnight growth in LB to stationary phase (zero time-point), was consistent with there being no overall transcriptional difference (P≤0.05) between the strains prior to plant inoculation (data not shown). Only 16% of CDSs within the horizontally-acquired islands [20] showed differential gene expression, suggesting that such CDSs are less likely to have been incorporated into the QS regulon than those on the chromosome backbone. qRT-PCR was used to study a number of genes in the *expI* mutant and wild type to examine differential gene expression, either to verify changes observed in the microarray or to examine the effects of a mutation in *expI* on additional genes (Table S1). Importantly, qRT-PCR analysis of selected genes after growth of the *expI* mutant and wild type *in vitro* revealed the same pattern of *expI*-dependence as observed *in vivo*, and these changes could be fully complemented by the addition of exogenous OHHL (Fig. 2).

The role of QS in pathogenesis of pectobacteria has been intensively studied *in vitro*, particularly for its ability to co-ordinately up-regulate PCWDEs [7]–[10],[12],[14],[28]. Previous work based on enzyme plate assays observed that all major groups of PCWDEs, including pectate lyases (Pel), cellulases (Cel), protease (Prt), pectin lyase (Pnl) polygalacturonase (Peh) and pectin methyl esterase (Pme) were under QS control [8]. In this study, we found that genes encoding all these groups showed lower transcript abundance in the *expI* mutant compared to the wild type at both 12 and 20 hpi (Table 1, Table S1). The major pectate lyases PelA, PelB (ECA4068) and PelC (ECA4069), as well as CelV (ECA1981), a putative cellulase ECA2220, PrtW (ECA2785) and PehA (ECA1095) have previously been associated with QS in pectobacteria, either through transcriptional or proteomic analyses [7],[10],[12],[28]. However, in addition the transcription (using microarray and/or qRT-PCR analyses) of genes encoding other PCWDEs and their isoforms, including “minor” pectate lyases (PelZ [ECA4070], Pel-3 [ECA1094], PelB and PelW [ECA2402]), CelB (ECA2827) and CelH (ECA3646), PehN (ECA1190), PmeB (ECA0107) and Pnl (ECA1499) was found to be *expI*-dependent. These results confirm previous observations of QS regulatory control *in vitro* and validate our *in planta* approach.

**Table 1:** Microarray Analysis of Selected *P. atrosepticum* Genes *in planta.*

Gene ID	Gene name	Predicted function	F/C 12h	F/C 20h
**Plant cell wall degrading enzymes**				
ECA4067*	*pelA*	Pectate lyase I	−15.9	−12.5
ECA1094*	*pel-3*	Pectate lyase	−54.2	−43.2
ECA2553	-	Pectate lyase	−11.2	−7.4
ECA4068*	*pelB*	Pectate lyase	−28.6	−11.4
ECA4070*	*pelZ*	Pectate lyase	−4.1	−6.3
ECA2827*	*celB*	Beta(1,4)-glucan glucanohydrolase	−9.2	−3.8
ECA1499*	*pnl*	Pectin lyase	−5.5	−7.8
**Type I secretion**				
ECA2781*	*prtF*	Protease secretion	−3.1	−2.8
ECA2782*	*prtE*	Protease secretion	-	-
ECA2783*	*prtD*	Protease secretion	-	−1.9
**Type II secretion**				
ECA3100*	*outM*	Secretion prot. M	−2.7	−2.2
ECA3101*	*outL*	Secretion prot. L	−3.0	−2.3
ECA3105*	*outH*	Secretion prot. H	−3.8	−2.4
ECA3106*	*outG*	Secretion prot. G	−7.5	−3.5
ECA3107*	*outF*	Secretion prot. F	−1.8	−1.8
ECA3109*	*outD*	Secretion prot. D	−1.7	−1.5
**Type III secretion**				
ECA2097*	*hrpE*	Secretion prot.	-	−1.6
ECA2108*	-	Putative lipoprot.	−1.7	-
**Type VI secretion and putative substrates**				
ECA3444*	*-*	Hypothetical prot.	−82.0	−50.0
ECA3445*	*-*	Hypothetical prot.	−2.5	−2.0
ECA2867*	*vgrG*	VgrG homologues	−2.9	−4.3
ECA3427*	*-*	VgrG homologues	−4.8	−3.6
ECA3420	*-*	Hypothetical prot.	−7.7	−2.6
ECA3430*	*-*	Putative phospholipase	−3.8	-
ECA3432*	*vasK*	IcmF-like prot.	−5.0	−2.5
ECA3433	*-*	Hypothetical prot.	−2.6	−1.8
ECA3436*	*vasG*	ClpB-like prot.	−4.4	−3.2
ECA3440	*-*	Hypothetical prot.	−11.1	−5.6
ECA3442*	*vasA*	Hypothetical prot.	−3.7	−4.2
ECA3443	*-*	Hypothetical prot.	−5.0	−3.4
ECA4275	*hcp1*	*hcpA* homologues	−50.0	−50.0
ECA3428*	*hcp2*			
ECA2866*	*hcp3*	*hcpA* homologue	−20.0	−20.0
ECA0456*	*hcp4*	*hcpA* homologue	−14.3	−5.9
ECA3672*	*-*	*hcpA* homologue	−6.9	−4.1
ECA0176*	*-*	*hcpA* homologue	−21.3	−12.7
ECA4277	*-*	*hcpA* homologue	−20.0	−16.7
**Regulators**				
ECA0105*	^§^ *expI*	AHL synthesis	−28.2	−18.3
ECA0106*	^§^ *expR*	QS regulator	−9.4	−8.5
ECA0809	*hexY*	Global regulator	−2.4	−1.5
ECA1022*	*aepA*	Virulence regulator	−10.5	−3.7
ECA1561	^§^ *virR*	QS regulator	−1.6	−1.5
ECA1931*	*hor*	Global regulator	−4.2	−2.5
ECA2882*	*expA*	Two component regulator	−2.3	−1.8
ECA4123	*rexZ*	PCWDE regulator	−3.3	-
ECA1562*	*virS*	TetR regulator	3.7	1.8
ECA2425*	*kdgR*	Pectin degradation repressor	2.1	-
ECA2445	*pehR* (*phoP*)	Two component regulator	2.3	-
ECA3030*	*hexA*	LysR regulator	3.5	2.1
ECA3366*	^§^ *rsmA*	Global regulator	1.9	-
ECA2724*	*rscR*	LysR regulator	3.3	2.9
ECA3168*	*ohrR*	Regulator hydro-peroxide resistance	2.5	4.2
ECA1740*	*fliZ*	Alternative sigma factor regulator	−2.0	−2.9
ECA2435*	*rdgA*	Regulator of pectin lyase production	1.6	1.5
**Toxin/virulence associated proteins**				
ECA0601	*cfa8A*	Putative oxidoreductase	-	−1.8
ECA0607*	*cfa2*	Coronafacic acid dehydratase	1.7	−2.0
ECA0931*	*svx*	Putative virulence-associated prot.	−6.9	−3.3
ECA3087*	*nip*	Putative virulence-associated prot.	−34.5	−11.0
ECA3946*	*-*	Putative exported prot.	−4.6	−2.0

Showing differences in gene transcript abundance in the *expI* mutant compared with the wild type *P. atrosepticum* strain at 12 and 20 hours post inoculation. Based on a 1.5 fold statistically significant difference [*P* value <0.05] in expression. F/C = Fold change; negative value for fold change = genes showing reduced transcript abundance in the *expI* mutant; ^*^ = qRT-PCR data available (see Table S1); ^§^ = Regulators previously shown to be quorum sensing controlled in *Pectobacterium*; - = no observed change in expression by microarray analysis. Prot. = protein.

Other genes previously shown to fall under QS control *in vitro*, including *svx*, *nip* and a gene of unknown function (ECA3946), as well as three regulators (*expR* [ECA0106], *rsmA* [ECA3366] and *virR* [ECA1561]) involved in the production of PCWDEs [9], [10], [12]–[14], also showed reduced transcript abundance in the *expI* mutant compared to the wild type strain. This again justifies our approach in assessing the genome-wide effects of QS regulation during the potato interaction. While 1167 genes, representing a variety of processes, were found to be differentially expressed in the microarray experiment (Table S2), we focus predominantly on those that display reduced transcript levels in the mutant (as these are presumably induced directly or indirectly by QS), and which also have a known or putative role in virulence (Table 1).

### Secretion Systems

To successfully cause disease *Pba* must secrete a multitude of PCWDEs and other proteins, many of which are under QS control. We observed that both Type I and Type II secretion systems (T1SS and T2SS, respectively), which can be considered as ‘accessory virulence factors’, are modulated by QS (Table 1). Prior to this study the secretion systems responsible for the delivery of these virulence factors had not been reported as QS-regulated, and this observation indicates a novel facet to QS control of pathogenesis in pectobacteria.

**Figure 3:**
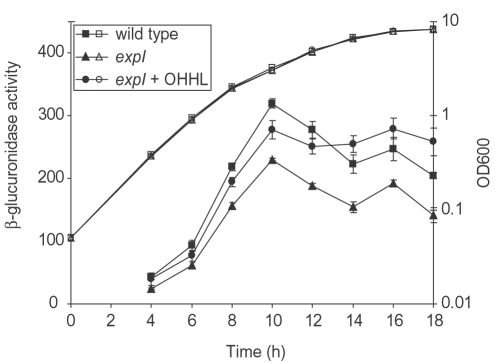
Expression of *outD-gusA in vitro* is QS-dependent. β-glucuronidase activity from an *outD-gusA* reporter fusion was measured in a wild type *P. atrosepticum* background (wild type, squares), in an *expI* mutant (*expI*, triangles) and in an *expI* mutant with the addition of exogenous 1 µg/ml OHHL (*expI*+OHHL, circles) throughout growth in PMB. β-glucuronidase activity (solid symbols) is expressed as A405/min/ml/OD600 and growth was measured as OD600 (open symbols). Bars show mean +/− standard error of the mean.

The T2SS is well characterised in pectobacteria and is responsible for secretion of many key virulence factors, *e.g.* Pel, Cel and Svx [10]. The T2SS of pectobacteria is encoded by a cluster of 15 *out* genes (ECA3098-3110 and ECA3113-3114) [20],[29], of which six (*outMLHGFD*) (by microarray analysis) exhibited reduced transcript abundance levels in the *expI* mutant (Table 1). Analysis of these and seven other *out* genes by qRT-PCR confirmed that all were expressed at a lower level in the *expI* mutant (Table S1), implying that expression of the Out T2SS is up-regulated by QS *in vivo*. Similar QS modulation of *out* expression was also demonstrated by both qRT-PCR (Table S1) and the use of an *outD-gusA* reporter fusion *in vitro* (Fig. 3). In the latter experiment, expression of *outD* (ECA3109) was reduced in the *expI* mutant and restored to wild type levels by the exogenous addition of OHHL, confirming QS modulation of *out* gene expression.

Regulation of the major secreted protease, PrtW, is QS-dependent in *Pba* [10]. Secretion of Prt by the PrtDEF T1SS is well-characterised in *Dickeya dadantii* (formerly *Erwinia chrysanthemi*) [30] and, by analogy, PrtW is expected to be secreted by the T1SS encoded by the neighbouring *prtDEF* (ECA 2781-2783) genes in *Pba*. To support this, the microarray data indicated that transcription of the T1SS genes *prtDF* was reduced in the *expI* mutant. Use of qRT-PCR confirmed that expression of all three T1SS genes, *prtDEF*, was significantly reduced in the *expI* mutant compared to the wild type (Table S1). QS-dependence of the T1SS and T2SS is a logical accompaniment to the simultaneous QS-dependent induction of their substrates, presumably allowing the systems to cope efficiently with the greatly increased quantity of these substrates. Examples of QS-modulated secretion have been reported previously in other pathogens, e.g. the Xcp T2SS of *Pseudomonas aeruginosa* and the Lip T1SS of *Serratia marcescens* [31],[32], although this is the first time that QS-dependant secretion systems have been described in pectobacteria.

As well as physically attacking the plant cell wall through the action of PCWDEs, in *Pba*1043 the Type III secretion system (T3SS) is also necessary for full virulence [5]. The T3SS is found in many Gram-negative pathogens of both animals and plants and is used to translocate effector proteins into host cells, where they manipulate host defences. Helper proteins (or harpins) are secreted to the extracellular environment, and may assist in effector translocation [33]. We observed that expression of the T3SS structural, putative effector and helper genes, and Type III-associated regulators were all modulated by QS. In *Pba*1043, and other pectobacteria, the T3SS is encoded by the *hrp* cluster, composed of around 40 CDSs. These CDSs encode components of the structural apparatus, as well as the putative effector DspA/E [ECA2113], and helpers HrpN [ECA2103] and HrpW [ECA2112]. The *Pba*1043 hrp cluster also contains a group of CDSs (ECA2104-ECA2110), which includes a number of lipoproteins, that appear to be absent in closely-related species [5]. ECA2104 shows homology to *vgrG* and is described below. In the microarray experiment, two CDSs *hrpE* [ECA2097], associated with the Type III structural apparatus, and a putative lipoprotein (ECA2108) exhibited decreased transcript abundance in the *expI* mutant (Table 1). qRT-PCR analysis of these and an additional 17 CDSs subsequently confirmed that CDSs encoding the Type III structural apparatus, the putative effector *dspE*, helpers *hrpN* and *hrpW*, regulators *hrpL*, *hrpS* and *hrpY*, and all CDSs between ECA2104 and ECA2110 were significantly reduced in the *expI* mutant compared to the wild type, predominantly at 12 h (Table S1). Either positive or negative QS regulation of the T3SS has been observed in other pathogens, e.g. *Pseudomonas aeruginosa* [34], *Vibrio harveyi* [35], enteropathogenic *E. coli* [36], *Ralstonia solanacearum* [37], and QS regulation of *hrpN* has been shown in *Pba* [8]. However, this is the first published evidence that QS plays a role in regulating the entire T3SS and its effectors in the enterobacterial plant pathogens, indicating that co-ordinated physical (PCWDEs) and stealth (T3SS) attacks may be necessary for successful disease development.

Recently, a novel T6SS was described and implicated in pathogenicity in *Vibrio cholerae* and *P. aeruginosa* [23],[38]. In *V. cholerae*, the system is encoded by the VAS locus, genes VCA0107-VCA0123. This locus is one member of a group of conserved gene clusters that are conserved in several pathogens. In both *V. cholerae* and *P. aeruginosa*, the T6SS is required for secretion of HcpA and VgrG proteins, although whether these represent putative effectors or simply secreted components of the secretion machinery is not yet clear [23],[38]. In *Pba*1043, the locus ECA3445–ECA3427 is predicted to encode a VAS-like T6SS and its putative substrates, since these genes encode proteins very similar to those encoded by VCA0107-VCA0123 and is similarly arranged on the chromosome. Microarray analysis indicated that 11 of the 18 genes were expressed at significantly lower levels in the *expI* mutant (Table 1), and so transcription of the T6SS also appears to be QS-dependent. The modulated genes included *Pba* homologues of VCA0120, VCA0116 and VCA0110, which are required for Type VI secretion in *V. cholerae* and/or *P. aeruginosa* [23],[38]. Moreover, the expression of several predicted T6SS substrates, *i.e.* encoded by *hcpA* and *vgrG*-like genes, was also found to be QS-dependent (Table 1). There are seven *hcpA* homologues in *Pba*, three of which (ECA4275[*hcp1*], ECA3428[*hcp2*] and ECA2866[*hcp3*]) are highly similar [20],[27]. ECA3428 and ECA4275 are sufficiently similar that it was not possible to design probes specific to each locus. Nevertheless, the probe detecting expression of both these genes showed decreased transcript abundance in expression in the *expI* mutant, indicating QS-dependent regulation. Expression of ECA2866 and four other homologues (ECA0456[*hcp4*], ECA3672, ECA0176 and ECA4277) was also decreased (Table 1). A combination of microarray analysis and qRT-PCR indicated reduced transcript abundance in the *expI* mutant of all five *vgrG* homologues, ECA2867, ECA3427, ECA2104, ECA4142 and ECA4276 in the *Pba*1043 genome (Table S1). Previous work showed that Hcp1-4 and a VgrG homologue (ECA3427) were found in the secretome of *Pba*1043. Over-expression of Hcp1 increased virulence, suggesting that this and related proteins are virulence factors in pectobacteria [27]. The VAS-like T6SS genes, ECA3445-ECA3427, appear to constitute an operon that may extend for a further seven CDSs (ECA3426-ECA3420). Of these, expression of six was reduced in the *expI* mutant (Table S2), raising the possibility that they may encode T6SS-dependent effectors.

**Figure 4:**
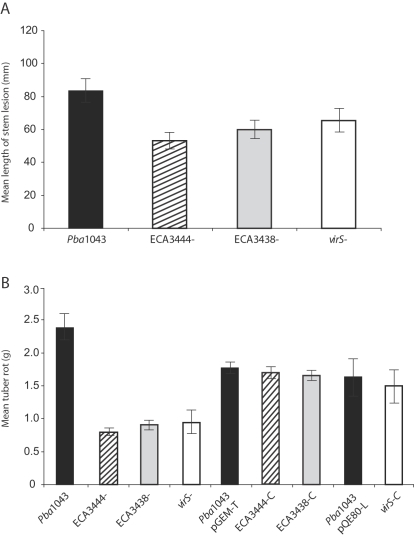
The Regulator *virS* and Components of the Type VI Secretion System are Involved in Virulence. Virulence assays in potato stems (A) and potato tubers (B), following inoculation of wild type *P. atrosepticum* and mutants affected in *virS* and Type VI secretion (ECA3444 and ECA3438). Complementation of tuber rotting phenotype using plasmids pGEM-T (ECA3444 and ECA3438) and pQE80-L *(virS)* (B). “C” indicates complemented. Bars show mean +/- standard error of the mean.

As the T6SS is clearly important for virulence in other pathogens, and a predicted substrate (Hcp1) affects virulence in *Pba*, we investigated whether the putative T6SS plays a role in virulence in *Pba*. Mutants in ECA3438 and ECA3444, when tested in potato stem and tuber virulence assays, both showed significantly reduced virulence compared with the wild type (Fig. 4). In tuber tests, complementation of the mutants *in trans* was shown to return virulence to wild type levels (Fig. 4B). Our results indicate, for the first time in any pathogen, a role for QS in the regulation of the T6SS and its putative substrates. It also demonstrates that the T6SS in *Pba* plays a role in pathogenesis, which appears to act in conjunction with PCWDE, the T3SS and other virulence determinants during the QS process.

### Regulators

**Figure 5:**
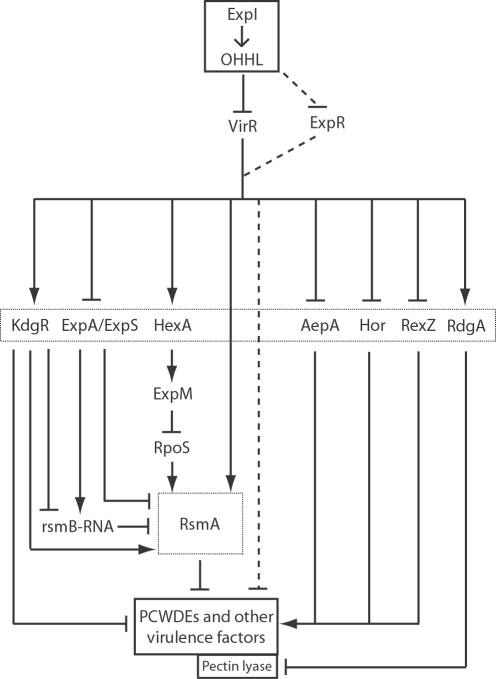
Schematic Model of the Hierarchical Relationships Between ExpI, Other Regulators and Virulence Factors in *Pectobacterium*. OHHL is believed to act *via* antagonism of the LuxR-family AHL-responsive transcriptional regulator, VirR, in turn activating or repressing numerous other regulators that control virulence factor gene expression. A second LuxR-type repressor, ExpR, may also contribute to OHHL regulation of downstream genes. This model represents an integrated summary of regulatory data derived from the study of multiple *Pectobacterium* strains, and is unlikely to apply in every aspect to all such strains. Adapted from von Bodman *et al*., [41] using additional information from Barnard and Salmond [16], and this study. Solid arrowheads indicate activation and bars indicate repression. Dotted lines indicate uncertain and/or strain dependent effects.

Microarray analysis revealed the QS-dependent differential expression of at least 79 CDSs with either known or putative regulatory functions (Table S2)**.** Twelve CDSs, five of which showed enhanced (*hexA* [ECA3030], *kdgR* [ECA2425], *phoP[pehR]* [ECA2445], *rdgA* [ECA2435] and *rsmA*) and seven of which showed reduced (*aepA* [ECA1022], *expA* [ECA2882], *expR, hexY* [ECA0809], *hor* [ECA1931], *rexZ* [ECA4123] and *virR*) transcript abundance in the *expI* mutant, are known to regulate PCWDEs production and are required for full virulence in pectobacteria [4], [12]–[14],[39] (Table 1). However, only three (*expR*, *rsmA* and *virR*) have previously been shown, *in vitro,* to fall under QS control [12]–[14]. Three CDSs (*hrpL* [ECA2087], *hrpY* [ECA2089] and *hrpS* [ECA2090]) involved in the regulation of the T3SS in pectobacteria and other phytopathogens [40] also showed decreased transcript abundance in the *expI* mutant. As all 15 of these CDSs are QS-dependent, this places QS at the apex of a regulatory hierarchy controlling both PCWDEs and the T3SS with its cognate effector proteins. Other QS-controlled regulators are also likely to be important during interaction with the plant (see below). Although QS is central to pathogenesis, elucidating the hierarchical relationships between “subordinate” regulators presents a particular challenge due to the lack of data on such relationships in this particular strain. Several virulence regulators in pectobacteria are known to operate though the Rsm system, which plays a major role in controlling virulence [14]. While not investigated as part of this work, it is highly likely that at least some of the regulators identified in this study operate through this system. Nevertheless, we have still been able to add considerable new information to existing regulatory models [41] and propose an extended model for virulence in the pectobacteria (Fig. 5).

In addition to regulators previously characterised in pectobacteria, differential expression of 18 further CDSs were found that are similar to a diverse range of transcriptional regulators in other bacteria (Table S2). These include CDSs with putative regulatory functions in nitrogen signal transduction and assimilation (*citB* [ECA2578], *glnB* [ECA3254], *nac* [ECA4483]), hydrogenase activity (*hypA* [ECA1235]), oxygen sensing (*fnr* [ECA2207]), defence against superoxides and other stress responses (*ohrR* [ECA3168], *phoB* [ECA1110], *recX* [ECA3368], *rseB* [ECA3282], *rseC* [ECA3281]), motility (*flgM* [ECA1700], *fliZ* [ECA1740]) and survival in soil (*sftR* [ECA4305]) (Table S2). Three of these additional regulators (*fliZ, ohrR* and *rscR*) have been implicated in virulence in other bacterial pathogens (Table 1) [42]–[44]. However, it does not necessarily follow that homologous regulatory proteins in bacteria are responsible for regulation of homologous processes [45].

Many CDSs encoding putative regulators of unknown function were shown to be regulated by QS. These CDSs thus represent novel candidates for virulence factors. Expression of one such CDS, ECA1562, subsequently named *virS*, was enhanced in the *expI* mutant at 12 and 20 hpi and is thus proposed to be repressed by QS (Table 1). VirS is a predicted TetR-family transcriptional regulator whose target(s) is unknown, although its closest reported homologue is a TetR family regulator, TvrR, implicated in virulence in the plant pathogen *Pseudomonas syringae* pv. tomato [46]. *virS* is located adjacent to the gene encoding a key QS-controlled regulator, VirR (ECA1561, [12]). However, inactivation of *virS* does not affect transcription of *virR* (data not shown). In order to determine whether *virS* plays a role in virulence, a defined *virS* mutant was constructed and tested in stem and tubers virulence assay. The *virS* mutant showed significantly reduced lesion formation compared with the wild type (Fig. 4) and is thus a novel virulence factor in *Pba*. In tuber tests, complementation of the mutant *in trans* returned virulence to wild type levels (Fig. 4B). The precise role of *virS in planta* is under investigation.

### Phytotoxins

The microarray data revealed a small reduction in expression of genes *cfa2* (ECA0607) and *cfa8A* (ECA0601) in the *expI* mutant compared to the wild type (Table 1). These genes are of particular interest as they are part of a cluster responsible for the synthesis of coronafacic acid (CFA) which, in *Pseudomonas syringae*, is a component of the phytotoxin coronatine [47]. We showed previously that mutations in this cluster (*cfa6* [ECA0603] and *cfa7* [ECA0602]) significantly reduce pathogenicity of *Pba*1043 on potato stems [20]. Transcriptional changes in *cfa2*, *cfa6* and *cfa7,* compared to a QS up-regulated (*pelA*) control, were thus examined at 12 and 20 hpi using qRT-PCR. At both time-points, *pelA*, the *cfa* genes, and the *cfl* (ECA0609) gene (involved in the formation of coronafacoyl conjugates by ligation of amino acids to CFA) showed reduced expression in the *expI* mutant, indicating that they are all under QS control (Table S1).

Salicylic acid (SA) and jasmonic acid (JA) are signalling molecules that play major roles in the activation of plant defences against pathogen attack [48]. CFA and its amino acid conjugates appear to act as structural and functional analogues of JA and its conjugates [49]. Recent work by Uppalapati *et al.* [21] showed that *Pseudomonas syringae* DC3000 mutants lacking CFA and/or coronatine were impaired in their ability to persist in tomato plants at the later stages of infection, and that the ability to persist coincided with the activation of JA-based, and concomitant suppression of SA-based, defences. It is hypothesised that, through this suppression of SA-mediated defences, coronafacoyl conjugates may aid *P. syringae* to enter the necrotrophic phase of infection and promote disease symptoms. It would appear therefore that *Pba*, through QS, synthesises CFA and coronafacoyl conjugates co-ordinately with multiple PCWDEs, the T3SS and T6SS in a synchronised assault on the plant as it progresses from biotrophy to necrotrophy. Although the effect of *Pba*-encoded CFA conjugates on plant defences has yet to be determined, such a two-pronged attack may be necessary for *Pba* to establish disease. It will be interesting to determine whether QS plays a similar role in *P. syringae* and related pathogens.

### Conclusions

QS regulation in pectobacteria was observed originally *in vitro* through dramatic impacts on PCWDE production, pathogenesis and (in *Pcc*) carbapenem antibiotic production [16]. The microarray analysis of global gene expression *in planta* presented here indicates a far broader physiological impact of QS, uncovering effects on the expression of many other genes associated with pathogenesis, and on other physiological processes not necessarily connected to plant pathogenesis. As QS is AHL concentration-dependent, its impact is likely to be greatest towards the latter stages of infection, where large quantities of PCWDEs are induced to attack plant cells and the characteristic soft rot disease symptoms occur [4]. Correspondingly, we find that production of the T1SS and T2SS, which are involved in the secretion of PCWDEs to the extracellular environment, are also under QS control.

A very small number of virulence regulators have previously been shown to fall under QS control. Our study has added over 70 other regulators to this list, including the major known virulence regulators associated with PCWDE production in pectobacteria. An important inference from these microarray analyses is that the QS control system occupies a critical position in the regulatory hierarchy and that multiple downstream regulators, some which may operate through the Rsm system [14], are under QS control. Furthermore, QS is seen to have both positive (activation) and negative (repression) effects on its downstream targets. Our knowledge of the hierarchical chain of command in control of the complex regulatory systems of PCWDE and other virulence factors is fragmentary, in part because the current literature describes experimental data derived from multiple strains of *Pba*, *Pcc*, and *Dickeya* spp. It may not therefore be completely legitimate to assume that the identified regulators play conserved roles in these different bacterial strains [45]. Nevertheless, while accepting this caveat, our results are consistent with the notion that, within the infected potato plant, QS acts as a key “master regulator” sensory system in this phytopathogen (Fig. 5). Additionally, regulators associated with virulence in other bacteria, and many novel putative regulators have also been identified; including VirS, which has been associated with virulence in this study.

In addition to the T1SS and T2SS, we have identified a T6SS in *Pba* and shown, for the first time in a plant pathogen, that it has a role in virulence. Moreover, we have described the first example of a QS-controlled T6SS in any pathogen. The precise functional roles of Hcp, VgrG and other possible Type VI substrates is unknown, but their proposed functions as effector proteins may be important for manipulating host defences whilst PCWDEs mount a simultaneous physical attack on plant cell walls. This does appear to be the case for both the T3SS and associated effectors, and coronafacoyl-amide conjugates, which are similarly QS-dependent, and consequently may suppress or otherwise manipulate defences. This has important implications for the infection process in pectobacteria, as it suggests that these pathogens do not infect merely by “brute force”, where the action of PCWDEs alone is sufficient to overwhelm plant defences and break down plant cell walls towards the end of infection. It seems increasingly likely that, in conjunction with PCWDEs, the production of virulence determinants that actively suppress plant defences, may be necessary to facilitate the transition from biotrophy to necrotrophy during disease development.

## Materials and Methods

### Bacterial strains, media and pathogenicity assays

*Pba*1043 [20], and strains with mutations in *expI*, ECA3438, ECA3444 and *virS* were used in this study. The *expI* mutant was derived from phage M1-mediated transduction of *expI*::mTn*5gusAgfp* from mutant MC3 into the wild type strain [10]. Mutants ECA3438 and ECA3444 were isolated from a mutation library of *Pba*1043 [5]. For inactivation of *virS*, 1085 bp of *virS* and surrounding regions were PCR-amplified using primers SC51 (ATTTGGATCCGTTGTTCCTGTTCTGTCG) and SC52 (TATATCTAGAGTTTACTGAGCAAGCGACG) and cloned into pBluescript-II KS^+^ using *Bam*HI-*Xba*I sites. The Kn^R^ cassette from pACYC177 (NEB) was cloned into the *Nsi*I site in the middle of *virS*. The resulting *virS*::Kn^R^ fragment was then cloned into the suicide vector, pKNG101 [50], generating the marker-exchange plasmid. The plasmid was introduced into *Pba*1043 by conjugation and transconjugants, resulting from integration of the suicide plasmid into the chromosome by homologous recombination, were selected by ability to grow on minimal medium containing 0.2% glucose+streptomycin. Following overnight growth in the absence of antibiotic selection, exconjugants, in which resolution of the plasmid from the chromosome leaving only the disrupted allele had occurred, were selected by ability to grow on minimal medium containing kanamycin+10% sucrose as sole carbon source and inability to grow on streptomycin. The disruption of the locus was confirmed by PCR analysis and DNA sequencing. All strains were maintained on Luria Bertani (LB) agar supplemented with kanamycin (50 µg/ml) and, unless stated otherwise, were cultured in 10 ml LB broth at 27°C overnight with aeration. Mutations were transduced into a clean *Pba*1043 background using phage M1 [51]. Pathogenicity tests were performed both on potato stems and tubers [19]. Approx. 10^2^ and 10^4^ cells per inoculation site were used for stems and tubers, respectively. Complementation of mutant strains was carried out *in trans* following cloning of ECA3444, ECA3438 and *expI* into plasmid pGEM-T (Promega, Southampton, UK) and *virS* into pQE80-L (Qiagen, Crawley, UK) with their own ribosome binding sites. OHHL-producing transgenic potato plants used for *in planta* OHHL complementation are as described [19]. GENSTAT for Windows was used for statistical analyses [20].

### *In planta* RNA preparation and microarrays

Wild type and *expI* mutant strains were grown in 10 ml LB broth at 27°C with aeration to stationary phase (approx. 1.0×10^9^ cells/ml) and re-suspended in 10 mM Mg SO_4_ prior to inoculation into sterilized potato tubers (2×10^7^ cells/ml into cv Maris Piper). The tubers were then wrapped in cling film and placed in a tray with wetted tissue to retain high humidity before incubation at 19°C in the dark. At 12 and 20 hours post inoculation (hpi), the bacterial cells were isolated from the tuber by scraping infected tissue into sterilised water. Starch was removed by centrifugation twice at 1000 rpm for 1 min. The bacterial cells in the supernatant were transferred to RNA stabilization buffer containing 1% phenol (pH 4.3, v/v) and 20% ethanol (v/v) and incubated on ice for at least 30 min. Total RNA was isolated using the SV Total RNA Isolation System (Promega) as described by the manufacturer and quantified using a NanoDrop ND-100 spectrophotometer (NanoDrop Technologies, Wilminton, DE). The quality of RNA was analyzed using an Agilent Bioanalyzer 2100 electrophoresis system (Agilent Technologies Inc., West Lothian, UK). In total 12 µg RNA was reverse transcribed and cDNA labelled [52]. 60-mer oligonucleotide probes were designed to *Pba* CDSs and used, together with controls, to generate 11K custom arrays with 99.5% genome coverage (Agilent, Inc., Santa Clara, CA, USA) [53]. Microarrays were carried out in triplicate for each time point [52],[53]. All microarray images were visually assessed for quality prior to feature extraction, whereby standard probe QC standards were applied (see further information in ArrayExpress-http://www.ebi.ac.uk/microarray-as/aer/). Features flagged as poor were removed prior to importing into Genespring software. Box plots and principle components analysis of whole datasets were used to assess array to array variation. Any outlying microarrays were repeated as necessary.

### Data analysis

Microarray data were analysed using GeneSpring software (version 7.2) and normalized using the Lowess algorithm (Agilent Technologies Inc.). Gene expression was considered to be different between the wild type and *expI* mutant strains for a probe if there was at least 1.5 fold change in normalised hybridisation score, and that change showed a statistically significant (Student's *t* test: *P* value <0.05) difference in their normalized data. Microarray data were submitted to the ArrayExpress repository (http://www.ebi.ac.uk/microarray-as/aer/), submission E-TABM-384, including details of the SCRI *Pectobacterium atrosepticum* 11k array (submission A-MEXP-942).

### RT-PCR and Gus expression

qRT-PCR was performed using *recA* as an endogenous control to validate differences in expression of genes identified from the microarray experiment, and to test additional genes in the *Pba* genome. RNA samples were analysed in triplicate. 5 µg total RNA was used to synthesize cDNA and 1 µl diluted template DNA (1:10) was used in a reaction of 25 µl containing 1x SYBR Green PCR Master Mix (Qiagen) and 10 pmol of the appropriate primers. qRT-PCR data were analysed using the Relative Expression Software Tool [REST] 2005 (Corbett Life Science, Cambridge, UK). Selected mutants were complemented by the addition of OHHL (Sigma) in DMSO (or with DMSO alone as a control) to PMM media at a final concentration of 5 µM, and strains grown to a final cell density of 5×10^7^ cfu/ml. After 18h incubation, bacteria were harvested and RNA extracted, purified and quantified as previously described. Differential expression was considered statistically significant if the *t*-test *P*-value was <0.05. To analyse gene expression of the *out* gene cluster *in vitro*, cultures were grown in Pel Minimal Medium (PMM) at 27 °C, RNA samples were prepared and qRT-PCR analysis performed as described in Burr *et al*., [12]. The *outD-gusA* strain, MC4, was as described by Corbett *et al*., [10], an *outD-gusA*/*expI* double mutant was generated by generalised transduction (data not shown), and β-glucuronidase (GusA) activity was measured throughout growth in Pel minimal broth (PMB) as described [10]. All primers used are described in Table S1.

### Bacterial counts and measurement of OHHL

Bacterial cells for counting were collected in 10 ml sterile water prior to dilution and plating as described [54]. Bacterial cells used for measuring OHHL levels were taken from these samples prior to dilution. OHHL levels were analysed using *E. coli* JM109 carrying a bioluminescence reporter vector (pSB401) [55]. Each sample of 100 µl was aliquoted into three wells of a sterile black 96-well microtitre plate, and 100 µl of the sensor strain (grown to an OD_600_ of 1.0) was added to each well. The microtitre plate was incubated at 37°C for 3 hours and the luminescence from each well was measured using a SpectraMax M5 luminescence plate reader at the default setting with an integration time of 1 second (Molecular Devices Corp., Sunnyvale, CA). A series of OHHL standards was used both as a positive control and to determine the level of OHHL.

### Identifiers, names and accessions numbers of genes referred to in the text

**ECA0105** (*expI*), YP048233; **ECA0106** (*expR*), YP048234; **ECA0176**, YP048830; **ECA0456** (*hcp4*), YP048574; **ECA0601** (*cfa8A*), YP048718; **ECA0602** (*cfa7*), YP048719; **ECA0603** (*cfa6*), YP048720; **ECA0607** (*cfa2*), YP048724; **ECA0609** (*cfl*), YP048726; **ECA0809** (*hexY*), YP048920; **ECA0931** (*svx*), YP049040; **ECA1017** (*pmeB*), YP049124; **ECA1022** (*aepA*), YP049129; **ECA1094** (*pel-3*), YP049200; **ECA1095** (*pehA*), YP049201; **ECA1110** (*phoB*), YP049216; **ECA1190** (*pehN*), YP049296; **ECA1235** (*hypA*), YP049341; **ECA1499** (*pnl*), YP049604; **ECA1561** (*virR*), YP049663; **ECA1562** (*virS*), YP049664; **ECA1700** (*flgM*), YP049801; **ECA1740** (*fliZ*), YP049840; **ECA1931** (*hor*), YP050028; **ECA1981** (*celV*), YP 050075; **ECA2087** (*hrpL*), YP050182; **ECA2089** (*hrpY*), YP050184; **ECA2090** (*hrpS*), YP050185; **ECA2097** (*hrpE*), YP050792; **ECA2103** (hrpN), YP050198; **ECA2104** (vgrG), YP050199; **ECA2108**, YP050203; **ECA2105-2110**, YP050200-050205; **ECA2112** (*hrpW*), YP050207; **ECA2113** (*dpsA/E*), YP050208; **ECA2207** (*fnr*), YP050300; **ECA2220**, YP050313; **ECA2402** (*pelW*), YP050497; **ECA2425** (*kdgR*), YP050520; **ECA2435** (*rdgA*), YP050530; **ECA2445** (*pehR*), YP050539; **ECA2553**, YP050644; **ECA2578** (*citB*), YP050669; **ECA2724** (*rscR*), YP050815; **ECA2781** (*prtF*), YP050872; **ECA2782** (*prtE*), YP050873; **ECA2783** (*prtD*), YP050874; **ECA2785** (*prtW*), YP050876; **ECA2827** (*celB*), YP050918; **ECA2866** (*hcp3*), YP050957; **ECA2867** (*vgrG*), YP050958; **ECA2882** (*expA*), YP050973; **ECA3030** (*hexA*), YP051120; **ECA3087** (*nip*), YP051177; **ECA3098-3114**, YP051188-051204; **ECA3100** (*outM*), YP051190; **ECA3101** (*outL*), YP051191; **ECA3105** (*outH*), YP051195; **ECA3106** (*outG*), YP051196; **ECA3107** (*outF*), YP051197; **ECA3109** (*outD*), YP051199; **ECA3168** (*ohrR*), YP051257; **ECA3254** (*glnB*), YP051343; **ECA3281** (*rseC*), YP051370; **ECA3282** (*rseB*), YP051371; **ECA3366** (*rsmA*), YP051455; **ECA3368** (*recX*), YP051457; **ECA3420**, YP051511; **ECA3421-3426**, YP051512-051517; **ECA3427**, YP051518; **ECA3428** (*hcp*), YP051519; **ECA3430**, YP051520; **ECA3432** (*vasK*), YP051522; **ECA3433**, YP051523; **ECA3436** (*vasG*), YP051526; **ECA3438**, YP051528; **ECA3440**, YP051530; **ECA3442** (*vasA*), YP051532; **ECA3443**, YP051533; **ECA3444**, YP051534; **ECA3445**, YP051535; **ECA3427-3445**, YP051518-051535; **ECA3646** (*celH*), YP051234; **ECA3672**, YP051760; **ECA3946**, YP052033; **ECA4067** (*pelA*), YP052154; **ECA4068** (*pelB*), YP052155; **ECA4069** (*pelC*), YP052156; **ECA4070** (*pelZ*), YP052157; **ECA4123** (*rexZ*), YP052210; **ECA4142** (*vgrG*), YP052229; **ECA4275**, YP052362; **ECA4276** (*vgrG*), YP052363; **ECA4277**, YP052364; **ECA4305** (*sftR*), YP052392; **ECA4483** (*nac*), YP052566.

## Supporting Information

Figure S1 ExpI is Involved in Virulence. Virulence assays in potato tubers following inoculation of wild type *P. atrosepticum* and the *expI* mutant, and complementation of the *expI* mutant by plasmid pGEM-T containing the *expI* coding region against the wild type containing empty vector. “C” indicates complemented. Bars show mean +/− standard error of the mean.
(0.39 MB EPS)

Table S1 qRT-PCR analysis of selected genes from *Pectobacterium atrosepticum* during potato infection. qRT-PCR data and primers of QS regulated genes.
(0.23 MB DOC)

Table S2 Microarray analysis of genes from *Pectobacterium atrosepticum* during potato infection. Expression values for microarray data of QS regulated genes.
(2.22 MB DOC)
