# Supplementary material for: Quorum Sensing Coordinates Brute Force and Stealth Modes of Infection in the Plant Pathogen Pectobacterium atrosepticum
Source: PLoS Pathog. 2008 Jun 20;4(6):e1000093. doi: 10.1371/journal.ppat.1000093 (PMC2413422; doi:10.1371/journal.ppat.1000093)
Supplement: Table S1 — qRT-PCR analysis of selected genes from Pectobacterium atrosepticum during potato infection. qRT-PCR data and primers of QS regulated genes. (0.23 MB DOC) [file ppat.1000093.s002.doc]

| **Supporting Table 1**. **qRT-PCR analysis of selected genes from *Pectobacterium atrosepticum* during potato infection** | | | | | | |
| --- | --- | --- | --- | --- | --- | --- |
| **Identifier** | **Gene name** | **Predicted function** | **RT-PCR**  **12hpi** | **RT-PCR**  **20hpi** | **RT-PCR**  ***in vitro*** | **Primers (5’ to 3’)** |
| **Plant cell wall degrading enzymes** | | | | | | |
| ECA1094 | *pel-3* | Pectate lyase | -45.6 | -24.9 |  | F: CCAACCTGGGTAAAACCATGA  R: CCGCCAGGGCCATTG |
| ECA4067 | *pelA* | pectate lyase I | -192.2 | -182.9 |  | F: GGAGGTTATGCCACCACTGA  R: TTGATCAGCGCATCTTCATT |
| ECA4068 | *pelB* | Pectate lyase II | -28.6 | -11.4 |  | F: GGCAGCCACTGGATTGTTAT  R: AGGTGATGATGAGCGGGTAA |
| ECA4069 | *pelC* | Pectate lyase III | -22.2 | -22.5 |  | F: GGGGTTACGCTACCACTGAC  R: CTGGCCGCAGATATTGTTTT |
| ECA4070 | *pelZ* | pectate lyase | -26.4 | -34.8 |  | F: GCAGCACCAGAGTTGAAAGG  R: GCCAACCTCGAAAACGATAA |
| ECA2402 | *pelW* | Pectate disaccharide-lyase | -4.1 | -2.3 |  | F: TGGGTTGCTGCACTTGATAG  R: GCGTTCTTAGCCAATTCTGC |
| ECA4510 | *pelX* | exopolygalacturonate lyase | -5.6 | n |  | F: CCTAAAACCACCAGCGAACATT  R: CCCCCGCTTTTACCTGCTT |
| ECA3112 | - | Pectate lyase | -3.0 | -2.3 |  | F: CGGTAGCTATTGCCCTTTCA  R: AACGCGTTTTTCAATTCCTG |
| ECA1981 | *celV* | endoglucanase V | -17.1 | -12.3 |  | F: TATGCTGCCCATGAATTTGA  R: AATCACGTTCGGCGAGTTAC |
| ECA2220 | - | Putative cellulase | -7.5 | -6.9 |  | F: TGACTTGGTGGTAGCGTGT  R: CTGCGGTGCAATTTAGGAAT |
| ECA2827 | *celB* | Beta(1,4)-glucan glucanohydrolase | -29.1 | -37.9 |  | F: CATCCAAAGACGCAGAA  R: AACCTTTAACGCTGGAGCTG |
| ECA3646 | *celH* | 6-phospho-beta-glucosidase | -5.1 | n |  | F: AGGGCAGGATTCCTATTTGG  R: TCCAGCATAAAACGTGACCA |
| ECA2785 | *prtW* | Metalloprotease | -247.6 | -174.7 |  | F: AACCATGTTCGTGGTGATGA  R: ATTTCACAAAGCCCTGATCG |
| ECA1499 | *Pnl* | Pectin lyase | -22.8 | -50.2 |  | F: GTCGGGCGGTAAAGTTGTTA  R: CGAAAGAACCGACAATGGTT |
| ECA0107 | *pmeB* | Pectin methylesterase | -10.1 | -4.1 |  | F: CACGCTCCCGGACTATTTTA  R: CCCGGCAGCACTTTAATATC |
| ECA1095 | *pehA* | Endo-polygalacturonase | -10.3 | -4.9 |  | F: TTCACGAACAGTGAGCGAAC  R: CGCTGAGAAAGACGGAAGAG |
| ECA1190 | *pehN* | Putative polygalacturonase | -4.3 | -27.8 |  | F: TTCCTGCATCACACTGGGTA  R: CAGCGGATCGACCAGATAGT |
| ECA3111 | *pehX* | exo-poly-alpha-D-galacturonosidase | -8.0 | n |  | F: CTCTGTGCGACAGTCGTTCT  R: CCAGGCAAGTACCACGCTAT |
| **Type I secretion** | | | | | | |
| ECA2781 | *prtF* | Protease secretion | -3.8 | -5.2 |  | F: CTGTTTGGGCTGCTTTCTTC  R: TGCTGACGCTATCTGTTTGG |
| ECA2783 | *prtD* | Protease secretion ATP-binding | -5.6 | -11.7 |  | F: TCTGGAGCATTGGGATTTTC  R: GTACCGAGTCGGACGACAAC |
| ECA2782 | *prtE* | Protease secretion protein | -2.3 | -4.4 |  | F: AAAGGCGTCATGGTTAATGG  R: ACGTGCATCAACCTCATTCA |
| **Type II secretion** | | | | | | |
| ECA3098 | *outO* | Type IV prepilin-like proteins leader peptide processing enzyme | -2.0 | -2.5 |  | F: TGGGGCTGATTGTTGGTAGT  R: GCTGAACAGCGGGATGTTAT |
| ECA3099 | *outN* | General secretion pathway protein N | -5.6 | -4.0 | -8.5 | F: CCTGACGTGTTATGCACCTG  R: CGCGAATAATGCCTTTACCT |
| ECA3100 | *outM* | General secretion pathway protein M | -3.8 | -3.1 | -5.4 | F: GCAACTGCGGGAAGATC  R: CAAAATCGGCAGACTGGTT |
| ECA3101 | *outL* | General secretion pathway protein L | -4.6 | -4.0 |  | F: GAGGAACGGTTGTTCGACAT  R: ACGTCAGGGCATAAAACGTC |
| ECA3102 | *outK* | General secretion pathway protein K | -4.2 | -2.5 |  | F: GCACGGGTAAAGCGTTTATG  R: TAATCTGGCCGATGATTTCC |
| ECA3103 | *outJ* | General secretion pathway protein J | -4.7 | -2.5 |  | F: ACGCTGCTGGAAATGATTCT  R: GCGTGCAATCATCTGAGAAA |
| ECA3104 | *outI* | General secretion pathway protein I | -6.6 | -3.0 |  | F: GCTGGAAGAGAAGACGTTCG  R: TCTGAGGATCACCGGTCTCT |
| ECA3105 | *outH* | General secretion pathway protein H | -3.8 | -3.4 |  | F: TGCTGCTGAAAACAGTCAGG  R: GCAATAGTTCAAGCCGCAAT |
| ECA3106 | *outG* | General secretion pathway protein G | -8.8 | -5.5 | -62.5 | F: TCATTCTCGGCGTATTGG  R: TGTTATCCAGCTTGTACATATCG |
| ECA3107 | *outF* | General secretion pathway protein F | -4.5 | -10.1 |  | F: AGACAGGCGCGTCAGTTATT  R: ATCCAGTGCTTCTTCCAACG |
| ECA3108 | *outE* | General secretion pathway protein E | -4.1 | -3.4 |  | F: CCATATTGCCTTTCGCCTAT  R: TTGCCGCTCAAACTCTTCTT |
| ECA3109 | *outD* | General secretion pathway protein D | -7.6 | -4.9 | -9.3 | F: GTAATACACGCCGCAAGAG  R: GATCGATAATCACCGTCTTATTC |
| ECA3110 | *outC* | General secretion pathway protein C | -4.4 | -2.5 |  | F: ACTGCCAGCTCCTCTGATTC  R: ACGTTGTGGGCTTTTCTTTC |
| **Type III secretion** | | | | | | |
| ECA2082 | *hrpO* | Type III secretion protein | -3.86 | -2.21 |  | F: TGCGACAGGCCAGTCACA  R: TCCAGCGTTTGGCGTTGT |
| ECA2085 | *hrpI* | Type III secretion protein | -3.32 | n |  | F: GCTAAATCGTATTGCGCTGA  R: GCCAGTGGTTTAGGCAGGTA |
| ECA2093 | *hrpA* | Type III secretion protein | -4.88 | n |  | F: CGATGGCCGGTTCTCTGA  R: TCCAACGCAATTTTCTGTGC |
| ECA2094 | *hrpB* | Type III secretion protein | -6.57 | n |  | F: TTTAGTGCGGCGTTGTCATC  R: CGTTCAATCCCTGAGATTTTTCC |
| ECA2097 | *hrpE* | Type III secretion protein | -4.52 | n |  | F: TCTGGGAAGAAGCCACTGTACA  R: TGCATTTTCTACCGCCTGTTC |
| ECA2098 | *hrpF* | Type III secretion protein | -4.28 | n |  | F: TCAGCGCCGCCTTGAC  R: ACCGCCTTCGGCAACA |
| ECA2100 | *hrcC* | Type III secretion protein | -6.33 | n |  | F: CATTCTTACACCCCACCTTGTTG  R: GGTTCATGGCACCGTTCAA |
| ECA2101 | *hrpT* | Type III secretion lipoprotein | -6.11 | -2.51 |  | F: AGTGGTGTTCATCGCATTCTT  R: CGCTTACCCTGCTGTAATCC |
| ECA2103 | *hrpN* | Type III secretion protein | -1.66 | 2.53 |  | F: GGCGTATACCCAAGGCGTTA  R: AGCCTTGCAGACCGTTGTTAC |
| ECA2105 | *-* | Type III secretion protein | -2.42 | -2.56 |  | F: GGTAGATCGCTCGGCAGTAG  R: CGAACGACGTCAAGAGACTG |
| ECA2106 | *-* | Hypothetical protein | -7.96 | -2.5 |  | F: GCCTAAAAGCGACACTGACC  R: TGCCCGTCCTTCAATATTTC |
| ECA2107 | *-* | Putative lipoprotein | -1.85 | -1.68 |  | F: TCCACAAAATCGGCTTTTTC  R: ATAACGCTGGCCTGTGATTC |
| ECA2108 | *-* | Putative lipoprotein | -1.97 | -2.19 |  | F: CAATACGCGAACGGAATTTT  R: ACGGGAACACCATCAGTAGG |
| ECA2109 | *-* | Putative lipoprotein | -4.14 | n |  | F: CTGGGTCTTTGCGTTTCATT  R: ACACTGGCACCAGGTCCTAC |
| ECA2110 | *-* | Conserved hypothetical protein | -8.89 | n |  | F: AATTAGGCGATAGCGACACG  R: GTGGATGAACCACCGCTAAT |
| ECA2111 | *-* | Potential hrpW-specific chaperone | -7.44 | n |  | F: AGCAAGGCGGATACATTACG  R: GTCCTGCTCCAGAGAATCCA |
| ECA2112 | *hrpW* | Type III secretion protein | -4.37 | n |  | F: TGCAGGCAGTGCGTTAAATAA  R: CCAGCAGCGCTTTTAACAGATC |
| ECA2113 | *dspE* | Putative avirulence protein | -4.08 | n |  | F: CTCAGCCCAGTCTGTCCATT  R: CCCCTGACTCTTCACCTGAC |
| ECA2114 | *dspF* | Putative avirulence protein | -3.71 | n |  | F: CGCGTTGGTATTGCATAGTG  R: CGCCGTAAAACTTGGTTCAT |
| **Type VI secretion and putative substrates** | | | | | | |
| ECA3428/ECA4275 | *hcp1/hcp2* | Hemolysin co-regulated protein (HcpA) | -21.0 | -123.3 |  | F: ATTGAAGTGTCGCTGGCTTACC  R: TCATCGGAGCCGGACGT |
| ECA2866 | *hcp3* | HcpA homologue | -315.3 | -280.1 |  | F: TCGGCAACATCTATGTGGAA  R: GGTACCATTTCAGGGTCACG |
| ECA0456 | *hcp4* | HcpA homologue | -11.1 | 2.4 |  | F: ACCGCTAGTCCTAACCAGCA  R: TGGCAGGTGATGCTTTCATA |
| ECA0176 | *-* | HcpA homologue | -17.5 | 17.9 |  | F: ATCTGCTGAGTGTGGCACTG  R: GCCGTACGGTAAAAATCGAA |
| ECA3672 | *-* | HcpA homologue | -6.3 | 4.8 |  | F: CGCGATCAAAATGTTTCTCA  R: TACAATGCTCGCATCCGTTA |
| ECA1069 | - | HcpA homologue | -3.7 | -6.2 |  | F: ATGTTTCCCTCAACCCTGTG  R: AACGAAATGCTTTCCTGTGG |
| ECA2867 | *vgrG* | Putative RHS accessory genetic element | -102.0 | -200.0 |  | F: ACGTGGCAAACAGTACAGGA  R: AGTTCGCCTTCGTACCACAC |
| ECA3427 | *-* | VgrG-like protein | -22.7 | -38.5 |  | F: TACCGTAAAGGTCGGTGCTC  R: TCGCCTTGAGTAAACCCACT |
| ECA2104 | - | VrgG-like protein | -8.33 | n |  | F: ACCAACCGTCCTCCTTACGA  R: CTTCAAAGCGCAGCTCATTAAA |
| ECA4142 | *vgrG* | VgrG-like protein | -4.3 | -2.6 |  | F: CTGGATGAGGGGTTGAATTG  R: CCCTGCGTGAATCCACTAAT |
| ECA4276 | *vgrG* | Rhs-family protein | -166.7 | -66.7 |  | F: ACGGTAAAGGTCGGTGCAT  R: CTCACTCGGCGCTTTAGTTC |
| ECA3421 | - | Rhs protein | -10.4 | -4.2 |  | F: AGTAGTGCTGCTGCGGAAAA  R: TACCCACTTCCATCCGTTTG |
| ECA3422 | - | Conserved hypothetical protein | -27.8 | -14.1 |  | F: CTCAGGGTGGCTGTTTTGTT  R: CGTTAAGCGGGATGCAATAC |
| ECA3430 | - | Putative phospholipase | -22.7 | -10.6 |  | F: TGGTGAAGCGCAATATCAAC  R: GCCTAACCCTGCCTGATACC |
| ECA3432 | *vasK* | IcmF-like protein | -14.3 | -25.6 |  | F: GCGCTACTGTGGGTCGTAGT  R: GGATCCTGCTCAATCACCTG |
| ECA3436 | *vasG* | ClpB-like protein | -41.7 | -43.5 |  | F: TCCGTATTGAGCACCTGTTG  R: GCCAGCTGTCCTGTAACCAT |
| ECA3438 | *-* | Conserved hypothetical protein | -34.5 | -50.0 |  | F: GACACACCGATTACGCACTG  R: GGGCATCATCACCTGGAATA |
| ECA3439 | - | Putative lipoprotein | -55.6 | -43.5 |  | F: GTTACTGCTGTCGGGGTGTT  R: TCGTGATCTGGTCGTAATCG |
| ECA3441 | - | Conserved hypothetical protein | -52.6 | -52.6 |  | F: GACGTTCCGTCAGGATGTCT  R: TGGGGTGAAAGCCAATAGAG |
| ECA3442 | *vasA* | Conserved hypothetical protein | -38.5 | -90.9 |  | F: ACCCTGAGCTTACCCGATTT  R: GCAGGTAATTAGGCCACAGC |
| ECA3444 | *-* | Conserved hypothetical protein | -250.0 | -37.0 |  | F: GCGCTGGTTGCTAACATTCT  R: TCAGAGAACGCCAGGAAGAT |
| ECA3445 | *-* | Conserved hypothetical protein | -333.3 | -58.8 |  | F: AAATATGAAAGGGCGCACAG  R: AATTTCACTGGCAGGTCGTC |
| **Regulators** | | | | | | |
| ECA0105 | *expI*^§^* | N-acylhomoserine lactone synthesis | -43.4 | -85.8 |  | F: AGAAGCTTTGGCTCGTCGTATT  R: CGTAGAGGCCACTGCTTCAAT |
| ECA0106 | *expR*^§^* | Quorum-sensing regulator | -54.3 | -190.4 |  | F: GGTAAAATTTCATATCGGCAACGT  R: GTAACTCAACGCCGAGCCTTA |
| ECA1022 | *aepA** | virulence regulator | -8.8 | -3.0 |  | F: ACCAAAATTGGTAGCGATGC  R: GCATCCGTTGGAGGAAGATA |
| ECA1740 | *fliZ* | Alternative sigma factor regulatory protein | -2.3 | -5.2 |  | F: AAAAATCGATTGGCAGCATC  R: ATGACGAACTTCGGTTTGCT |
| ECA1931 | *Hor** | global regulator | -20.2 | -14.1 |  | F: TCTGATTTAGCCCGTCTGGT  R: TTCGGACTAATGAGGGTTGC |
| ECA2882 | *expA** | two-component response regulator | -4.6 | -1.6 |  | F: TGGCATTGAGCCAGTTGGA  R: AATCTGTAATTCGCGTTCAGACAA |
| ECA1562 | *(virS)* | TetR-family transcriptional regulator | +5.3 | n |  | F: GCCACCATGAATGAACTGG  R: GCACTTCCAGCGTCTCAGAT |
| ECA2425 | *kdgR** | Pectin degradation repressor | +2.4 | n |  | F: ATTCCGTGTCGTCCGTTTTA  R: GACATAGCCCAGGGATTTCA |
| ECA2724 | *rscR* | LysR-family transcriptional regulator | n | +2.0 |  | F: AGTCTTTGCCGAAGTCCTGA  R: AGACGGCCATGCTCATTAAC |
| ECA3168 | *ohrR* | Hydroperoxide resistance regulator | +2.0 | +3.7 |  | F: CCGTTTGCTGTCTGAGTTGA  R: TCTTCTGTTCCCCGATTACG |
| hexA | *hexA** | LysR-family transcriptional regulator of motility and virulence | n | +2.3 |  | F: CCAGTGCAAATCGACCAGTA  R: AGCTCCTTTCCGATGAGTTG |
| ECA3366 | *csrA / rsmA*^§^* | Global regulator | +1.9 | n |  | F: ACTCGTCGAGTTGGCGAAAC  R: ATACGCACCTGGTTGCCTTT |
| ECA3435 | - | Putative sigma-54 dependent transcriptional regulator | -18.9 | -24.4 |  | F: ACCCGACAGCATGATGAAAC  R: AGGTACGCGCCTGATTCTTA |
| ECA2435 | *rdgA** | regulator of pectin lyase production | +2.2 | n |  | F: TGGCGACATGATTGGTGTAT  R: TGTTGTCCCAAGCTGATACG |
| ECA2437 | *rdgB** | regulator of pectin lyase production | -3.6 | n |  | F: TGGATCCACAAACTGCTGAG  R: AGCTCATCTTTTCGCATGGT |
| ECA2087 | *hrpL* | Sigma factor | -1.57 | n |  | F: CAATCTGGTGCGCAACTATTTC  R: CTCGCTGGCTTTCGGTAATC |
| ECA2089 | *hrpY* | Two-component response regulator | -2.49 | n |  | F: CCGATTTTGGTGCTCAGCAT  R: ACCGCTTTTCAGGGCATG |
| ECA2090 | *hrpS* | Sigma-54-dependent enhancer-binding protein | -6.20 | n |  | F: CGCTGTTTCAGCGTTTTAGTCA  R: CATCGGCGGCAATGTCA |
| **Others** | | | | | | |
| ECA0607 | *cfa2* | Coronafacic acid dehydratase | -7.4 | -4.5 |  | F: TTATGGCTGTTTCGGGTCAGA  R: AACGGTGATGTTGAAAGCTGAA |
| ECA0603 | *cfa6* | Type I polyketide synthase | -10.3 | -3.7 |  | F: GCGGATGCATGGAGAGCTT  R: TGGCTTAAATAGCCAGAGGAATAGG |
| ECA0602 | *cfa7* | Type I polyketide synthase | -5.1 | -1.7 |  | F: GCCTGCTCTGCGTCTCTCA  R: GAACACTCGCCGCTCTTCAG |
| ECA0609 | *cfl* | Coronafacate ligase | -4.8 | -1.7 |  | F: TCGCTGGCGAACGTATTATT  R: CTGATTTTTCCCGTGGTGTT |
| ECA0931 | *svx* | Putative avirulence protein | -16.0 | -17.1 |  | F: ATACGGAAAGCGGTTCTTCA  R: ACTACGTTGGCTGGATTTGG |
| ECA3087 | *nip* | Virulence-associated protein | -66.9 | -83.5 |  | F: TTCGCCACTTTTTCTTCACA  R: TGATGGTTTCAACCCTCCAT |
| ECA3946 | - | Putative exported protein | -29.7 | -18.9 |  | F: GTTTCCGTGGAAGTGAAGGA  R: GGCATCAGTCTGTGCACTTG |
| ECA0852 | - | Putative exported hydrolase | -7.3 | -7.2 |  | F: GGTTGGGTAAAGCAATTGGA  R: AGCATCGTCCAGGTCGTATT |
| **Reference gene** | | | | | | |
| ECA3369 | *recA* | Recombinase A | 1.0 | 1.0 |  | F: AGTGGCAGCACCGTTCAAG  R: CACCCAGATCAACCAGCTCA |
| **Primer sequences for complementary clones** | | | | | | |
| ECA1562 | *virS* | TetR-family transcriptional regulator |  |  |  | F:TATAGAATTCTGGCAGGAGTGGTGATG  R:TATAGGATCCGGATAGAAAAGGAACCTGTG |
| ECA3444 | *-* | Conserved hypothetical protein |  |  |  | F: TGAAAGGCCCCCTCGGCAATATTC  R: ATCAAACAGACTTGCCGCGCTTC |
| ECA3438 | *-* | Conserved hypothetical protein |  |  |  | F:TAATTGATGTTTACTGACGAAATAGGGAAATAATATGTCGAGTCGCAATCGCATTATC  R: TGCTCATCTCTTACTCTCCTGACTAC |

**Supporting Table 1.** *P. atrosepticum* coding sequences in an *expI* mutant showing a statistically significant (P<0.05) change in transcript abundance (1.5 fold) compared to the wild type strain. qRT-PCR analysis of RNA from potato tubers at 12 and 20 hour post inoculation and, for selected sequences, following growth in Pel minimal broth.

*Regulation of virulence in *Pectobacterium*

§ Regulators previously shown to be under QS control in *Pectobacterium*

Plus value = increased transcript abundance in *expI* mutant

Minus value = decreased transcript abundance in *expI* mutant

n = no change

F = forward primer

R = reverse primer
