# Supplementary material for: Quorum Sensing Coordinates Brute Force and Stealth Modes of Infection in the Plant Pathogen Pectobacterium atrosepticum
Source: PLoS Pathog. 2008 Jun 20;4(6):e1000093. doi: 10.1371/journal.ppat.1000093 (PMC2413422; doi:10.1371/journal.ppat.1000093)
Supplement: Table S2 — Microarray analysis of genes from Pectobacterium atrosepticum during potato infection. Expression values for microarray data of QS regulated genes. (2.22 MB DOC) [file ppat.1000093.s003.doc]

| **Supporting Table 2. Microarray analysis of genes from *Pectobacterium atrosepticum* during potato infection** | | | | | | |
| --- | --- | --- | --- | --- | --- | --- |
| **Systematic** | **Normaliz-ed 12h** | **t-test P-value 12h** | **Normaliz-ed 20h** | **t-test P-value 20h** | **Common** | **Description** |
|  |  |  |  |  |  |  |
| ansB(ECAORF1075_1_sense) | 0.324813 | 0.018804 | 0.527031 | 0.023454 | ansB | None |
| basR(ECAORF4004_1_sense) |  |  | 1.455204 | 0.038377 | basR | None |
| basS(ECAORF4005_1_sense) | 0.485126 | 0.023708 |  |  | basS | None |
| ccdA(ECAORF0937_1_sense) | 0.554898 | 0.039453 |  |  |  | PROBE01823 |
| celA(ECAORF4370_1_sense) | 0.4715 | 0.029883 |  |  | celA | None |
| celC(ECAORF3098_1_sense) | 0.268371 | 0.00162 |  |  |  | PROBE06029 |
| cydA(ECAORF1338_1_sense) | 2.91563 | 0.007439 |  |  | cydA | None |
| cydB(ECAORF1339_1_sense) | 2.156201 | 6.75E-04 |  |  | cydB | None |
| cysG(ECAORF4041_1_sense) | 0.414382 | 3.17E-05 |  |  |  | PROBE07829 |
| doC(ECAORF0248_1_sense) | 0.557691 | 0.00116 |  |  | doC | None |
| ECA0001(ECAORF0001_1_sense) | 1.513937 | 0.0218 |  |  |  | PROBE00001 |
| ECA0009(ECAORF0009_1_sense) | 0.353773 | 8.61E-04 |  |  | ECA0009 | putative exported protein |
| ECA0009(ECAORF0009_3_sense) | 0.34456 | 0.021603 |  |  | ECA0009 | putative exported protein |
| ECA0010(ECAORF0010_1_sense) | 0.300295 | 0.04842 |  |  | rbsD | high affinity ribose transport protein |
| ECA0011(ECAORF0011_1_sense) | 0.329645 | 0.040496 |  |  | rbsA | ribose transport ATP-binding protein |
| ECA0012(ECAORF0012_1_sense) | 0.215795 | 0.001325 |  |  | rbsC | ribose transport permease system protein |
| ECA0013(ECAORF0013_1_sense) | 0.127825 | 8.88E-06 |  |  | rbsB | ribose-binding periplasmic protein |
| ECA0014(ECAORF0014_1_sense) | 0.459768 | 0.001466 |  |  | rbsK | ribokinase |
| ECA0022(ECAORF0022_3_sense) | 1.536509 | 0.041281 |  |  | engB | probable GTP-binding protein |
| ECA0026(ECAORF0024_1_sense) | 0.576309 | 0.046054 |  |  | hemN | oxygen-independent coproporphyrinogen III oxidase |
| ECA0028(ECAORF0026_1_sense) | 2.493613 | 9.70E-04 |  |  | glnL | nitrogen regulation two-component system, histidine kinase |
| ECA0030(ECAORF0028_1_sense) | 2.659047 | 0.002214 |  |  | typA | GTP-binding regulatory protein |
| ECA0030(ECAORF0028_3_sense) | 2.962878 | 0.018843 |  |  | typA | GTP-binding regulatory protein |
| ECA0036(ECAORF0034_1_sense) | 3.102068 | 0.004809 |  |  | recG | putative ATP-dependent DNA helicase |
| ECA0037(ECAORF0035_1_sense) | 2.020617 | 0.01673 |  |  | trmH | putative tRNA (guanosine-2'-O)-methyltransferase |
| ECA0038(ECAORF0036_1_sense) | 2.759662 | 0.042257 |  |  | spoT | guanosine-3',5'-bis(diphosphate) 3'-pyrophosphohydrolase |
| ECA0040(ECAORF0038_1_sense) | 2.134744 | 0.004236 | 1.51744 | 0.00107 | gmk | guanylate kinase |
| ECA0047(ECAORF0044_1_sense) | 1.841988 | 2.95E-05 |  |  | ECA0047 | putative exported protein |
| ECA0048(ECAORF0045_3_sense) | 1.619769 | 0.028698 |  |  | pitA | low-affinity inorganic phosphate transporter |
| ECA0054(ECAORF0051_1_sense) |  |  | 2.989845 | 0.010524 | ECA0054 | conserved hypothetical protein |
| ECA0058(ECAORF0055_1_sense) | 0.53361 | 0.037863 |  |  | ECA0058 | transmembrane sensor |
| ECA0061(ECAORF0058_1_sense) | 1.713332 | 0.028114 |  |  | ECA0061 | conserved hypothetical protein |
| ECA0069(ECAORF0066_1_sense) |  |  | 3.460512 | 1.64E-05 | padC | phenolic acid decarboxylase |
| ECA0078(ECAORF0074_1_sense) | 0.419451 | 0.001057 |  |  | tkrA | 2-ketogluconate reductase |
| ECA0084(ECAORF0080_1_sense) | 1.895982 | 0.049448 |  |  | glyQ | glycine-tRNA synthetase, alpha subunit |
| ECA0085(ECAORF0081_1_sense) | 2.311099 | 0.009329 |  |  | glyS | glycine-tRNA synthetase, beta subunit |
| ECA0086(ECAORF0082_1_sense) | 0.404463 | 0.015685 |  |  | ECA0086 | putative exported protein |
| ECA0087(ECAORF0083_3_sense) | 0.325571 | 0.005108 |  |  | mtlA | PTS system, mannitol-specific IIabc component |
| ECA0091(ECAORF0087_1_sense) | 0.465901 | 0.005261 |  |  | ECA0091 | methyl-accepting chemotaxis protein |
| ECA0092(ECAORF0088_1_sense) | 0.487776 | 0.035139 |  |  | sodA | manganese superoxide dismutase |
| ECA0100(ECAORF0096_1_sense) |  |  | 1.477647 | 0.017235 | xylH | xylose transport system permease |
| ECA0102(ECAORF0098_1_sense) | 0.102189 | 6.66E-04 |  |  | ECA0102 | conserved hypothetical protein |
| ECA0102(ECAORF0098_3_sense) | 0.127033 | 0.001797 |  |  | ECA0102 | conserved hypothetical protein |
| ECA0104(ECAORF0100_1_sense) | 0.187551 | 6.11E-04 |  |  | argG | argininosuccinate synthase |
| ECA0105(ECAORF0101_1_sense) | 0.03551 | 4.78E-04 | 0.054723 | 9.78E-04 | expI | N-acylhomoserine lactone synthesis protein |
| ECA0106(ECAORF0102_1_sense) | 0.106132 | 1.88E-04 | 0.117282 | 0.004615 | expR | quorum-sensing transcriptional regulator |
| ECA0122(ECAORF0118_1_sense) | 0.620102 | 0.015206 |  |  | ECA0122 | hypothetical protein |
| ECA0123(ECAORF0119_1_sense) | 0.415596 | 0.005384 | 1.555157 | 0.002648 | ECA0123 | putative lipoprotein |
| ECA0125(ECAORF0121_1_sense) | 0.471572 | 0.031193 |  |  | ECA0125 | conserved hypothetical protein |
| ECA0129(ECAORF0125_1_sense) | 0.233336 | 5.36E-05 |  |  | ECA0129 | putative deoxycytidylate deaminase |
| ECA0134(ECAORF0130_1_sense) | 1.532051 | 0.014167 |  |  | ECA0134 | putative peptidase |
| ECA0147(ECAORF0143_1_sense) |  |  | 1.543617 | 0.046187 | rpmG | 50S ribosomal protein L33 |
| ECA0151(ECAORF0147_1_sense) | 1.519297 | 0.008036 |  |  | kdtA | 3-deoxy-D-manno-octulosonic-acid transferase |
| ECA0159(ECAORF0155_1_sense) | 1.770103 | 0.049624 |  |  | waaG | lipopolysaccharide core biosynthesis protein |
| ECA0163(ECAORF0159_1_sense) |  |  | 1.834139 | 0.001685 | waaC | lipopolysaccharide heptosyltransferase-1 |
| ECA0173(ECAORF0168_1_sense) | 1.481099 | 0.00359 |  |  | gpsA | glycerol-3-phosphate dehydrogenase [NAD(P)+] |
| ECA0176(ECAORF0171_1_sense) | 0.046587 | 4.97E-06 | 0.079553 | 0.042292 | ECA0176 | HcpA homologue |
| ECA0176(ECAORF0171_3_sense) | 0.052821 | 0.002647 | 0.061477 | 0.015561 | ECA0176 | HcpA homologue |
| ECA0177(ECAORF0172_1_sense) | 0.132329 | 0.013631 | 0.105863 | 0.016354 | ECA0177 | conserved hypothetical protein |
| ECA0182(ECAORF0177_1_sense) |  |  | 0.493159 | 0.013239 | ECA0182 | methyl-accepting chemotaxis protein |
| ECA0184(ECAORF0179_1_sense) | 0.330963 | 0.040671 |  |  | ECA0184 | putative carboxymethylenebutenolidase |
| ECA0185(ECAORF0180_1_sense) | 0.443385 | 0.001156 |  |  | udp | uridine phosphorylase |
| ECA0186(ECAORF0181_1_sense) | 0.452415 | 0.020554 |  |  | ECA0186 | conserved hypothetical protein |
| ECA0193(ECAORF0188_1_sense) | 0.281652 | 2.68E-04 |  |  | argB | acetylglutamate kinase |
| ECA0197(ECAORF0192_1_sense) |  |  | 2.16009 | 2.33E-04 | ECA0197 | conserved hypothetical protein |
| ECA0199(ECAORF0194_1_sense) | 0.401574 | 0.031693 |  |  | tatA | sec-independent protein translocase |
| ECA0202(ECAORF0197_1_sense) | 1.638662 | 0.004675 |  |  | tatD | deoxyribonuclease |
| ECA0205(ECAORF0200_1_sense) | 1.580396 | 0.003116 | 1.637078 | 0.006247 | ubiD | 3-octaprenyl-4-hydroxybenzoate carboxy-lyase |
| ECA0214(ECAORF0209_1_sense) |  |  | 0.546557 | 0.004948 | birA | bira bifunctional protein [includes: biotin operon repressor; biotin--[acetyl-CoA-carboxylase] synthetase] |
| ECA0216(ECAORF0211_1_sense) |  |  | 0.377258 | 0.00136 | tufA | elongation factor Tu |
| ECA0217(ECAORF0212_1_sense) | 1.818938 | 0.0027 |  |  | secE | preprotein translocase SecE subunit |
| ECA0220(ECAORF0215_1_sense) | 3.596065 | 0.036005 |  |  | rplA | 50S ribosomal protein L1 |
| ECA0223(ECAORF0218_1_sense) | 4.199613 | 0.020897 |  |  | rpoB | DNA-directed RNA polymerase, beta-subunit |
| ECA0224(ECAORF0219_1_sense) | 2.578105 | 0.006695 |  |  | rpoC | DNA-directed RNA polymerase beta' subunit |
| ECA0231(ECAORF0227_1_sense) | 0.491767 | 0.019511 |  |  | thiE | thiamine-phosphate pyrophosphorylase |
| ECA0233(ECAORF0229_1_sense) | 0.398309 | 0.001453 |  |  | rsd | regulator of sigma D |
| ECA0235(ECAORF0231_1_sense) | 1.48755 | 0.049916 |  |  | hemE | uroporphyrinogen decarboxylase |
| ECA0237(ECAORF0232_1_sense) | 1.760905 | 0.002965 |  |  | ECA0237 | putative lipoprotein |
| ECA0239(ECAORF0234_1_sense) | 1.625043 | 0.035937 |  |  | ECA0239 | putative lipoprotein |
| ECA0240(ECAORF0235_1_sense) | 2.243784 | 0.004383 |  |  | purD | phosphoribosylamine--glycine ligase |
| ECA0241(ECAORF0236_1_sense) | 2.692686 | 5.29E-04 |  |  | purH | bifunctional purine biosynthesis protein PurH [includes: phosphoribosylaminoimidazolecarboxamide formyltransferase; IMP cyclohydrolase] |
| ECA0247(ECAORF0242_1_sense) | 0.338898 | 0.017467 |  |  | ECA0247 | putative cystine-binding periplasmic protein |
| ECA0248(ECAORF0243_1_sense) | 1.827971 | 0.024849 |  |  | ECA0248 | putative transcriptional regulator |
| ECA0251(ECAORF0246_1_sense) | 0.55676 | 0.012903 |  |  | ECA0251 | endonuclease/Exonuclease/phosphatase family protein |
| ECA0252(ECAORF0247_1_sense) | 0.435405 | 0.005699 |  |  | ECA0252 | conserved hypothetical protein |
| ECA0261(ECAORF0256_1_sense) | 2.074724 | 0.016006 |  |  | accB | biotin carboxyl carrier protein of acetyl-CoA carboxylase |
| ECA0263(ECAORF0258_1_sense) | 0.558575 | 0.00745 |  |  | ECA0263 | putative membrane protein |
| ECA0266(ECAORF0261_1_sense) | 1.484556 | 6.64E-04 |  |  | ECA0266 | putative signaling membrane protein |
| ECA0267(ECAORF0262_1_sense) | 1.526344 | 0.021261 |  |  | mreB | rod shape-determining protein |
| ECA0267(ECAORF0262_3_sense) | 1.56721 | 0.009567 |  |  | mreB | rod shape-determining protein |
| ECA0268(ECAORF0263_1_sense) | 2.86774 | 4.17E-04 |  |  | mreC | rod shape-determining protein |
| ECA0269(ECAORF0264_1_sense) | 2.950522 | 0.040594 |  |  | mreD | rod shape-determining protein |
| ECA0274(ECAORF0269_1_sense) | 0.634405 | 0.005277 |  |  | ECA0274 | conserved hypothetical protein |
| ECA0275(ECAORF0270_1_sense) |  |  | 1.921027 | 0.008641 | ECA0275 | hypothetical protein |
| ECA0277(ECAORF0272_1_sense) | 6.389954 | 0.010561 | 2.127959 | 0.049236 | ECA0277 | putative membrane protein |
| ECA0277(ECAORF0272_3_sense) | 3.159903 | 7.40E-04 | 1.845877 | 0.040575 | ECA0277 | putative membrane protein |
| ECA0279(ECAORF0274_1_sense) | 1.803737 | 0.022564 |  |  | ECA0279 | putative membrane protein |
| ECA0281(ECAORF0276_1_sense) | 1.482786 | 0.04414 |  |  | ECA0281 | conserved hypothetical protein |
| ECA0287(ECAORF0282_1_sense) | 0.528716 | 0.040906 |  |  | ECA0287 | putative sigma(54) modulation protein |
| ECA0294(ECAORF0289_1_sense) |  |  | 1.473331 | 0.015345 | ECA0294 | putative sodium/calcium exchanger protein |
| ECA0297(ECAORF0292_1_sense) |  |  | 1.895219 | 0.002963 | vpsC | putative exported protein |
| ECA0303(ECAORF0298_1_sense) | 0.469109 | 0.035451 |  |  | degQ | exported protease |
| ECA0304(ECAORF0299_3_sense) | 0.437203 | 0.002416 |  |  | ECA0304 | putative membrane protein |
| ECA0306(ECAORF0301_1_sense) | 1.918578 | 0.005656 |  |  | rplM | 50S ribosomal subunit protein L13 |
| ECA0309(ECAORF0304_1_sense) | 1.886358 | 0.004128 | 1.861819 | 3.31E-05 | sspB | stringent starvation protein B |
| ECA0310(ECAORF0305_1_sense) | 0.087925 | 0.017912 |  |  | budC | acetoin reductase |
| ECA0312(ECAORF0307_1_sense) | 1.641309 | 0.042174 |  |  | gltB | glutamate synthase [NADPH] large chain |
| ECA0323(ECAORF0318_1_sense) | 1.686302 | 0.017133 |  |  | ECA0323 | putative tetrapyrrole methylase |
| ECA0325(ECAORF0319_1_sense) | 1.994847 | 0.004279 |  |  | ECA0325 | putative plasmid stability protein |
| ECA0327(ECAORF0321_1_sense) |  |  | 2.353536 | 0.011903 | ECA0327 | conserved hypothetical protein |
| ECA0334(ECAORF0328_1_sense) | 2.062073 | 0.024418 |  |  | ECA0334 | conserved hypothetical protein |
| ECA0334(ECAORF0328_3_sense) | 2.254645 | 2.93E-04 |  |  | ECA0334 | conserved hypothetical protein |
| ECA0335(ECAORF0329_1_sense) | 2.190991 | 0.026625 |  |  | parE | topoisomerase IV subunit B |
| ECA0342(ECAORF0336_1_sense) | 2.324837 | 0.00842 |  |  | ECA0342 | PTS system, EIIa component |
| ECA0346(ECAORF0340_1_sense) | 1.785707 | 0.041858 |  |  | plsC | 1-acyl-sn-glycerol-3-phosphate acyltransferase |
| ECA0352(ECAORF0346_1_sense) |  |  | 2.982541 | 0.044647 | ECA0352 | AraC-family trancriptional regulator |
| ECA0362(ECAORF0356_1_sense) | 0.482993 | 0.004089 |  |  | scrA | PTS system, sucrose-specific IIbc component |
| ECA0362(ECAORF0356_3_sense) | 0.619548 | 0.019605 |  |  | scrA | PTS system, sucrose-specific IIbc component |
| ECA0366(ECAORF0360_1_sense) | 0.577614 | 7.31E-04 |  |  | ECA0366 | conserved hypothetical protein |
| ECA0374(ECAORF0368_1_sense) | 1.978661 | 0.01515 |  |  | nrdG | anaerobic ribonucleoside-triphosphate reductase activating protein |
| ECA0381(ECAORF0375_1_sense) | 0.492001 | 0.007381 |  |  | pyrI | aspartate carbamoyltransferase regulatory subunit |
| ECA0381(ECAORF0375_3_sense) | 0.466182 | 0.038896 |  |  | pyrI | aspartate carbamoyltransferase regulatory subunit |
| ECA0383(ECAORF0377_1_sense) | 0.289885 | 0.010823 | 1.704666 | 0.004587 | ECA0383 | conserved hypothetical protein |
| ECA0384(ECAORF0378_3_sense) | 0.434554 | 0.006721 |  |  | argI | ornithine carbamoyltransferase chain I |
| ECA0389(ECAORF0383_1_sense) | 2.876436 | 0.036008 |  |  | ECA0389 | putative hydrolase |
| ECA0403(ECAORF0396_1_sense) | 1.918851 | 0.028244 |  |  | holC | DNA polymerase III, chi subunit |
| ECA0407(ECAORF0400_1_sense) | 0.674328 | 0.017223 |  |  | ECA0407 | putative integrase (partial) |
| ECA0413(ECAORF0403_1_sense) | 0.320537 | 0.022121 |  |  | ECA0413 | hypothetical protein |
| ECA0427(ECAORF0412_1_sense) |  |  | 3.722796 | 0.007764 | ECA0427 | LysR-family transcriptional regulator |
| ECA0427(ECAORF0412_3_sense) |  |  | 4.112557 | 0.018649 | ECA0427 | LysR-family transcriptional regulator |
| ECA0435(ECAORF0420_1_sense) | 2.669737 | 0.018902 |  |  | mgtB | Magnesium transport ATPase |
| ECA0436(ECAORF0421_1_sense) | 0.49503 | 0.022028 |  |  | ECA0436 | methyl-accepting chemotaxis protein |
| ECA0442(ECAORF0427_1_sense) |  |  | 1.653056 | 0.025604 | rhaR | L-rhamnose operon transcriptional activator |
| ECA0450(ECAORF0435_1_sense) | 1.847376 | 0.03927 |  |  | rhaT | L-rhamnose-proton symport |
| ECA0451(ECAORF0436_1_sense) | 1.708208 | 0.021228 |  |  | ECA0451 | putative membrane protein |
| ECA0454(ECAORF0439_1_sense) |  |  | 0.356374 | 0.006713 | ECA0454 | putative membrane protein |
| ECA0455(ECAORF0440_1_sense) | 0.237524 | 0.011536 |  |  | ECA0455 | conserved hypothetical protein |
| ECA0456(ECAORF0441_1_sense) | 0.070738 | 0.001817 | 0.167709 | 0.016617 | *hcp4* | HcpA homologue |
| ECA0459(ECAORF0444_1_sense) |  |  | 1.570182 | 0.026715 | pepP | proline aminopeptidase II |
| ECA0459(ECAORF0444_3_sense) |  |  | 1.559727 | 0.021998 | pepP | proline aminopeptidase II |
| ECA0464(ECAORF0449_1_sense) | 1.717438 | 0.016415 |  |  | radA | putative DNA repair protein |
| ECA0473(ECAORF0458_1_sense) | 1.642724 | 0.007688 |  |  | ECA0473 | putative Na+ dependent nucleoside transporter-family protein |
| ECA0474(ECAORF0459_1_sense) |  |  | 1.626897 | 7.70E-04 | entD | enterobactin synthetase component D (4'-phosphopantetheinyl transferase) |
| ECA0477(ECAORF0462_1_sense) | 0.655838 | 0.016644 |  |  | entC | enterobactin synthetase component C (isochorismate synthase) |
| ECA0487(ECAORF0472_1_sense) | 0.043735 | 0.010292 |  |  | fom1 | phosphoenolpyruvate phosphomutase |
| ECA0488(ECAORF0473_1_sense) | 0.237432 | 0.022463 |  |  | fom2 | phosphonopyruvate decarboxylase |
| ECA0489(ECAORF0474_1_sense) |  |  | 0.501027 | 0.018869 | ECA0489 | putative 2-hydroxy-3-oxopropionate reductase |
| ECA0496(ECAORF0481_1_sense) | 0.621469 | 0.027748 |  |  | phnM | putative phosphonate metabolism protein |
| ECA0500(ECAORF0485_1_sense) | 0.271567 | 0.007479 | 0.18261 | 0.004114 | ECA0500 | putative capsular polysaccharide biosynthesis protein |
| ECA0506(ECAORF0491_1_sense) |  |  | 0.44898 | 0.030178 | ECA0506 | probable capsular polysaccharide bisynthesis glycosyl transferase |
| ECA0507(ECAORF0492_1_sense) | 0.212333 | 9.08E-06 |  |  | ECA0507 | putative capsular polysaccharide bisynthesis glycosyl transferase |
| ECA0508(ECAORF0493_1_sense) | 0.571893 | 0.006589 |  |  | ECA0508 | putative membrane protein |
| ECA0512(ECAORF0497_1_sense) | 0.571246 | 0.005202 |  |  | ECA0512 | conserved hypothetical protein |
| ECA0515(ECAORF0500_1_sense) | 0.610196 | 0.049212 |  |  | ECA0515 | conserved hypothetical protein |
| ECA0528(ECAORF0514_1_sense) | 0.41172 | 0.037078 |  |  | ECA0528 | conserved hypothetical protein |
| ECA0546(ECAORF0528_3_sense) | 0.693969 | 0.012921 |  |  | rci | shufflon-specific DNA recombinase |
| ECA0553(ECAORF0535_1_sense) |  |  | 1.746712 | 0.02223 | ECA0553 | putative exported protein |
| ECA0555(ECAORF0537_1_sense) |  |  | 2.413426 | 0.039792 | ECA0555 | putative exported protein |
| ECA0562(ECAORF0544_1_sense) | 0.424776 | 0.011172 |  |  | ECA0562 | hypothetical protein |
| ECA0563(ECAORF0545_1_sense) | 0.275616 | 7.55E-04 | 0.31269 | 0.022119 | ECA0563 | putative membrane protein |
| ECA0575(ECAORF0557_1_sense) | 0.610292 | 0.02852 |  |  | ECA0575 | putative membrane protein |
| ECA0586A(ECAORF0569_1_sense) | 0.491345 | 0.016873 | 0.493287 | 0.012552 | ECA0586A | hypothetical protein |
| ECA0601(ECAORF0584_1_sense) |  |  | 0.569455 | 0.018411 | ECA0601 | putative oxidoreductase |
| ECA0606(ECAORF0589_1_sense) | 0.602993 | 0.012786 |  |  | cfa3 | Cfa-beta-ketoacylsynthase |
| ECA0607(ECAORF0590_1_sense) | 1.683671 | 0.007196 | 0.505289 | 0.02377 | cfa2 | coronafacic acid dehydratase |
| ECA0613(ECAORF0596_1_sense) | 0.661058 | 0.041378 |  |  | ECA0613 | conserved hypothetical protein |
| ECA0621(ECAORF0604_1_sense) | 0.299544 | 0.026451 |  |  | aspA2 | aspartate ammonia-lyase 2 |
| ECA0623(ECAORF0605_1_sense) |  |  | 2.679145 | 0.018742 | fxsA | suppressor of F plamsid exlusion of phage T7 |
| ECA0627(ECAORF0609_1_sense) |  |  | 1.982998 | 0.00113 | ubiA | 4-hydroxybenzoate octaprenyl transferase |
| ECA0634(ECAORF0616_1_sense) |  |  | 2.344868 | 0.011902 | ECA0634 | LysR-family transcriptional regulator |
| ECA0636(ECAORF0618_1_sense) | 12.16633 | 0.031651 |  |  | ECA0636 | putative membrane protein |
| ECA0642(ECAORF0624_3_sense) | 1.587262 | 0.021348 |  |  | ECA0642 | putative DedA-family membrane protein |
| ECA0658(ECAORF0639_1_sense) | 0.584945 | 0.025463 |  |  | ECA0658 | putative exported protein |
| ECA0660(ECAORF0641_1_sense) | 0.224637 | 0.005176 |  |  | ECA0660 | putative exported protein |
| ECA0664(ECAORF0645_1_sense) |  |  | 0.712398 | 0.002343 | ECA0664 | hypothetical protein |
| ECA0676(ECAORF0657_1_sense) | 2.107541 | 0.040489 |  |  | ECA0676 | hypothetical protein |
| ECA0677(ECAORF0658_1_sense) | 3.451571 | 0.015147 |  |  | ECA0677 | putative phage regulatory protein |
| ECA0678(ECAORF0659_1_sense) | 5.439172 | 0.015188 |  |  | ECA0678 | putative phage regulatory protein |
| ECA0681(ECAORF0662_1_sense) | 4.224945 | 0.002516 |  |  | dnaG | DNA primase |
| ECA0682(ECAORF0663_1_sense) | 2.664927 | 0.012562 |  |  | rpsU | 30S ribosomal subunit protein S21 |
| ECA0692(ECAORF0673_1_sense) | 6.553849 | 3.51E-04 |  |  | ECA0692 | putative membrane protein |
| ECA0693(ECAORF0674_1_sense) | 4.175415 | 0.020214 | 0.628824 | 0.006977 | ECA0693 | putative GTP-binding protein |
| ECA0701(ECAORF0682_1_sense) | 1.707308 | 0.001845 |  |  | ECA0701 | hypothetical protein |
| ECA0702(ECAORF0683_1_sense) | 2.867754 | 0.002224 |  |  | secG | protein-export membrane protein |
| ECA0709(ECAORF0689_1_sense) | 5.552638 | 5.20E-04 |  |  | ECA0709 | hypothetical protein |
| ECA0709(ECAORF0689_3_sense) | 4.975186 | 0.002203 |  |  | ECA0709 | hypothetical protein |
| ECA0710(ECAORF0690_1_sense) | 6.548424 | 0.008563 |  |  | ECA0710 | conserved hypothetical protein |
| ECA0711(ECAORF0691_1_sense) | 3.746394 | 0.005316 |  |  | nusA | N utilization substance protein A |
| ECA0712(ECAORF0692_1_sense) | 4.202655 | 0.018945 |  |  | infB | translation initiation factor IF-2 |
| ECA0714(ECAORF0694_1_sense) | 4.888781 | 0.015217 |  |  | truB | tRNA pseudouridine synthase B |
| ECA0716(ECAORF0696_1_sense) | 2.388637 | 0.021195 |  |  | pnp | polyribonucleotide nucleotidyltransferase |
| ECA0717(ECAORF0697_1_sense) | 8.146603 | 5.54E-04 |  |  | nlpI | lipoprotein |
| ECA0718(ECAORF0698_1_sense) | 15.71555 | 6.33E-04 |  |  | deaD | ATP-independent RNA helicase |
| ECA0723(ECAORF0701_1_sense) | 0.34657 | 0.032941 |  |  | ECA0723 | putative sterol transferase |
| ECA0724(ECAORF0702_1_sense) | 0.620031 | 4.77E-04 | 2.019346 | 0.026049 | ECA0724 | putative acetyltransferase |
| ECA0726(ECAORF0704_3_sense) | 1.866536 | 0.005098 |  |  | ECA0726 | hypothetical protein |
| ECA0727(ECAORF0705_1_sense) | 0.629276 | 0.013258 |  |  | deoC | deoxyribose-phosphate aldolase |
| ECA0728(ECAORF0706_1_sense) |  |  | 0.62442 | 0.049935 | deoA | thymidine phosphorylase |
| ECA0732(ECAORF0710_1_sense) | 1.501778 | 0.001942 |  |  | ECA0732 | putative exported protein |
| ECA0733(ECAORF0711_1_sense) | 2.468595 | 0.034162 |  |  | rimI | ribosomal-protein-alanine acetyltransferase |
| ECA0734(ECAORF0712_1_sense) |  |  | 2.292251 | 0.028342 | holD | DNA polymerase III, psi subunit |
| ECA0735(ECAORF0713_1_sense) | 1.935212 | 7.59E-04 |  |  | rsmC | ribosomal RNA small subunit methyltransferase C |
| ECA0738(ECAORF0716_3_sense) | 1.612606 | 0.012955 |  |  | ECA0738 | putative membrane protein |
| ECA0740(ECAORF0718_1_sense) | 0.201492 | 0.004417 | 0.336608 | 0.032444 | budA | alpha-acetolactate decarboxylase |
| ECA0744(ECAORF0722_1_sense) | 0.255224 | 0.004676 | 0.604982 | 0.009949 | gcvH | glycine cleavage system H protein |
| ECA0754(ECAORF0732_1_sense) |  |  | 1.979718 | 0.02861 | rafA | alpha-galactosidase |
| ECA0759(ECAORF0737_1_sense) |  |  | 2.461667 | 0.020907 | ECA0759 | conserved hypothetical protein |
| ECA0760(ECAORF0738_1_sense) | 1.625613 | 0.044909 |  |  | ECA0760 | putative membrane protein |
| ECA0761(ECAORF0739_1_sense) | 1.627122 | 0.001102 |  |  | ECA0761 | conserved hypothetical protein |
| ECA0772(ECAORF0750_1_sense) | 1.611078 | 0.034637 |  |  | recJ | single-stranded DNA-specific exonuclease |
| ECA0774(ECAORF0752_1_sense) | 2.322358 | 0.008281 |  |  | lysS | lysyl tRNA synthetase |
| ECA0778(ECAORF0756_1_sense) |  |  | 0.482262 | 0.041109 | ECA0778 | putative membrane protein |
| ECA0779(ECAORF0757_1_sense) | 2.835361 | 0.024063 |  |  | ECA0779 | conserved hypothetical protein |
| ECA0781(ECAORF0759_1_sense) | 1.995116 | 0.002042 |  |  | ddg | putative lipid A biosynthesis acyltransferase |
| ECA0783(ECAORF0761_1_sense) | 2.321419 | 0.001362 | 1.738145 | 0.009936 | ECA0783 | putative permease |
| ECA0800(ECAORF0778_1_sense) | 0.551048 | 0.026525 | 0.44671 | 1.93E-04 | ECA0800 | conserved hypothetical protein |
| ECA0809(ECAORF0786_1_sense) | 0.417384 | 0.041709 |  |  | hexY | global regulatory protein |
| ECA0809(ECAORF0786_3_sense) | 0.410586 | 0.035439 |  |  | hexY | global regulatory protein |
| ECA0813(ECAORF0789_1_sense) | 3.791384 | 0.028773 |  |  | ECA0813 | RNA polymerase sigma factor |
| ECA0814(ECAORF0790_1_sense) |  |  | 0.49815 | 0.040263 | ECA0814 | putative iron sensor protein |
| ECA0815(ECAORF0791_1_sense) |  |  | 2.244577 | 0.026668 | ECA0815 | putative membrane protein |
| ECA0818(ECAORF0794_1_sense) | 0.634882 | 0.009482 |  |  | ECA0818 | putative membrane protein |
| ECA0820(ECAORF0796_1_sense) | 0.160182 | 0.036695 |  |  | ECA0820 | O-acetyl-L-homoserine sulfhydrylase |
| ECA0821(ECAORF0797_1_sense) | 1.830337 | 0.003958 |  |  | ECA0821 | putative exported protein |
| ECA0823(ECAORF0799_1_sense) | 0.55265 | 8.60E-05 |  |  | cybB | cytochrome B561 |
| ECA0830(ECAORF0806_1_sense) | 0.370926 | 0.008533 |  |  | ECA0830 | putative exported protein |
| ECA0833(ECAORF0809_1_sense) |  |  | 0.548804 | 0.011909 | ECA0833 | phage regulatory protein |
| ECA0846(ECAORF1492_1_sense) | 0.674149 | 0.032945 |  |  |  | PROBE02913 |
| ECA0850(ECAORF0826_1_sense) |  |  | 1.715517 | 0.015253 | ECA0850 | putative sugar ABC transporter, permease protein |
| ECA0850(ECAORF0826_3_sense) |  |  | 1.510019 | 0.012832 | ECA0850 | putative sugar ABC transporter, permease protein |
| ECA0851(ECAORF0827_1_sense) | 0.581713 | 0.03214 |  |  | ECA0851 | putative sugar ABC transporter ATP-binding protein |
| ECA0852(ECAORF0828_1_sense) |  |  | 0.477593 | 0.029567 | ECA0852 | putative exported plant proteoglycan hydrolase |
| ECA0855(ECAORF0831_1_sense) |  |  | 1.424401 | 0.03864 | ECA0855 | ABC transporter permease protein |
| ECA0875(ECAORF0850_1_sense) | 0.578898 | 5.33E-04 | 1.418143 | 0.03133 | ECA0875 | putative GNAT-family acetyltransferase |
| ECA0877(ECAORF0852_1_sense) | 0.568295 | 0.016919 |  |  | ECA0877 | TonB-like protein |
| ECA0877(ECAORF0852_3_sense) | 0.640155 | 0.048846 |  |  | ECA0877 | TonB-like protein |
| ECA0881(ECAORF0856_1_sense) | 2.297977 | 0.01177 |  |  | ECA0881 | putative exported protein |
| ECA0891(ECAORF0866_1_sense) | 0.524909 | 0.030508 |  |  | crr | PTS system, glucose-specific IIa component |
| ECA0891(ECAORF0866_2_sense) | 0.560452 | 0.016121 |  |  | crr | PTS system, glucose-specific IIa component |
| ECA0891(ECAORF0866_3_sense) | 0.61074 | 0.015176 |  |  | crr | PTS system, glucose-specific IIa component |
| ECA0893(ECAORF0868_1_sense) | 0.329757 | 0.022735 |  |  | ptsH | PTS system phosphocarrier protein |
| ECA0894(ECAORF0869_1_sense) | 0.279168 | 1.54E-04 |  |  | cysK | cysteine synthase A |
| ECA0898(ECAORF0874_1_sense) | 0.59805 | 8.61E-04 |  |  | ECA0898 | conserved hypothetical protein |
| ECA0898(ECAORF0874_2_sense) | 0.420831 | 0.002087 |  |  | ECA0898 | conserved hypothetical protein |
| ECA0898(ECAORF0874_3_sense) | 0.514998 | 0.001585 |  |  | ECA0898 | conserved hypothetical protein |
| ECA0904(ECAORF0880_1_sense) |  |  | 6.048334 | 0.041232 | fumA | fumarate hydratase class I, aerobic |
| ECA0915(ECAORF0891_1_sense) | 0.396993 | 0.014509 | 0.216078 | 0.003547 | ECA0915 | putative exported protein |
| ECA0916(ECAORF0892_1_sense) | 0.164309 | 0.001663 | 0.44474 | 0.014254 | ECA0916 | LysR-family transcriptional regulator |
| ECA0920(ECAORF0896_1_sense) | 0.515385 | 6.89E-04 |  |  | ECA0920 | probable carbohydrate oxidoreductase |
| ECA0921(ECAORF0897_1_sense) |  |  | 1.625054 | 0.029123 | ECA0921 | GntR-family transcriptional regulator |
| ECA0923(ECAORF0899_1_sense) | 0.424034 | 0.049384 |  |  | ECA0923 | conserved hypothetical protein |
| ECA0930(ECAORF0906_1_sense) | 1.812936 | 0.02144 |  |  | ECA0930 | probable permease |
| ECA0931(ECAORF0907_1_sense) | 0.1445 | 0.005153 | 0.299885 | 0.04785 | ECA0931 | putative avirulence protein |
| ECA0932(ECAORF0908_1_sense) | 0.18503 | 0.038293 |  |  | ECA0932 | permease |
| ECA0933(ECAORF0909_1_sense) | 0.103957 | 0.004199 |  |  | ECA0933 | LuxR-family transcriptional regulator |
| ECA0934(ECAORF0910_1_sense) | 0.195526 | 0.002153 | 0.345035 | 0.014277 | ECA0934 | putative acetyltransferase |
| ECA0945(ECAORF0921_1_sense) | 0.519054 | 0.007609 |  |  | ECA0945 | putative exported protein |
| ECA0952(ECAORF0928_1_sense) | 0.36745 | 0.007715 |  |  | ECA0952 | hypothetical protein |
| ECA0953(ECAORF0929_1_sense) | 0.386462 | 0.031343 |  |  | ECA0953 | hypothetical protein |
| ECA0960(ECAORF0936_1_sense) | 0.218493 | 0.005985 |  |  | ECA0960 | putative restriction enzyme |
| ECA0964(ECAORF0938_2_sense) | 0.633441 | 0.048156 |  |  | ECA0964 | putative regulatory protein |
| ECA0964(ECAORF0938_3_sense) | 0.614272 | 0.011811 |  |  | ECA0964 | putative regulatory protein |
| ECA0975(ECAORF0949_1_sense) |  |  | 1.797885 | 9.31E-04 | ECA0975 | conserved hypothetical protein |
| ECA0977(ECAORF0951_1_sense) | 2.371339 | 8.18E-05 |  |  | ECA0977 | putative methyltransferase |
| ECA0981(ECAORF0955_1_sense) | 0.510001 | 0.01661 |  |  | tas | putative aldo/keto reductase-family protein |
| ECA0983(ECAORF0957_1_sense) | 2.00275 | 0.01932 |  |  | ECA0983 | putative membrane protein |
| ECA0994(ECAORF0968_1_sense) | 1.58754 | 0.041455 |  |  | ptrA | protease III precursor |
| ECA0995(ECAORF0969_1_sense) |  |  | 1.735379 | 0.022971 | recB | exodeoxyribonuclease V beta chain |
| ECA0997(ECAORF0971_1_sense) | 0.597525 | 0.003834 |  |  | ECA0997 | putative lacI-family transcriptional regulator |
| ECA0999(ECAORF0973_1_sense) |  |  | 0.44818 | 0.008376 | argA | amino-acid acetyltransferase |
| ECA1008(ECAORF0982_1_sense) | 0.132225 | 0.026503 |  |  | ECA1008 | ABC transporter, substrate binding protein |
| ECA1010(ECAORF0984_1_sense) | 1.531822 | 0.024478 |  |  | ECA1010 | conserved hypothetical protein |
| ECA1022(ECAORF0996_1_sense) | 0.09508 | 0.006032 | 0.272401 | 0.030343 | aepA | exoenzymes regulatory protein |
| ECA1023(ECAORF0997_1_sense) |  |  | 0.547658 | 0.039893 | ECA1023 | MarR-family transcriptional regulator |
| ECA1027(ECAORF1001_1_sense) | 0.279773 | 0.003128 |  |  | ECA1027 | conserved hypothetical protein |
| ECA1030(ECAORF1004_1_sense) |  |  | 1.589265 | 0.029441 | map | methionine aminopeptidase |
| ECA1031(ECAORF1005_1_sense) | 2.710629 | 0.026034 |  |  | rpsB | 30S ribosomal protein S2 |
| ECA1033(ECAORF1007_1_sense) | 3.582984 | 0.005467 |  |  | pyrH | uridylate kinase |
| ECA1037(ECAORF1011_1_sense) | 2.060541 | 0.005872 |  |  | cdsA | phosphatidate cytidylyltransferase |
| ECA1038(ECAORF1012_1_sense) | 2.647411 | 8.56E-04 |  |  | ecfE | protease |
| ECA1039(ECAORF1013_1_sense) | 2.051659 | 0.00653 |  |  | ecfK | putative surface antigen |
| ECA1041(ECAORF1015_1_sense) | 2.450757 | 0.027298 | 1.786722 | 0.021884 | lpxD | UDP-3-O-[3-hydroxymyristoyl] glucosamine N-acyltransferase |
| ECA1044(ECAORF1018_1_sense) | 2.512894 | 0.001798 |  |  | lpxB | lipid-A-disaccharide synthase |
| ECA1045(ECAORF1019_1_sense) | 2.078307 | 0.004095 | 0.60528 | 0.015291 | rnhB | ribonuclease HII |
| ECA1046(ECAORF1020_1_sense) | 2.454676 | 0.041713 |  |  | dnaE | DNA polymerase III alpha subunit |
| ECA1049(ECAORF1023_1_sense) | 2.176504 | 0.028808 | 1.675528 | 0.020783 | mesJ | cell cycle protein |
| ECA1054(ECAORF1028_1_sense) | 0.073093 | 0.010495 | 0.418395 | 0.019961 | ECA1054 | putative integrase |
| ECA1055(ECAORF1029_1_sense) | 0.062825 | 1.52E-04 | 0.28093 | 0.001413 | ECA1055 | hypothetical protein |
| ECA1056(ECAORF1030_1_sense) | 0.136057 | 9.55E-04 |  |  | ECA1056 | putative phage-related reverse transcriptase/maturase family protein |
| ECA1057(ECAORF1031_1_sense) | 0.100356 | 5.02E-04 | 0.255692 | 0.04063 | ECA1057 | phage-related hypothetical protein |
| ECA1058(ECAORF1032_1_sense) | 0.331962 | 0.034626 |  |  | ECA1058 | hypothetical protein |
| ECA1059(ECAORF1033_1_sense) | 0.491593 | 0.003146 |  |  | ECA1059 | putative integrase |
| ECA1061(ECAORF1035_1_sense) | 0.534683 | 0.015473 |  |  | ECA1061 | putative integrase |
| ECA1076(ECAORF1050_1_sense) | 0.409509 | 0.014156 |  |  | fecB | iron(III) dicitrate-binding periplasmic protein |
| ECA1079(ECAORF1053_1_sense) | 3.198208 | 0.025118 |  |  | fecI | RNA polymerase sigma factor |
| ECA1086(ECAORF1060_1_sense) | 2.054688 | 0.031289 |  |  | ECA1086 | putative signaling protein |
| ECA1101(ECAORF1074_1_sense) | 1.706478 | 0.039398 |  |  | ECA1101 | putative ABC transporter ATP-binding protein |
| ECA1105(ECAORF1078_1_sense) | 0.520876 | 0.022109 |  |  | ECA1105 | putative methyl-accepting chemotaxis protein |
| ECA1110(ECAORF1083_1_sense) |  |  | 1.526334 | 0.030494 | phoB | phosphate regulon response regulator |
| ECA1114(ECAORF1087_1_sense) | 1.767955 | 0.023418 |  |  | brnQ | branched-chain amino acid transport system II carrier protein |
| ECA1115(ECAORF1088_1_sense) | 2.257497 | 0.017773 |  |  | proY | proline-specific permease |
| ECA1117(ECAORF1090_1_sense) | 0.496669 | 0.001286 |  |  | ECA1117 | probable peroxidase |
| ECA1119(ECAORF1092_1_sense) | 2.405299 | 7.76E-04 |  |  | queA | S-adenosylmethionine:tRNA ribosyltransferase-isomerase |
| ECA1119(ECAORF1092_3_sense) | 2.469518 | 0.015116 |  |  | queA | S-adenosylmethionine:tRNA ribosyltransferase-isomerase |
| ECA1120(ECAORF1093_1_sense) | 2.259869 | 0.015748 |  |  | tgt | queuine tRNA-ribosyltransferase |
| ECA1121(ECAORF1094_1_sense) | 1.54326 | 0.022058 |  |  | ECA1121 | putative membrane subunit of preprotein translocase |
| ECA1122(ECAORF1095_1_sense) | 2.172182 | 3.27E-04 |  |  | secD | protein-export membrane protei |
| ECA1123(ECAORF1096_1_sense) | 1.56818 | 0.005767 |  |  | secF | protein-export membrane protein |
| ECA1127(ECAORF1100_1_sense) | 2.247933 | 0.033463 |  |  | ribH | 6,7-dimethyl-8-ribityllumazine synthase |
| ECA1128(ECAORF1101_1_sense) | 1.843921 | 0.009228 |  |  | nusB | N utilization substance protein B |
| ECA1129(ECAORF1102_1_sense) | 2.286056 | 0.003647 |  |  | thiL | thiamine-monophosphate kinase |
| ECA1130(ECAORF1103_1_sense) | 2.318112 | 8.99E-05 |  |  | pgpA | phosphatidylglycerophosphatase A |
| ECA1133(ECAORF1106_1_sense) | 1.598115 | 0.005109 |  |  | xseB | exodeoxyribonuclease VII small subunit |
| ECA1133(ECAORF1106_3_sense) | 2.465738 | 0.001202 |  |  | xseB | exodeoxyribonuclease VII small subunit |
| ECA1134(ECAORF1107_1_sense) | 3.420708 | 0.014508 |  |  | thiI | thiamine biosynthesis protein |
| ECA1138(ECAORF1111_1_sense) | 1.820181 | 0.0031 |  |  | ECA1138 | probable transporter |
| ECA1143(ECAORF1116_1_sense) | 1.696948 | 0.019031 |  |  | cyoA | cytochrome o ubiquinol oxidase subunit II |
| ECA1147(ECAORF1120_1_sense) | 1.796628 | 0.037897 |  |  | tig | trigger factor |
| ECA1152(ECAORF1125_1_sense) | 2.144431 | 0.024244 |  |  | ppiD | peptidyl-prolyl cis-trans isomerase D |
| ECA1156(ECAORF1129_1_sense) | 4.36607 | 0.008161 |  |  | ECA1156 | putative pyridoxal-phosphate dependent protein |
| ECA1158(ECAORF1131_1_sense) | 2.555953 | 0.011194 |  |  | mdlA | ATP-binding component of ABC transporter |
| ECA1165(ECAORF1138_1_sense) | 2.936165 | 0.04734 |  |  | hha | haemolysin expression modulating protein |
| ECA1183(ECAORF1156_1_sense) | 0.37598 | 0.034497 | 0.634052 | 0.041623 | ECA1183 | hypothetical protein |
| ECA1186(ECAORF1159_1_sense) |  |  | 0.312936 | 0.025398 | ECA1186 | hypothetical protein |
| ECA1191(ECAORF1164_1_sense) |  |  | 2.491224 | 0.021325 | ECA1191 | conserved hypothetical protein |
| ECA1197(ECAORF1170_1_sense) | 1.86944 | 0.013241 |  |  | ECA1197 | putative ferredoxin |
| ECA1198(ECAORF1171_1_sense) | 3.046902 | 0.017263 |  |  | nrdB | ribonucleoside-diphosphate reductase 1 beta chain |
| ECA1199(ECAORF1172_1_sense) | 2.029069 | 0.018675 |  |  | nrdA | ribonucleoside-diphosphate reductase 1 alpha chain |
| ECA1201(ECAORF1174_1_sense) | 1.894072 | 0.004538 |  |  | gyrA | DNA gyrase subunit A |
| ECA1213(ECAORF1186_1_sense) | 1.87668 | 0.001755 | 0.551517 | 0.016279 | menB | naphthoate synthase |
| ECA1215(ECAORF1188_1_sense) | 1.885993 | 0.012032 |  |  | menE | O-succinylbenzoic acid--CoA ligase |
| ECA1225(ECAORF1198_1_sense) | 0.228042 | 0.034386 | 1.770607 | 0.045293 | hybO | hydrogenase-2 small subunit |
| ECA1226(ECAORF1199_1_sense) | 0.295154 | 0.025032 |  |  | hybA | hydrogenase-2 operon protein |
| ECA1235(ECAORF1208_1_sense) | 0.321523 | 0.006621 |  |  | hypA | putative regulator of hydrogenase isoenzymes |
| ECA1236(ECAORF1209_1_sense) |  |  | 1.904701 | 0.045187 | hyfR | hydrogenase-4 transcriptional activator |
| ECA1237(ECAORF1210_1_sense) | 0.567208 | 0.031159 |  |  | hycI | hydrogenase 3 maturation protease |
| ECA1249(ECAORF4431_1_sense) | 0.236007 | 0.001791 | 0.463541 | 0.046636 |  | PROBE08526 |
| ECA1250(ECAORF1477_1_sense) | 0.309628 | 0.035513 | 0.62431 | 0.033571 |  | PROBE02884 |
| ECA1253(ECAORF1226_1_sense) | 2.002065 | 0.002708 |  |  | purN | phosphoribosylglycinamide formyltransferase |
| ECA1254(ECAORF1227_1_sense) | 3.284561 | 3.45E-06 |  |  | purM | phosphoribosylformylglycinamidine cyclo-ligase |
| ECA1256(ECAORF1229_1_sense) | 2.956798 | 5.72E-05 |  |  | uraA | uracil permease |
| ECA1257(ECAORF1230_1_sense) | 2.195542 | 0.017017 |  |  | ECA1257 | conserved hypothetical protein |
| ECA1261(ECAORF1234_1_sense) | 0.449717 | 0.047826 |  |  | gcvR | glycine cleavage system transcriptional repressor |
| ECA1274(ECAORF1247_1_sense) | 0.125883 | 0.048177 |  |  | ECA1274 | probable RNA polymerase sigma factor |
| ECA1280(ECAORF1253_1_sense) | 3.029155 | 0.029997 |  |  | ECA1280 | putative phospholipase/Carboxylesterase family protein |
| ECA1281(ECAORF1254_1_sense) | 2.038651 | 0.001767 |  |  | ECA1281 | methyl-accepting chemotaxis protein |
| ECA1281(ECAORF1254_3_sense) | 2.145432 | 0.005486 |  |  | ECA1281 | methyl-accepting chemotaxis protein |
| ECA1294(ECAORF1267_1_sense) | 6.132953 | 0.031762 |  |  | cspE | cold shock-like protein |
| ECA1300(ECAORF1273_1_sense) | 1.645538 | 0.022983 |  |  | dacA | D-alanine carboxypeptidase |
| ECA1301(ECAORF1274_1_sense) | 1.525686 | 0.043501 |  |  | rlpA | rare lipoprotein A |
| ECA1303(ECAORF1276_1_sense) | 4.091085 | 0.023068 |  |  | mrdA | penicillin-binding protein 2 |
| ECA1304(ECAORF1277_1_sense) | 3.517039 | 1.29E-04 |  |  | ECA1304 | conserved hypothetical protein |
| ECA1307(ECAORF1280_1_sense) | 1.671325 | 0.032599 |  |  | holA | DNA polymerase III, delta subunit |
| ECA1309(ECAORF1282_1_sense) | 1.820574 | 0.002491 |  |  | leuS | leucyl-tRNA synthetase |
| ECA1315(ECAORF1288_1_sense) | 1.91672 | 9.66E-04 |  |  | lnt | apolipoprotein N-acyltransferase |
| ECA1320(ECAORF1293_3_sense) |  |  | 1.565338 | 0.037088 | ubiF | 2-octaprenyl-3-methyl-6-methoxy-1,4-benzoquinol hydroxylase |
| ECA1321(ECAORF1294_1_sense) |  |  | 1.755288 | 0.007415 | ECA1321 | putative membrane protein |
| ECA1325(ECAORF1298_1_sense) |  |  | 0.637967 | 2.58E-04 | nagA | N-acetylglucosamine-6-phosphate deacetylase |
| ECA1326(ECAORF1299_1_sense) |  |  | 0.524037 | 0.022669 | nagB | glucosamine-6-phosphate isomerase |
| ECA1328(ECAORF1301_1_sense) | 1.96729 | 0.00179 |  |  | glnS | glutaminyl-tRNA synthetase |
| ECA1332(ECAORF1305_1_sense) |  |  | 0.502272 | 8.01E-04 | ECA1332 | methyl-accepting chemotaxis protein |
| ECA1333(ECAORF1306_1_sense) | 3.824247 | 6.41E-04 |  |  | ECA1333 | conserved hypothetical protein |
| ECA1333(ECAORF1306_3_sense) | 3.095491 | 0.029827 |  |  | ECA1333 | conserved hypothetical protein |
| ECA1336(ECAORF1309_1_sense) |  |  | 1.744276 | 0.003107 | pgm | phosphoglucomutase |
| ECA1344(ECAORF1317_1_sense) | 0.625114 | 0.035243 |  |  | ECA1344 | putative membrane protein |
| ECA1348(ECAORF1321_1_sense) |  |  | 1.519476 | 0.003456 | ECA1348 | conserved hypothetical protein |
| ECA1351(ECAORF1324_1_sense) | 2.034795 | 0.040797 |  |  | ECA1351 | putative allophanate hydrolase subunit 1 |
| ECA1356(ECAORF1329_1_sense) | 0.511154 | 0.014823 |  |  | gltA | citrate synthase |
| ECA1361(ECAORF1334_3_sense) | 0.555174 | 0.014393 |  |  | sucA | 2-oxoglutarate dehydrogenase E1 component |
| ECA1362(ECAORF1335_1_sense) | 0.440509 | 0.001877 |  |  | sucB | dihydrolipoamide succinyltransferase component of 2-oxoglutarate dehydrogenase complex |
| ECA1364(ECAORF1337_1_sense) | 0.513709 | 0.020879 |  |  | sucD | succinyl-CoA synthetase alpha chain |
| ECA1368(ECAORF1341_1_sense) |  |  | 1.584258 | 0.036967 | ECA1368 | putative membrane protein |
| ECA1368(ECAORF1341_2_sense) |  |  | 1.983047 | 0.001624 | ECA1368 | putative membrane protein |
| ECA1368(ECAORF1341_3_sense) |  |  | 1.589494 | 0.028409 | ECA1368 | putative membrane protein |
| ECA1369(ECAORF1342_1_sense) | 1.854422 | 4.63E-04 |  |  | ECA1369 | putative thioesterase |
| ECA1370(ECAORF1343_1_sense) | 1.786519 | 0.03778 |  |  | tolQ | TolQ protein |
| ECA1371(ECAORF1344_1_sense) | 1.635451 | 0.009666 |  |  | tolR | tolR protein |
| ECA1372(ECAORF1345_1_sense) | 2.0553 | 0.041061 |  |  | tolA | TolA protein |
| ECA1374(ECAORF1347_1_sense) |  |  | 2.390462 | 0.049853 | pal | peptidoglycan-associated lipoprotein precursor |
| ECA1381(ECAORF1354_1_sense) | 1.558355 | 0.035115 |  |  | aroG | phospho-2-dehydro-3-deoxyheptonate aldolase, Phe-sensitive |
| ECA1396(ECAORF1369_1_sense) | 0.440493 | 0.002911 |  |  | ECA1396 | putative exported protein |
| ECA1406(ECAORF1379_1_sense) |  |  | 0.68617 | 0.038619 | fdnI | formate dehydrogenase, nitrate-inducible, cytochrome b556 |
| ECA1406(ECAORF1379_3_sense) |  |  | 0.577031 | 0.018544 | fdnI | formate dehydrogenase, nitrate-inducible, cytochrome b556 |
| ECA1409(ECAORF1382_1_sense) | 2.20375 | 0.00392 |  |  | metG | methionyl-tRNA synthetase |
| ECA1410(ECAORF1383_1_sense) |  |  | 1.703939 | 0.001779 | ECA1410 | conserved hypothetical protein |
| ECA1411(ECAORF1384_1_sense) | 2.101248 | 0.012843 |  |  | udk | uridine kinase |
| ECA1412(ECAORF1385_1_sense) | 1.849585 | 0.024797 |  |  | dcd | deoxycytidine triphosphate deaminase |
| ECA1417(ECAORF1390_1_sense) | 1.390625 | 0.039692 |  |  | wza | putative polysaccharide export protein |
| ECA1424(ECAORF1397_1_sense) | 1.764398 | 0.00388 |  |  | rfbH | CDP-4-keto-6-deoxy-D-glucose-3-dehydratase |
| ECA1452(ECAORF1425_3_sense) |  |  | 1.690201 | 0.020766 | ECA1452 | putative beta-glucoside operon antiterminator |
| ECA1466(ECAORF1439_1_sense) | 1.80473 | 1.78E-04 |  |  | ECA1466 | conserved hypothetical protein |
| ECA1468(ECAORF1441_1_sense) | 2.266863 | 0.004162 |  |  | ECA1468 | ABC transporter, ATP-binding protein |
| ECA1472(ECAORF1445_1_sense) | 0.472099 | 0.00617 |  |  | ECA1472 | conserved hypothetical protein |
| ECA1474(ECAORF1446_1_sense) | 0.387568 | 0.040227 |  |  | ECA1474 | conserved hypothetical protein |
| ECA1475(ECAORF1447_1_sense) | 0.471339 | 0.005308 | 0.183855 | 0.005172 | ECA1475 | conserved hypothetical protein |
| ECA1498(ECAORF1470_1_sense) | 0.233733 | 0.008325 |  |  | nlpA | lipoprotein-28 precursor |
| ECA1499(ECAORF1471_1_sense) | 0.18354 | 0.004401 |  |  | pnl | pectin lyase |
| ECA1509(ECAORF1478_1_sense) | 0.23608 | 0.012957 |  |  | ECA1509 | methyl-accepting chemotaxis protein |
| ECA1510(ECAORF1479_1_sense) | 4.282314 | 0.011771 |  |  | ECA1510 | putative iron permease |
| ECA1511(ECAORF1480_1_sense) | 0.301216 | 0.041781 |  |  | ECA1511 | putative exported protein |
| ECA1515(ECAORF1484_1_sense) |  |  | 1.803099 | 0.003063 | ECA1515 | ABC transporter ATP binding protein |
| ECA1518(ECAORF1487_1_sense) | 4.046663 | 0.001487 |  |  | ECA1518 | AraC-family transcriptional regulator |
| ECA1520(ECAORF1474_1_sense) | 2.114089 | 0.007259 |  |  | gltP | proton glutamate symport protein |
| ECA1520(ECAORF1489_1_sense) |  |  | 0.547594 | 0.036009 |  | PROBE02908 |
| ECA1521(ECAORF1490_1_sense) | 0.374122 | 0.015973 |  |  | ECA1521 | hypothetical protein |
| ECA1525(ECAORF1494_1_sense) | 2.903052 | 0.002566 |  |  | ECA1525 | ABC transporter ATP-binding protein |
| ECA1527(ECAORF1496_1_sense) |  |  | 1.525076 | 0.01839 | ECA1527 | putative malate/lactate dehydrogenase |
| ECA1537(ECAORF1506_1_sense) | 0.543793 | 0.026032 |  |  | hasA | extracellular heme-binding protein |
| ECA1537(ECAORF1506_3_sense) | 0.55146 | 0.042239 |  |  | hasA | extracellular heme-binding protein |
| ECA1540(ECAORF1509_1_sense) | 2.759874 | 2.94E-04 |  |  | ECA1540 | ECF sigma factor |
| ECA1544(ECAORF1513_1_sense) | 1.664032 | 0.040747 |  |  | ECA1544 | putative methyltransferase |
| ECA1547(ECAORF1516_1_sense) | 1.519704 | 0.031332 |  |  | amn | AMP nucleosidase |
| ECA1549(ECAORF1518_1_sense) | 2.092057 | 0.041611 |  |  | ECA1549 | putative ABC transporter substrate-binding protein |
| ECA1561(ECAORF1530_1_sense) | 0.617562 | 0.004395 |  |  | ECA1561 | quorum-sensing transcriptional regulator |
| ECA1562(ECAORF1531_1_sense) | 3.697231 | 0.00648 | 1.821029 | 0.002092 | ECA1562 | TetR-family transcriptional regulator |
| ECA1563(ECAORF1532_1_sense) |  |  | 1.571091 | 0.011901 | ECA1563 | putative lipoprotein |
| ECA1568(ECAORF1537_1_sense) | 0.360317 | 0.001647 |  |  | ECA1568 | putative chemotaxis signal transduction protein |
| ECA1575(ECAORF1543_1_sense) |  |  | 2.499721 | 0.024361 | ECA1575 | MerR-family transcriptional regulator |
| ECA1577(ECAORF1545_1_sense) | 0.547943 | 0.010259 |  |  | ECA1577 | putative lipoprotein |
| ECA1580(ECAORF1548_1_sense) |  |  | 0.638625 | 0.01967 | ECA1580 | putative cold-shock protein |
| ECA1583(ECAORF1551_1_sense) | 4.178064 | 0.013103 |  |  | ECA1583 | putative integrase |
| ECA1588(ECAORF1556_1_sense) |  |  | 1.887356 | 0.004459 | ECA1588 | conserved hypothetical protein |
| ECA1597(ECAORF1565_1_sense) | 0.607474 | 0.030181 |  |  | ECA1597 | hypothetical protein |
| ECA1598(ECAORF1566_1_sense) | 0.256253 | 0.002766 |  |  | ECA1598 | conserved hypothetical protein |
| ECA1603(ECAORF1571_1_sense) | 0.608248 | 0.001072 |  |  | arsC | arsenate reductase |
| ECA1608(ECAORF1576_1_sense) | 0.664329 | 0.024513 |  |  | mobC | mobilization protein |
| ECA1614(ECAORF1582_1_sense) | 0.64433 | 0.021358 |  |  | virB10 | putative conjugal transfer protein |
| ECA1621(ECAORF1590_1_sense) | 3.402794 | 0.009781 |  |  | virB2 | putative conjugal transfer protein |
| ECA1639(ECAORF1606_1_sense) | 0.281971 | 7.23E-04 | 0.387275 | 0.008877 | ECA1639 | hypothetical protein |
| ECA1644(ECAORF1611_3_sense) |  |  | 2.371301 | 0.003976 | ECA1644 | putative DNA-binding protein |
| ECA1648(ECAORF1615_1_sense) | 0.422407 | 0.00202 |  |  | ECA1648 | putative exported protein |
| ECA1657(ECAORF1624_1_sense) |  |  | 1.969587 | 0.032454 | ECA1657 | hypothetical protein |
| ECA1664(ECAORF1631_1_sense) |  |  | 0.556704 | 0.048945 | ECA1664 | putative lipoprotein |
| ECA1669(ECAORF1636_1_sense) | 0.476873 | 0.015292 |  |  | ECA1669 | conserved hypothetical protein |
| ECA1669A(ECAORF1637_1_sense) | 0.246064 | 0.002565 |  |  | ECA1669A | conserved hypothetical protein |
| ECA1682(ECAORF1651_1_sense) |  |  | 1.962169 | 0.04784 | dsrB | conserved hypothetical protein |
| ECA1683(ECAORF1652_1_sense) | 0.308512 | 0.009246 |  |  | trg | methyl-accepting chemotaxis protein |
| ECA1684(ECAORF1653_1_sense) | 0.622766 | 0.004459 |  |  | ECA1684 | putative membrane protein |
| ECA1689(ECAORF1659_1_sense) | 0.234757 | 0.005816 |  |  | cheA | chemotaxis protein |
| ECA1689(ECAORF1659_3_sense) | 0.220729 | 0.013033 |  |  | cheA | chemotaxis protein |
| ECA1690(ECAORF1660_1_sense) | 0.472227 | 0.0076 |  |  | cheW | chemotaxis protein |
| ECA1695(ECAORF1665_1_sense) |  |  | 0.55561 | 0.010166 | cheZ | chemotaxis protein |
| ECA1696(ECAORF1666_3_sense) | 0.608094 | 0.0485 |  |  | flhB | flagellar biosynthetic protein |
| ECA1700(ECAORF1670_1_sense) | 0.42649 | 0.001566 |  |  | flgM | negative regulator of flagellin synthesis |
| ECA1706(ECAORF1676_1_sense) | 1.707455 | 0.007032 |  |  | flgF | flagellar basal-body rod protein |
| ECA1715(ECAORF1684_1_sense) | 1.873707 | 0.002346 |  |  | fliQ | flagellar biosynthetic protein |
| ECA1729(ECAORF1698_1_sense) | 0.43232 | 0.010817 |  |  | fliS | flagellar protein |
| ECA1730(ECAORF1699_1_sense) | 0.343995 | 4.90E-06 |  |  | fliD | flagellar hook-associated protein 2 |
| ECA1731(ECAORF1700_1_sense) | 0.236522 | 0.023944 |  |  | fliC | flagellin |
| ECA1731(ECAORF1700_3_sense) | 0.263353 | 0.02349 |  |  | fliC | flagellin |
| ECA1733(ECAORF1702_3_sense) |  |  | 0.59571 | 0.007906 | vioA | nucleotide sugar transaminase |
| ECA1739(ECAORF1708_1_sense) | 0.381864 | 0.002004 |  |  | fliA | RNA polymerase sigma factor for flagellar operon |
| ECA1740(ECAORF1709_3_sense) | 0.488976 | 0.048418 | 0.34875 | 0.018204 | fliZ | putative alternative sigma factor regulatory protein |
| ECA1742(ECAORF1711_1_sense) |  |  | 1.713863 | 0.046175 | ECA1742 | conserved hypothetical protein |
| ECA1747(ECAORF1716_1_sense) |  |  | 1.612819 | 0.012174 | ECA1747 | conserved hypothetical protein |
| ECA1748(ECAORF1717_1_sense) |  |  | 0.47001 | 0.045028 | fabA | 3-hydroxydecanoyl-[acyl-carrier-protein] dehydratase |
| ECA1751(ECAORF1720_1_sense) |  |  | 1.894003 | 0.003075 | ompA | outer-membrane protein A |
| ECA1762(ECAORF1731_1_sense) | 1.832454 | 0.00406 |  |  | ECA1762 | conserved hypothetical protein |
| ECA1764(ECAORF1733_1_sense) | 0.454161 | 0.030355 | 0.605308 | 0.049825 | ECA1764 | conserved hypothetical protein |
| ECA1765(ECAORF1734_3_sense) |  |  | 1.887693 | 0.019235 | ECA1765 | hypothetical protein |
| ECA1767(ECAORF1736_1_sense) | 0.648645 | 0.011225 |  |  | ECA1767 | Isochorismatase family protein |
| ECA1769(ECAORF1738_1_sense) | 0.438093 | 0.011392 |  |  | ECA1769 | conserved hypothetical protein |
| ECA1773(ECAORF1742_1_sense) | 0.092086 | 0.016739 |  |  | ECA1773 | conserved hypothetical protein |
| ECA1774(ECAORF1743_1_sense) | 0.36892 | 0.025323 | 0.291994 | 0.012773 | ECA1774 | methyl-accepting chemotaxis protein |
| ECA1778(ECAORF1747_1_sense) | 1.973643 | 0.007171 |  |  | opgH | periplasmic glucans biosynthesis protein |
| ECA1780(ECAORF1749_1_sense) |  |  | 1.517493 | 0.003346 | ECA1780 | putative NADH-dependent flavin oxidoreductase |
| ECA1782(ECAORF1751_1_sense) | 0.096226 | 0.013142 |  |  | ECA1782 | putative exported protein |
| ECA1784(ECAORF1753_1_sense) | 0.139069 | 1.04E-05 |  |  | ECA1784 | conserved hypothetical protein |
| ECA1785(ECAORF1754_1_sense) | 0.358314 | 0.001421 |  |  | ECA1785 | conserved hypothetical protein |
| ECA1787(ECAORF1756_1_sense) |  |  | 0.55155 | 0.003617 | pyrC | dihydroorotase |
| ECA1788(ECAORF1757_1_sense) | 0.218483 | 0.022074 |  |  | ECA1788 | conserved hypothetical protein |
| ECA1788(ECAORF1757_3_sense) | 0.227054 | 0.030143 |  |  | ECA1788 | conserved hypothetical protein |
| ECA1792(ECAORF1761_1_sense) | 2.520245 | 0.004685 |  |  | ECA1792 | conserved hypothetical protein |
| ECA1794(ECAORF1763_1_sense) | 2.881139 | 2.48E-04 |  |  | plsX | fatty acid/phospholipid synthesis protein |
| ECA1795(ECAORF1764_1_sense) | 2.571209 | 2.70E-04 |  |  | fabH | 3-oxoacyl-[acyl-carrier-protein] synthase III |
| ECA1796(ECAORF1765_1_sense) | 2.004376 | 0.007228 |  |  | fabD | malonyl CoA-acyl carrier protein transacylase |
| ECA1799(ECAORF1768_1_sense) | 3.983888 | 0.009402 |  |  | fabF | 3-oxoacyl-[acyl-carrier-protein] synthase II |
| ECA1804(ECAORF1773_1_sense) | 2.486338 | 2.91E-04 |  |  | ECA1804 | conserved hypothetical protein |
| ECA1811(ECAORF1778_1_sense) |  |  | 1.715132 | 0.047513 | ECA1811 | putative lipoprotein |
| ECA1814(ECAORF1781_1_sense) | 0.178339 | 0.044115 |  |  | ECA1814 | conserved hypothetical protein |
| ECA1815(ECAORF1782_1_sense) | 3.01741 | 0.025778 |  |  | ndh | NADH dehydrogenase |
| ECA1819(ECAORF1786_1_sense) |  |  | 1.810769 | 0.009576 | ECA1819 | TetR-family transcriptional regulator |
| ECA1820(ECAORF1787_1_sense) | 3.089399 | 8.16E-04 |  |  | ECA1820 | conserved hypothetical protein |
| ECA1823(ECAORF1789_1_sense) | 2.036139 | 0.010993 |  |  | lolC | lipoprotein releasing system transmembrane protein |
| ECA1827(ECAORF1793_1_sense) | 2.648472 | 0.004145 |  |  | cobB | putative cobalamin biosynthesis/propionate catabolism protein |
| ECA1840(ECAORF1806_1_sense) | 1.783351 | 0.011458 |  |  | gntT | high-affinity gluconate transporter |
| ECA1847(ECAORF3188_1_sense) | 5.120179 | 0.019192 |  |  |  | PROBE06201 |
| ECA1850(ECAORF1816_1_sense) | 0.106545 | 2.57E-04 | 0.242648 | 0.005035 | palI | sucrose isomerase |
| ECA1853(ECAORF1819_1_sense) | 0.544419 | 0.01727 |  |  | ppsA | phosphoenolpyruvate synthase |
| ECA1855(ECAORF1821_1_sense) | 2.347569 | 4.55E-04 |  |  | ECA1855 | putative membrane protein |
| ECA1861(ECAORF1827_1_sense) | 2.50327 | 0.006594 | 2.233108 | 0.018217 | sufC | probable ABC transporter ATP-dependent protein |
| ECA1865(ECAORF1831_1_sense) | 1.457245 | 0.018459 |  |  | ECA1865 | conserved hypothetical protein |
| ECA1867(ECAORF1833_1_sense) |  |  | 2.619857 | 0.011381 | pykF | pyruvate kinase |
| ECA1875(ECAORF1841_1_sense) | 0.110265 | 1.56E-04 | 0.482923 | 3.42E-04 | nrfA | cytochrome c552 precursor |
| ECA1876(ECAORF1842_1_sense) | 0.346398 | 0.017313 |  |  | nrfB | cytochrome C-type protein |
| ECA1878(ECAORF1844_1_sense) | 0.313218 | 0.028483 |  |  | nrfD | nitrite reductase complex component |
| ECA1879(ECAORF1845_1_sense) | 0.580229 | 0.018733 |  |  | nrfE | cytochrome C-type biogenesis protein |
| ECA1879(ECAORF1845_3_sense) | 0.371608 | 0.035642 |  |  | nrfE | cytochrome C-type biogenesis protein |
| ECA1885(ECAORF1851_2_sense) | 3.555583 | 0.023934 |  |  | ccmD | heme exporter protein D |
| ECA1891(ECAORF1857_1_sense) | 1.86972 | 0.01499 |  |  | ECA1891 | hypothetical protein |
| ECA1900(ECAORF1866_1_sense) |  |  | 1.978155 | 0.006373 | narQ | nitrate/nitrite sensor protein |
| ECA1900(ECAORF1866_3_sense) |  |  | 1.713076 | 0.006066 | narQ | nitrate/nitrite sensor protein |
| ECA1909(ECAORF1875_1_sense) | 0.446871 | 0.039403 |  |  | katB | catalase-peroxidase |
| ECA1928(ECAORF1894_1_sense) | 1.462841 | 0.004814 |  |  | rnt | ribonuclease T |
| ECA1930(ECAORF1896_1_sense) | 0.561359 | 1.64E-04 |  |  | ECA1930 | conserved hypothetical protein |
| ECA1931(ECAORF1897_1_sense) | 0.243549 | 0.022883 | 0.399118 | 0.002414 | hor | global regulator |
| ECA1933(ECAORF1899_1_sense) |  |  | 1.559549 | 0.037835 | ECA1933 | conserved hypothetical protein |
| ECA1936(ECAORF1902_1_sense) | 1.872641 | 9.46E-04 |  |  | tyrS | tyrosyl-tRNA synthetase |
| ECA1938(ECAORF1904_1_sense) | 0.096355 | 0.032233 |  |  | gst | glutathione S-transferase |
| ECA1939(ECAORF1905_1_sense) | 0.34886 | 0.001655 |  |  | ECA1939 | putative glutamate symport protein |
| ECA1944(ECAORF1910_1_sense) | 2.907355 | 0.019846 |  |  | pgpB | phosphatidylglycerophosphatase B |
| ECA1947(ECAORF1911_1_sense) | 1.859359 | 0.001288 |  |  | ECA1947 | putative lipoprotein |
| ECA1957(ECAORF1921_1_sense) |  |  | 0.700737 | 0.013949 | pyrF | orotidine 5'-phosphate decarboxylase |
| ECA1964(ECAORF1928_1_sense) | 1.820684 | 0.002789 |  |  | ECA1964 | putative molybdopterin oxidoreductase |
| ECA1969(ECAORF1933_1_sense) | 10.85764 | 0.012205 | 18.76599 | 0.001343 | ECA1969 | putative NADH:flavin oxidoreductase |
| ECA1970(ECAORF1934_1_sense) | 4.957012 | 0.017537 | 5.286638 | 0.006023 | ECA1970 | putative thiamine biosynthesis protein |
| ECA1971(ECAORF1935_1_sense) | 0.243195 | 0.00118 |  |  | aidA | conserved hypothetical protein |
| ECA1980(ECAORF1944_1_sense) |  |  | 2.41188 | 0.013536 | ECA1980 | None |
| ECA1981(ECAORF1945_1_sense) | 0.516723 | 0.040689 | 0.614254 | 0.013898 | celV | endoglucanase V |
| ECA1983(ECAORF1947_1_sense) | 0.497237 | 0.006286 | 0.344801 | 0.013954 | pspA | phage shock protein A |
| ECA1990(ECAORF1954_1_sense) | 0.162383 | 0.002946 |  |  | tpx | thiol peroxidase |
| ECA1990(ECAORF1954_3_sense) | 0.162893 | 0.006305 |  |  | tpx | thiol peroxidase |
| ECA1991(ECAORF1955_1_sense) | 0.405138 | 0.016718 |  |  | ECA1991 | conserved hypothetical protein |
| ECA1992(ECAORF1956_1_sense) | 0.515572 | 8.81E-04 |  |  | mppA | periplasmic murein peptide-binding protein |
| ECA1998(ECAORF1962_1_sense) | 0.179749 | 0.001084 |  |  | ECA1998 | conserved hypothetical protein |
| ECA2005(ECAORF1969_1_sense) | 4.546782 | 0.033009 | 3.757817 | 0.019433 | acpD | acyl carrier protein phosphodiesterase |
| ECA2010(ECAORF1974_1_sense) | 0.405908 | 0.005123 |  |  | ECA2010 | putative exported protein |
| ECA2011(ECAORF1975_1_sense) | 0.151741 | 0.028856 |  |  | asr | acid shock protein |
| ECA2013(ECAORF1977_1_sense) | 2.101423 | 0.023292 | 2.396257 | 0.031279 | rstA | two-component response regulator |
| ECA2013(ECAORF1977_3_sense) | 1.941162 | 0.033644 | 2.032935 | 0.016284 | rstA | two-component response regulator |
| ECA2019(ECAORF1983_1_sense) | 0.276494 | 0.003308 | 0.307309 | 0.003219 | ECA2019 | hypothetical protein |
| ECA2023(ECAORF1988_1_sense) | 2.383635 | 0.008643 |  |  | ECA2023 | putative siderophore-interacting protein |
| ECA2026(ECAORF1991_1_sense) | 3.201086 | 0.029232 |  |  | ECA2026 | putative exported protein |
| ECA2027(ECAORF1992_1_sense) | 2.700313 | 0.007737 |  |  | ECA2027 | putative acyltransferase |
| ECA2031(ECAORF1996_1_sense) |  |  | 0.245881 | 0.010622 | narG | respiratory nitrate reductase 1 alpha chain |
| ECA2041(ECAORF2006_1_sense) | 1.643138 | 0.004039 |  |  | ECA2041 | putative DNA-binding protein |
| ECA2042(ECAORF2007_1_sense) | 0.592375 | 0.015046 |  |  | ECA2042 | hypothetical protein |
| ECA2050(ECAORF2015_1_sense) | 1.812262 | 0.011698 |  |  | ECA2050 | putative membrane protein |
| ECA2053(ECAORF2018_1_sense) | 0.385639 | 0.002504 |  |  | gabT | 4-aminobutyrate aminotransferase |
| ECA2054(ECAORF2019_3_sense) |  |  | 0.690911 | 0.022928 |  | PROBE10150 |
| ECA2058(ECAORF2023_1_sense) | 1.757847 | 0.011966 |  |  | ECA2058 | probable short-chain dehydrogenase |
| ECA2069(ECAORF2034_1_sense) | 0.552865 | 0.006333 |  |  | ECA2069 | TetR-family transcriptional regulator |
| ECA2072(ECAORF2037_1_sense) | 1.489934 | 0.00474 |  |  | ECA2072 | flavodoxin |
| ECA2082(ECAORF2047_1_sense) | 1.952053 | 0.002411 |  |  | hrpO | type III secretion protein |
| ECA2082(ECAORF2047_3_sense) | 1.758523 | 0.027229 |  |  | hrpO | type III secretion protein |
| ECA2093(ECAORF1970_1_sense) | 1.754301 | 0.013117 |  |  | hrpA | type III secretion protein |
| ECA2097(ECAORF2062_1_sense) |  |  | 0.629604 | 0.041973 | hrpE | type III secretion protein |
| ECA2098(ECAORF2063_1_sense) | 1.739192 | 0.023286 |  |  | hrpF | type III secretion protein |
| ECA2099(ECAORF2064_1_sense) |  |  | 1.870232 | 0.040561 | hrpG | type III secretion protein |
| ECA2108(ECAORF2073_1_sense) | 0.606826 | 0.034997 |  |  | ECA2108 | putative lipoprotein |
| ECA2116(ECAORF2081_1_sense) | 0.534302 | 0.00869 |  |  | hecB | putative hemolysin activator protein |
| ECA2123(ECAORF2088_3_sense) | 1.749812 | 0.020455 |  |  | ECA2123 | hypothetical protein |
| ECA2130(ECAORF2095_1_sense) |  |  | 1.741533 | 0.03079 | ECA2130 | hypothetical protein |
| ECA2134(ECAORF2099_1_sense) | 0.281472 | 0.018598 | 0.312086 | 1.17E-04 | ECA2134 | ABC transporter, substrate binding protein |
| ECA2150(ECAORF2115_1_sense) | 0.030509 | 0.004634 | 0.059521 | 0.014529 | ECA2150 | putative membrane protein |
| ECA2161(ECAORF2126_1_sense) | 0.385461 | 0.018499 |  |  | ECA2161 | putative luciferase-like monooxygenase |
| ECA2162(ECAORF2127_1_sense) | 0.244241 | 0.029368 |  |  | ECA2162 | conserved hypothetical protein |
| ECA2164(ECAORF4403_2_sense) | 0.453712 | 0.021974 |  |  |  | PROBE09119 |
| ECA2166(ECAORF2131_1_sense) | 2.06265 | 0.009221 |  |  | ascB | 6-phospho-beta-glucosidase |
| ECA2167(ECAORF2132_1_sense) | 0.548877 | 0.016612 |  |  | ECA2167 | putative inositol monophosphatase |
| ECA2168(ECAORF2133_1_sense) | 1.74206 | 0.029451 |  |  | ECA2168 | conserved hypothetical protein |
| ECA2172(ECAORF2137_1_sense) | 0.401604 | 0.009921 |  |  | ECA2172 | putative exported protein |
| ECA2173(ECAORF2138_1_sense) | 2.11821 | 0.02718 |  |  | ECA2173 | conserved hypothetical protein |
| ECA2175(ECAORF2140_1_sense) | 0.458625 | 0.001451 |  |  | ECA2175 | putative exported protein |
| ECA2176(ECAORF2141_1_sense) | 0.435886 | 3.91E-04 |  |  | ECA2176 | hypothetical protein |
| ECA2182(ECAORF2146_1_sense) | 1.670462 | 0.02962 |  |  | ECA2182 | prophage integrase |
| ECA2183(ECAORF2147_1_sense) | 2.736218 | 0.039432 |  |  | ECA2183 | conserved hypothetical protein |
| ECA2184(ECAORF2148_1_sense) | 1.920493 | 0.005681 |  |  | pth | peptidyl-tRNA hydrolase |
| ECA2186(ECAORF2150_1_sense) | 2.122491 | 0.03466 |  |  | prs | ribose-phosphate pyrophosphokinase |
| ECA2189(ECAORF2153_1_sense) | 2.402031 | 6.68E-04 |  |  | hemA | glutamyl-tRNA reductase |
| ECA2204(ECAORF2167_1_sense) | 0.438032 | 5.05E-04 |  |  | pntA | pyridine nucleotide transhydrogenase subunit-alpha |
| ECA2206(ECAORF2169_1_sense) | 0.242655 | 0.00687 |  |  | ECA2206 | putative universal stress protein |
| ECA2207(ECAORF2170_1_sense) | 2.88975 | 0.007847 |  |  | fnr | fumarate and nitrate reduction regulatory protein |
| ECA2220(ECAORF2183_1_sense) | 0.044407 | 0.005443 |  |  | ECA2220 | putative cellulase |
| ECA2221(ECAORF2184_1_sense) | 0.303954 | 4.28E-04 |  |  | osmC | osmotically inducible protein C |
| ECA2221(ECAORF2184_3_sense) | 0.323584 | 0.013207 |  |  | osmC | osmotically inducible protein C |
| ECA2225A(ECAORF2189_2_sense) | 0.606328 | 0.04455 |  |  | ECA2225A | hypothetical protein |
| ECA2237(ECAORF2202_1_sense) | 0.169703 | 0.011743 |  |  | flxA | conserved hypothetical protein |
| ECA2240(ECAORF2205_1_sense) | 2.286271 | 0.026205 |  |  | ECA2240 | putative lipoprotein |
| ECA2244(ECAORF2209_1_sense) |  |  | 0.375268 | 0.023832 | ddc | L-2,4-diaminobutyrate decarboxylase |
| ECA2245(ECAORF2210_1_sense) | 0.256338 | 0.033373 |  |  | ECA2245 | conserved hypothetical protein |
| ECA2254(ECAORF2219_1_sense) | 2.794428 | 0.015263 |  |  | ECA2254 | putative lipoprotein |
| ECA2255(ECAORF2220_1_sense) |  |  | 1.981614 | 0.030226 | ECA2255 | putative exported protein |
| ECA2262(ECAORF2227_3_sense) |  |  | 0.549958 | 0.009731 | fumC | fumarate hydratase class II |
| ECA2279(ECAORF2244_1_sense) | 4.848857 | 0.030676 |  |  | rnfD | electron transport complex protein |
| ECA2279(ECAORF2244_3_sense) | 4.744425 | 0.012401 |  |  | rnfD | electron transport complex protein |
| ECA2280(ECAORF2245_1_sense) | 2.513631 | 0.003009 |  |  | rnfG | electron transport complex protein |
| ECA2281(ECAORF2246_1_sense) | 2.535569 | 0.022737 |  |  | rnfE | electron transport complex protein |
| ECA2282(ECAORF2247_1_sense) | 1.960977 | 0.013925 |  |  | nth | endonuclease III |
| ECA2283(ECAORF2248_1_sense) | 1.585529 | 0.005905 |  |  | ECA2283 | putative proton dependent peptide transporter |
| ECA2284(ECAORF2249_1_sense) | 0.341623 | 0.009722 |  |  | cysB | cys regulon transcriptional activator |
| ECA2288(ECAORF2253_1_sense) | 0.587191 | 0.01186 |  |  | sohB | protease |
| ECA2290(ECAORF2255_1_sense) | 0.683559 | 0.031273 |  |  | btuR | cob(I)alamin adenosyltransferase |
| ECA2291(ECAORF2256_1_sense) | 2.719001 | 0.00694 |  |  | ECA2291 | putative RNA pseudouridylate synthase |
| ECA2293(ECAORF2258_1_sense) | 2.473329 | 0.014465 |  |  | trpH | putative phosphoesterase |
| ECA2302(ECAORF2267_1_sense) | 0.124301 | 0.001609 | 0.225489 | 0.004955 | ECA2302 | putative oligogalacturonate-specific porin |
| ECA2305(ECAORF2270_1_sense) | 2.032906 | 0.03738 |  |  | ECA2305 | putative membrane protein |
| ECA2313(ECAORF2278_1_sense) | 2.183418 | 1.31E-05 | 1.779466 | 4.35E-05 | ispZ | intracellular septation protein |
| ECA2316(ECAORF2281_1_sense) | 0.308304 | 0.001668 |  |  | ECA2316 | conserved hypothetical protein |
| ECA2320(ECAORF2285_1_sense) | 2.24903 | 0.022853 |  |  | oppF | oligopeptide transport ATP-binding protein |
| ECA2320(ECAORF2285_3_sense) | 2.566626 | 0.011878 |  |  | oppF | oligopeptide transport ATP-binding protein |
| ECA2322(ECAORF2287_1_sense) | 2.261164 | 0.001121 |  |  | oppC | oligopeptide transport system permease protein |
| ECA2323(ECAORF2288_1_sense) | 2.795102 | 8.84E-04 |  |  | oppB | oligopeptide transport system permease protein |
| ECA2326(ECAORF2291_1_sense) | 0.183972 | 3.56E-04 |  |  | adhE | aldehyde-alcohol dehydrogenase |
| ECA2334(ECAORF2299_1_sense) | 2.203819 | 0.001188 |  |  | ECA2334 | putative membrane protein |
| ECA2340(ECAORF2305_1_sense) | 1.997523 | 0.004635 |  |  | ansA | L-asparaginase I |
| ECA2344(ECAORF2309_1_sense) |  |  | 1.704039 | 0.006445 | gapA | glyceraldehyde 3-phosphate dehydrogenase a |
| ECA2344(ECAORF2309_3_sense) |  |  | 1.536788 | 0.022532 | gapA | glyceraldehyde 3-phosphate dehydrogenase a |
| ECA2345(ECAORF2310_1_sense) |  |  | 2.840234 | 7.89E-05 | ECA2345 | putative aldose 1-epimerase |
| ECA2349(ECAORF2314_1_sense) | 0.367129 | 0.017969 |  |  | ECA2349 | conserved hypothetical protein |
| ECA2350(ECAORF2315_1_sense) | 2.211209 | 0.004647 |  |  | ECA2350 | probable transport protein |
| ECA2360(ECAORF2325_1_sense) | 1.822113 | 0.028732 |  |  | fadR | fatty acid metabolism regulator |
| ECA2364(ECAORF2329_1_sense) | 0.604689 | 0.018822 |  |  | ECA2364 | putative lipoprotein |
| ECA2365(ECAORF2330_1_sense) | 1.769663 | 6.04E-04 |  |  | ECA2365 | conserved hypothetical protein |
| ECA2369(ECAORF2334_1_sense) | 0.504904 | 0.005382 |  |  | minD | septum site-determining protein |
| ECA2371(ECAORF2336_1_sense) | 2.100502 | 0.012326 | 2.009718 | 0.022798 | rnd | ribonuclease D |
| ECA2373(ECAORF2338_1_sense) | 5.642025 | 0.001256 |  |  | ECA2373 | outer membrane lipoprotein |
| ECA2374(ECAORF2339_1_sense) | 3.00701 | 4.11E-04 |  |  | ECA2374 | conserved hypothetical protein |
| ECA2375(ECAORF2340_1_sense) | 1.598612 | 0.038973 |  |  | ECA2375 | putative ATP-dependent helicase |
| ECA2380(ECAORF2345_1_sense) |  |  | 0.526391 | 0.037688 | sdaA | L-serine dehydratase 1 |
| ECA2381(ECAORF2346_1_sense) | 0.543249 | 0.022552 |  |  | ECA2381 | LysR-family transcriptional regulator |
| ECA2382(ECAORF2347_1_sense) | 1.909267 | 0.020787 |  |  | ECA2382 | putative NADP oxidoreductase coenzyme F420-dependent protein |
| ECA2384(ECAORF2349_1_sense) | 4.970467 | 0.021913 |  |  | ECA2384 | putative membrane protein |
| ECA2389(ECAORF2354_1_sense) | 1.99755 | 0.003838 |  |  | ECA2389 | putative membrane protein |
| ECA2389(ECAORF2354_3_sense) | 1.935121 | 0.023265 |  |  | ECA2389 | putative membrane protein |
| ECA2390(ECAORF2355_1_sense) | 1.746807 | 0.025576 |  |  | rrmA | ribosomal RNA large subunit methyltransferase A |
| ECA2391(ECAORF2356_1_sense) | 0.471886 | 0.043494 |  |  | ECA2391 | cold shock protein |
| ECA2391(ECAORF2356_2_sense) | 1.746546 | 0.025546 |  |  | ECA2391 | cold shock protein |
| ECA2396(ECAORF2361_1_sense) | 0.223935 | 0.002052 |  |  | ECA2396 | putative fructosamine kinase |
| ECA2406(ECAORF2371_1_sense) | 0.578055 | 0.008295 |  |  | togB | periplasmic pectic oligomers binding protein |
| ECA2410(ECAORF2375_1_sense) | 1.806823 | 0.022679 |  |  | ECA2410 | putative sodium:dicarboxylate symporter |
| ECA2411(ECAORF2376_1_sense) | 0.564757 | 5.93E-04 |  |  | osmE | osmotically inducible lipoprotein E |
| ECA2417(ECAORF2382_1_sense) | 3.368386 | 0.002507 |  |  | pheT | phenylalanyl-tRNA synthetase beta chain |
| ECA2418(ECAORF2383_1_sense) | 2.676501 | 5.41E-04 |  |  | pheS | phenylalanyl-tRNA synthetase alpha chain |
| ECA2419(ECAORF2384_1_sense) | 1.658545 | 0.001156 |  |  | rplT | 50S ribosomal protein L20 |
| ECA2422(ECAORF2387_1_sense) | 2.960892 | 5.19E-04 |  |  | thrS | threonyl-tRNA synthetase |
| ECA2424(ECAORF2389_1_sense) | 1.533639 | 0.011861 |  |  | ECA2424 | putative exported protein |
| ECA2425(ECAORF2390_1_sense) | 2.11467 | 0.009372 |  |  | kdgR | pectin degradation repressor |
| ECA2430(ECAORF2395_1_sense) | 0.165198 | 0.008399 |  |  | ECA2430 | putative membrane protein |
| ECA2433(ECAORF2398_1_sense) | 0.512841 | 0.037089 |  |  | ECA2433 | putative signaling protein |
| ECA2434(ECAORF2399_1_sense) | 0.320406 | 0.039982 |  |  | ECA2434 | hypothetical protein |
| ECA2435(ECAORF2400_1_sense) | 1.642069 | 0.028053 | 1.518776 | 9.73E-04 | rdgA | regulator of pectin lyase production |
| ECA2439(ECAORF2403_1_sense) | 0.233126 | 0.001617 |  |  | icd | isocitrate dehydrogenase [NADP] |
| ECA2442(ECAORF2406_1_sense) | 2.155385 | 0.037764 |  |  | trmU | tRNA (5-methylaminomethyl-2-thiouridylate)-methyltransferase |
| ECA2445(ECAORF2409_1_sense) | 2.257666 | 0.012341 |  |  | pehR | two-component response regulator of virulence determinants |
| ECA2447(ECAORF2411_1_sense) |  |  | 2.040851 | 0.011228 | ECA2447 | conserved hypothetical protein |
| ECA2450(ECAORF2413_1_sense) | 2.189378 | 0.01954 |  |  | potA | spermidine/putrescine transport ATP-binding protein |
| ECA2451(ECAORF2414_1_sense) | 1.849839 | 5.53E-04 |  |  | potB | spermidine/putrescine transport system permease protein |
| ECA2452(ECAORF2415_1_sense) | 2.623876 | 0.014122 |  |  | potC | spermidine/putrescine transport system permease protein |
| ECA2453(ECAORF2416_1_sense) |  |  | 0.670623 | 0.032207 | potD | spermidine/putrescine-binding periplasmic protein |
| ECA2454(ECAORF2171_1_sense) | 1.66421 | 0.005629 |  |  | ogt | probable methylated DNA--protein-cysteine methyltransferase |
| ECA2459(ECAORF2422_1_sense) | 1.487966 | 0.005325 |  |  | prc | tail-specific protease precursor |
| ECA2460(ECAORF2423_1_sense) | 2.433613 | 9.88E-04 |  |  | proQ | ProP effector |
| ECA2464(ECAORF2427_1_sense) | 2.178116 | 0.02639 |  |  | ECA2464 | conserved hypothetical protein |
| ECA2471(ECAORF2434_1_sense) | 22.75917 | 0.015597 |  |  | cspG | cold shock protein |
| ECA2471(ECAORF2434_2_sense) | 23.99014 | 0.018933 |  |  | cspG | cold shock protein |
| ECA2471(ECAORF2434_3_sense) | 22.51827 | 0.018416 |  |  | cspG | cold shock protein |
| ECA2475(ECAORF2328_1_sense) | 0.448566 | 0.027112 |  |  | holE | DNA polymerase III, theta subunit |
| ECA2475(ECAORF2437_1_sense) | 2.245784 | 4.64E-05 |  |  |  | PROBE04745 |
| ECA2480(ECAORF2442_1_sense) | 0.343517 | 0.005506 |  |  | hexR | hex regulon repressor |
| ECA2482(ECAORF2444_1_sense) | 1.754105 | 0.017447 |  |  | msbB | lipid A biosynthesis (KDO)2-(lauroyl)-lipid iva acyltransferase |
| ECA2483(ECAORF2445_1_sense) | 1.714123 | 0.009953 |  |  | ECA2483 | putative peptidase |
| ECA2485(ECAORF2447_1_sense) | 1.523953 | 0.02216 |  |  | znuC | high-affinity zinc uptake system ATP-binding protein |
| ECA2492(ECAORF2454_1_sense) | 1.871882 | 0.011203 |  |  | ruvA | holliday junction DNA helicase |
| ECA2493(ECAORF2455_1_sense) | 1.610614 | 0.006328 |  |  | ruvC | crossover junction endodeoxyribonuclease |
| ECA2495(ECAORF2457_1_sense) | 2.356402 | 0.015095 | 1.637954 | 0.006559 | nudB | DATP pyrophosphohydrolase |
| ECA2506(ECAORF2468_1_sense) | 0.591032 | 0.011473 |  |  | ECA2506 | hypothetical protein |
| ECA2511(ECAORF2472_1_sense) | 3.260365 | 0.003562 |  |  | ECA2511 | conserved hypothetical protein |
| ECA2521(ECAORF2482_1_sense) | 2.755178 | 0.00456 |  |  | mviN | putative virulence factor |
| ECA2525(ECAORF2486_1_sense) | 2.267624 | 0.018504 | 1.589603 | 0.004878 | ECA2525 | putative membrane protein |
| ECA2527(ECAORF2488_1_sense) |  |  | 1.883597 | 0.004517 | ECA2527 | putative lipoprotein |
| ECA2528(ECAORF2489_1_sense) |  |  | 1.605249 | 0.002735 | ECA2528 | conserved hypothetical protein |
| ECA2534(ECAORF2495_1_sense) | 1.606608 | 7.24E-04 |  |  | uup | ABC transporter ATP-binding protein |
| ECA2539(ECAORF2500_1_sense) |  |  | 2.525115 | 0.020161 | pepN | aminopeptidase N |
| ECA2542(ECAORF2503_1_sense) | 0.464379 | 0.018488 |  |  | ompN | outer membrane protein |
| ECA2545(ECAORF2506_1_sense) | 2.029531 | 0.018054 |  |  | ECA2545 | putative exported protein |
| ECA2548(ECAORF2509_1_sense) | 1.702337 | 1.99E-04 |  |  | mukE | killing factor |
| ECA2549(ECAORF2510_1_sense) | 1.577654 | 0.015885 |  |  | mukF | killing factor |
| ECA2553(ECAORF2514_1_sense) | 0.089666 | 4.91E-05 | 0.135984 | 0.002848 | ECA2553 | pectate lyase |
| ECA2556(ECAORF2517_1_sense) | 2.351879 | 0.04071 |  |  | lpxK | tetraacyldisaccharide 4'-kinase |
| ECA2556(ECAORF2517_3_sense) | 2.285227 | 0.002153 |  |  | lpxK | tetraacyldisaccharide 4'-kinase |
| ECA2557(ECAORF2518_1_sense) | 1.779379 | 0.001659 |  |  | msbA | lipid a export ATP-binding/permease |
| ECA2562(ECAORF2523_1_sense) | 2.096433 | 0.004151 |  |  | ECA2562 | putative iron ABC transporter permease component |
| ECA2565(ECAORF2526_1_sense) | 1.469441 | 0.005744 |  |  | sbcB | exodeoxyribonuclease I |
| ECA2566(ECAORF2527_1_sense) | 2.423324 | 0.006245 |  |  | ECA2566 | putative amino acid permease |
| ECA2567(ECAORF2528_1_sense) |  |  | 0.334669 | 0.007042 | ECA2567 | hypothetical protein |
| ECA2573(ECAORF2534_1_sense) |  |  | 2.034813 | 0.035293 | citD | citrate lyase acyl carrier protein |
| ECA2576(ECAORF2537_1_sense) | 0.32333 | 0.034792 |  |  | citW | citrate/acetate antiporter |
| ECA2578(ECAORF2539_1_sense) | 0.636905 | 0.009049 |  |  | citB | two-component response regulator |
| ECA2579(ECAORF2540_1_sense) | 0.450274 | 0.014491 |  |  | ECA2579 | putative methyl-accepting chemotaxis citrate transducer |
| ECA2586(ECAORF2547_1_sense) |  |  | 0.663719 | 0.036761 | hisH | imidazole glycerol phosphate synthase subunit |
| ECA2590(ECAORF2551_1_sense) | 6.359778 | 0.008964 |  |  | ihfB | integration host factor beta-subunit |
| ECA2595(ECAORF2556_1_sense) | 1.720322 | 0.021801 | 0.624628 | 0.01907 | ECA2595 | conserved hypothetical protein |
| ECA2597(ECAORF2558_1_sense) |  |  | 2.926844 | 7.27E-04 | pflB | formate acetyltransferase |
| ECA2598(ECAORF2559_1_sense) | 2.420766 | 2.94E-05 |  |  | ECA2598 | putative exported protein |
| ECA2617(ECAORF2578_1_sense) | 2.699048 | 6.14E-04 |  |  | ECA2617 | phage lysis protein Y, holin |
| ECA2640(ECAORF2601_1_sense) | 2.264838 | 6.07E-04 |  |  | ECA2640 | conserved hypothetical protein |
| ECA2641(ECAORF2602_1_sense) |  |  | 1.465635 | 0.047313 | ECA2641 | permease |
| ECA2648(ECAORF2609_1_sense) | 0.605845 | 0.008136 |  |  | lrp | leucine-responsive regulatory protein |
| ECA2652(ECAORF2613_1_sense) | 2.204333 | 0.049722 |  |  | aat | leucyl/phenylalanyl-tRNA--protein transferase |
| ECA2657(ECAORF2618_1_sense) |  |  | 3.554892 | 0.045725 | ECA2657 | conserved hypothetical protein |
| ECA2671(ECAORF2632_1_sense) | 0.430549 | 0.023559 |  |  | artJ | arginine-binding periplasmic protein 2 |
| ECA2671(ECAORF2632_3_sense) | 0.422018 | 0.017064 |  |  | artJ | arginine-binding periplasmic protein 2 |
| ECA2678(ECAORF2639_1_sense) | 2.078874 | 0.030915 |  |  | ECA2678 | conserved hypothetical protein |
| ECA2679(ECAORF2640_1_sense) | 1.461284 | 0.038733 |  |  | rimK | ribosomal protein S6 modification protein |
| ECA2681(ECAORF2642_1_sense) | 0.417637 | 0.003545 |  |  | grxA | glutaredoxin 1 |
| ECA2708(ECAORF2669_1_sense) | 3.552259 | 2.53E-04 |  |  | ECA2708 | conserved hypothetical protein |
| ECA2709(ECAORF2670_1_sense) |  |  | 1.912302 | 0.00238 | sanA | putative vancomycin resistance protein |
| ECA2714(ECAORF2675_1_sense) | 1.75419 | 0.004943 |  |  | adhC | alcohol dehydrogenase class III |
| ECA2724(ECAORF2685_1_sense) | 3.298711 | 0.012712 | 2.888142 | 0.00118 | rscR | LysR-family transciptional regulator |
| ECA2725(ECAORF2686_1_sense) | 1.751898 | 0.026357 |  |  | ECA2725 | putative membrane protein |
| ECA2730(ECAORF2691_1_sense) | 2.996583 | 0.002964 |  |  | sotA | sugar efflux transporter |
| ECA2733(ECAORF2694_1_sense) | 1.691188 | 0.003347 |  |  | ECA2733 | conserved hypothetical protein |
| ECA2734(ECAORF2695_1_sense) | 2.518496 | 0.025621 |  |  | ECA2734 | putative membrane-bound phosphatase |
| ECA2734(ECAORF2695_3_sense) | 2.437491 | 0.011964 |  |  | ECA2734 | putative membrane-bound phosphatase |
| ECA2735(ECAORF2696_1_sense) | 1.994761 | 0.038525 |  |  | spr | lipoprotein |
| ECA2736(ECAORF2697_1_sense) | 0.576462 | 0.01447 |  |  | ECA2736 | putative ABC transporter, periplasmic binding protein |
| ECA2741(ECAORF2702_1_sense) | 1.742503 | 0.003987 |  |  | bcr | bicyclomycin resistance protein |
| ECA2742(ECAORF2703_1_sense) | 3.15381 | 1.19E-04 | 1.934771 | 2.96E-04 | rsuA | ribosomal small subunit pseudouridine synthase |
| ECA2747(ECAORF2708_1_sense) | 1.916134 | 0.002449 |  |  | ECA2747 | nucleoid-associated protein |
| ECA2752(ECAORF2713_2_sense) | 1.603044 | 0.025808 |  |  | ECA2752 | phage-related protein |
| ECA2756(ECAORF2717_1_sense) | 0.400595 | 0.003418 |  |  | ECA2756 | hypothetical protein |
| ECA2761(ECAORF2722_1_sense) | 0.566897 | 0.006637 |  |  | ECA2761 | conserved hypothetical protein |
| ECA2763(ECAORF2724_1_sense) | 2.015512 | 0.010936 |  |  | ECA2763 | probable transport protein |
| ECA2770(ECAORF2731_1_sense) | 1.461126 | 0.028395 |  |  | glnQ | glutamine transport ATP-binding protein |
| ECA2772(ECAORF2733_1_sense) | 1.732858 | 0.027707 | 0.601285 | 0.004855 | ECA2772 | putative glycosyl transferase |
| ECA2772(ECAORF2733_3_sense) |  |  | 0.446784 | 0.030198 | ECA2772 | putative glycosyl transferase |
| ECA2773(ECAORF2734_1_sense) | 1.757899 | 0.009547 | 0.442573 | 7.49E-04 | ECA2773 | bacteriophage tail sheath protein (partial) |
| ECA2774(ECAORF2735_1_sense) |  |  | 0.443171 | 0.009967 | ECA2774 | conserved hypothetical protein |
| ECA2775(ECAORF2736_1_sense) |  |  | 0.540152 | 0.027928 | ECA2775 | conserved hypothetical protein |
| ECA2780(ECAORF2741_1_sense) |  |  | 0.469646 | 0.015612 | ECA2780 | putative membrane protein |
| ECA2781(ECAORF2742_1_sense) | 0.319388 | 0.026167 | 0.360576 | 0.00908 | prtF | protease secretion protein |
| ECA2783(ECAORF2744_1_sense) |  |  | 0.531246 | 0.002379 | prtD | protease secretion ATP-binding protein |
| ECA2784(ECAORF2745_1_sense) | 0.307103 | 0.019094 |  |  | inh | protease inhibitor |
| ECA2785(ECAORF2746_1_sense) | 0.205486 | 4.33E-04 | 0.221227 | 0.003789 | prtW | metalloprotease |
| ECA2794(ECAORF2755_1_sense) |  |  | 1.771221 | 4.41E-04 | ECA2794 | putative exported protein |
| ECA2795(ECAORF2756_1_sense) | 0.272374 | 0.037682 |  |  | cyaB | adenylate cyclase |
| ECA2795(ECAORF2756_3_sense) | 0.338694 | 0.002895 |  |  | cyaB | adenylate cyclase |
| ECA2805(ECAORF2766_1_sense) | 5.159679 | 0.016893 |  |  | rhlE | putative ATP-dependent RNA helicase |
| ECA2805(ECAORF2766_3_sense) | 6.210567 | 0.011388 |  |  | rhlE | putative ATP-dependent RNA helicase |
| ECA2806(ECAORF2767_1_sense) | 1.490041 | 0.045621 |  |  | cbrD | achromobactin transport ATP-binding protein |
| ECA2810(ECAORF2771_1_sense) | 2.00724 | 0.007237 |  |  | acr | TonB-dependent ferric achromobactin receptor |
| ECA2812(ECAORF2773_1_sense) | 0.518702 | 0.002342 |  |  | ECA2812 | putative membrane protein |
| ECA2812(ECAORF2773_3_sense) | 0.526062 | 0.007607 |  |  | ECA2812 | putative membrane protein |
| ECA2816(ECAORF2777_1_sense) | 0.562692 | 0.005466 |  |  | moaB | molybdenum cofactor biosynthesis protein B |
| ECA2819(ECAORF2780_1_sense) | 1.57135 | 0.048607 |  |  | ECA2819 | hypothetical protein |
| ECA2823(ECAORF2784_1_sense) | 2.50064 | 0.028758 |  |  | bioC | biotin synthesis protein |
| ECA2825(ECAORF2786_1_sense) | 5.111369 | 8.75E-06 |  |  | bioB | biotin synthase |
| ECA2825(ECAORF2786_3_sense) | 5.238044 | 0.009888 |  |  | bioB | biotin synthase |
| ECA2826(ECAORF2787_1_sense) | 2.395163 | 6.65E-04 |  |  | bioA | adenosylmethionine-8-amino-7-oxononanoate aminotransferase |
| ECA2827(ECAORF2788_1_sense) | 0.10827 | 0.005178 | 0.26557 | 0.003012 | celB | beta(1,4)-glucan glucanohydrolase |
| ECA2828(ECAORF2789_1_sense) |  |  | 1.951331 | 0.047333 | ECA2828 | putative membrane protein |
| ECA2829(ECAORF2790_1_sense) | 3.57879 | 0.033256 | 4.001922 | 0.002617 | ECA2829 | puatative membrane protein |
| ECA2837(ECAORF2798_1_sense) |  |  | 1.923471 | 0.006418 | moeB | molybdopterin biosynthesis protein |
| ECA2847(ECAORF2808_1_sense) | 0.518638 | 0.031736 |  |  | ECA2847 | conserved hypothetical protein |
| ECA2851(ECAORF2812_1_sense) |  |  | 1.702748 | 0.034893 | mvpT | putative plasmid protein |
| ECA2855(ECAORF2816_1_sense) | 3.258293 | 0.043703 |  |  | ECA2855 | conserved hypothetical protein |
| ECA2866(ECAORF2827_1_sense) | 0.047295 | 0.008115 | 0.045852 | 1.18E-04 | *hcp3* | HcpA homologue |
| ECA2866(ECAORF2827_2_sense) | 0.036697 | 5.41E-04 | 0.052468 | 5.73E-06 | *hcp3* | HcpA homologue |
| ECA2866(ECAORF2827_3_sense) | 0.074201 | 2.58E-06 | 0.054696 | 6.56E-05 | *hcp3* | HcpA homologue |
| ECA2867(ECAORF2828_1_sense) | 0.292798 | 0.003119 | 0.215708 | 0.021948 | vgrG | putative RHS accessory genetic element |
| ECA2867(ECAORF4236_1_sense) | 0.318397 | 0.04953 | 0.272353 | 0.042454 | vgrG | putative RHS accessory genetic element |
| ECA2868(ECAORF2829_1_sense) | 0.49873 | 0.007054 | 0.418421 | 0.043472 | ECA2868 | conserved hypothetical protein |
| ECA2868(ECAORF2829_3_sense) | 0.525758 | 0.023475 | 0.367277 | 0.003987 | ECA2868 | conserved hypothetical protein |
| ECA2869(ECAORF2830_1_sense) | 0.372715 | 0.004844 |  |  | rhs | putative RHS protein |
| ECA2870(ECAORF2831_1_sense) | 0.140518 | 0.004007 | 0.285662 | 0.02958 | ECA2870 | hypothetical protein |
| ECA2874(ECAORF2835_1_sense) | 1.806574 | 0.049676 |  |  | ECA2874 | conserved hypothetical protein |
| ECA2875(ECAORF2836_1_sense) | 2.166432 | 3.23E-04 |  |  | ECA2875 | putative ATP-binding protein |
| ECA2879(ECAORF2840_3_sense) | 1.852232 | 0.021072 |  |  | ECA2879 | integrase |
| ECA2882(ECAORF2843_1_sense) | 0.441938 | 2.48E-04 | 0.567179 | 0.005893 |  |  |
| ECA2884(ECAORF2845_1_sense) | 2.080184 | 0.038353 |  |  | ECA2884 | hypothetical protein |
| ECA2885(ECAORF2846_1_sense) | 2.919101 | 0.0355 |  |  | cycA | D-serine/D-alanine/glycine transporter |
| ECA2886(ECAORF2847_1_sense) | 0.157159 | 0.003829 | 0.388791 | 0.034733 | ECA2886 | conserved hypothetical protein |
| ECA2886(ECAORF2847_3_sense) | 0.157211 | 0.012879 |  |  | ECA2886 | conserved hypothetical protein |
| ECA2890(ECAORF2851_1_sense) | 0.637989 | 0.046332 |  |  | ECA2890 | hypothetical protein |
| ECA2895(ECAORF2856_1_sense) |  |  | 0.330929 | 0.005497 | ECA2895 | hypothetical protein |
| ECA2897(ECAORF2858_1_sense) | 0.090541 | 0.011269 |  |  | ECA2897 | conserved hypothetical protein |
| ECA2900(ECAORF2861_1_sense) | 0.411487 | 0.004099 |  |  | ECA2900 | hypothetical protein |
| ECA2910(ECAORF2871_2_sense) | 0.359372 | 0.00945 |  |  | ECA2910 | putative plasmid replication protein |
| ECA2921(ECAORF2882_1_sense) | 0.189547 | 0.011533 | 0.268655 | 0.001423 | ECA2921 | hypothetical protein |
| ECA2946(ECAORF3198_1_sense) |  |  | 1.985468 | 0.020698 |  | PROBE06217 |
| ECA2949(ECAORF2910_1_sense) | 1.762946 | 0.035593 |  |  | nifE | nitrogenase iron-molybdenum cofactor biosynthesis protein |
| ECA2964(ECAORF2925_1_sense) | 0.608802 | 0.029216 |  |  | ECA2964 | putative peptidase |
| ECA2965(ECAORF2926_1_sense) |  |  | 2.512368 | 0.024941 | ECA2965 | putative mandelate racemase / muconate lactonizing enzyme |
| ECA2979(ECAORF2940_1_sense) |  |  | 1.514508 | 0.006797 | ECA2979 | probable hydrolase |
| ECA2987(ECAORF2948_1_sense) | 2.089075 | 2.60E-05 |  |  | ECA2987 | conserved hypothetical protein |
| ECA2989(ECAORF2950_1_sense) | 0.377005 | 0.018553 | 0.435896 | 0.003834 | nirE | uroporphyrin-III C-methyltransferase |
| ECA3005(ECAORF2965_1_sense) | 3.464913 | 0.04141 |  |  | ECA3005 | putative glutamine amidotransferase |
| ECA3008(ECAORF2968_3_sense) | 1.739601 | 0.01869 |  |  | ECA3008 | conserved hypothetical protein |
| ECA3010(ECAORF2970_1_sense) | 3.962611 | 0.012974 |  |  | ECA3010 | ABC transporter ATP-binding protein |
| ECA3011(ECAORF2971_1_sense) |  |  | 1.761887 | 0.035469 | pab | conserved hypothetical protein |
| ECA3016(ECAORF2977_1_sense) | 1.563588 | 0.015205 |  |  | nuoN | NADH-quinone oxidoreductase chain N |
| ECA3017(ECAORF2978_1_sense) | 3.233265 | 7.41E-04 | 2.844619 | 8.35E-04 | nuoM | NADH-quinone oxidoreductase chain M |
| ECA3018(ECAORF2979_1_sense) | 2.909504 | 0.013392 |  |  | nuoL | NADH-quinone oxidoreductase chain L |
| ECA3019(ECAORF2980_1_sense) | 2.123078 | 6.34E-04 | 1.909947 | 0.001834 | nuoK | NADH-quinone oxidoreductase chain K |
| ECA3020(ECAORF2981_1_sense) | 1.938508 | 0.001045 |  |  | nuoJ | NADH-quinone oxidoreductase chain J |
| ECA3023(ECAORF2984_1_sense) | 2.044567 | 0.035674 |  |  | nuoG | NADH-quinone oxidoreductase chain G |
| ECA3025(ECAORF2986_1_sense) | 1.566604 | 0.004586 |  |  | nuoE | NADH-quinone oxidoreductase chain E |
| ECA3026(ECAORF2987_1_sense) | 1.70237 | 0.001719 |  |  | nuoC | NADH-quinone oxidoreductase chain C/D |
| ECA3028(ECAORF2989_1_sense) | 2.283588 | 0.013032 |  |  | nuoA | NADH-quinone oxidoreductase chain A |
| ECA3034(ECAORF2995_1_sense) |  |  | 1.693385 | 0.005366 | ECA3034 | putative phosphohydrolase |
| ECA3036(ECAORF2997_1_sense) | 0.245321 | 0.042984 |  |  | ECA3036 | putative haloacid dehalogenase-like hydrolase |
| ECA3040(ECAORF3001_1_sense) | 2.664574 | 0.047696 |  |  | pta | phosphate acetyltransferase |
| ECA3043(ECAORF3004_1_sense) | 0.393882 | 0.007699 |  |  | ECA3043 | putative phosphoesterase |
| ECA3052(ECAORF3013_1_sense) | 2.097096 | 2.42E-04 |  |  | purF | amidophosphoribosyltransferase |
| ECA3053(ECAORF3014_1_sense) | 2.162477 | 0.007564 |  |  | cvpA | colicin V production protein |
| ECA3055(ECAORF3016_1_sense) | 2.046812 | 0.030129 |  |  | folC | bifunctional protein [includes: folylpolyglutamate synthase and dihydrofolate synthase] |
| ECA3058(ECAORF3019_1_sense) | 2.672446 | 0.01045 |  |  | truA | tRNA pseudouridine synthase A |
| ECA3063(ECAORF3024_1_sense) | 1.585952 | 2.44E-04 |  |  | fabB | 3-oxoacyl-[acyl-carrier-protein] synthase I |
| ECA3066(ECAORF3027_3_sense) | 1.503728 | 0.025282 |  |  | ECA3066 | conserved hypothetical protein |
| ECA3071(ECAORF3032_1_sense) | 0.386981 | 0.021179 |  |  | ECA3071 | conserved hypothetical protein |
| ECA3083(ECAORF3045_1_sense) | 1.659775 | 0.002805 |  |  | vacJ | lipoprotein |
| ECA3087(ECAORF3049_1_sense) | 0.029094 | 3.07E-04 | 0.091002 | 0.002306 | ECA3087 | putative exported protein |
| ECA3090(ECAORF3052_1_sense) | 0.234064 | 0.011415 | 0.420423 | 0.014048 | ECA3090 | hypothetical protein |
| ECA3092(ECAORF3054_1_sense) | 5.423291 | 4.91E-05 | 2.611666 | 0.001914 | dbpA | ATP-independent RNA helicase |
| ECA3097(ECAORF3059_1_sense) | 5.462231 | 0.036942 |  |  | ECA3097 | monooxygenase |
| ECA3100(ECAORF3062_1_sense) |  |  | 0.651915 | 0.004533 | outM | general secretion pathway protein M |
| ECA3100(ECAORF3062_3_sense) | 0.377344 | 0.024046 | 0.45107 | 0.014929 | outM | general secretion pathway protein M |
| ECA3101(ECAORF3063_1_sense) | 0.326729 | 0.001377 | 0.432357 | 0.012753 | outL | general secretion pathway protein L |
| ECA3105(ECAORF3067_1_sense) | 0.265303 | 0.03047 | 0.416683 | 0.001858 | outH | general secretion pathway protein H |
| ECA3106(ECAORF3068_1_sense) | 0.133681 | 0.009711 | 0.288476 | 0.009181 | outG | general secretion pathway protein G |
| ECA3107(ECAORF3069_1_sense) | 0.566733 | 0.007654 | 0.553422 | 0.003441 | outF | general secretion pathway protein F |
| ECA3109(ECAORF3071_1_sense) |  |  | 0.650423 | 0.031412 | outD | general secretion pathway protein D |
| ECA3114(ECAORF3076_1_sense) | 1.724142 | 0.006753 |  |  | outS | general secretion pathway lipoprotein |
| ECA3115(ECAORF3077_1_sense) | 0.479491 | 0.00155 |  |  | ECA3115 | putative aminotransferase |
| ECA3117(ECAORF2208_1_sense) |  |  | 0.249143 | 0.025525 | dat | D-alanine aminotransferase |
| ECA3117(ECAORF3079_1_sense) |  |  | 2.552733 | 0.012017 |  | PROBE05992 |
| ECA3118(ECAORF3080_1_sense) | 1.703147 | 0.003206 |  |  | ECA3118 | putative mechanosensitive ion channel protein |
| ECA3119(ECAORF3081_1_sense) | 2.142139 | 3.14E-04 |  |  | ECA3119 | hypothetical protein |
| ECA3123(ECAORF3085_1_sense) | 1.588097 | 4.65E-04 |  |  | ECA3123 | putative membrane protein |
| ECA3129(ECAORF3091_1_sense) | 0.545692 | 0.00228 | 0.591585 | 0.012239 | dltB | peptidoglycan biosynthesis protein |
| ECA3129(ECAORF3091_3_sense) | 0.622958 | 0.002895 | 0.450598 | 0.003866 | dltB | peptidoglycan biosynthesis protein |
| ECA3130(ECAORF3092_1_sense) | 0.522649 | 0.002361 |  |  | dltD | poly(glycerophosphate chain) D-alanine transfer protein |
| ECA3133(ECAORF3095_1_sense) | 0.53971 | 0.004883 |  |  | ECA3133 | hypothetical protein |
| ECA3150(ECAORF3112_2_sense) |  |  | 1.670687 | 0.008247 | ECA3150 | conserved hypothetical protein |
| ECA3156(ECAORF3117_1_sense) | 2.13475 | 0.008744 |  |  | purE | phosphoribosylaminoimidazole carboxylase catalytic subunit |
| ECA3157(ECAORF3118_1_sense) | 2.02788 | 0.006384 |  |  | purK | phosphoribosylaminoimidazole carboxylase ATPase subunit |
| ECA3157(ECAORF3118_3_sense) | 1.971966 | 0.012534 |  |  | purK | phosphoribosylaminoimidazole carboxylase ATPase subunit |
| ECA3164(ECAORF3125_1_sense) | 0.566536 | 0.020079 |  |  | ppk | polyphosphate kinase |
| ECA3167(ECAORF3128_1_sense) | 4.069994 | 0.006148 |  |  | ohr | organic hydroperoxide resistance protein |
| ECA3168(ECAORF3129_1_sense) | 2.504595 | 5.68E-04 | 4.221329 | 4.25E-05 | ohrR | transcriptional regulator of organic hydroperoxide resistance gene ohr |
| ECA3182(ECAORF3143_1_sense) | 0.41854 | 0.007658 |  |  | ECA3182 | probable oxidoreductase |
| ECA3184(ECAORF3145_1_sense) |  |  | 0.575658 | 0.046837 | ECA3184 | putative HlyD family secretion protein |
| ECA3191(ECAORF3152_3_sense) |  |  | 1.726503 | 0.00363 | ECA3191 | putative acetyltransferase |
| ECA3192(ECAORF3153_1_sense) | 5.671306 | 0.023632 |  |  | ECA3192 | conserved hypothetical protein |
| ECA3195(ECAORF3157_1_sense) | 0.42702 | 0.009084 |  |  | ECA3195 | putative membrane protein |
| ECA3204(ECAORF3166_1_sense) | 0.357894 | 2.15E-05 |  |  | ECA3204 | putative exported protein |
| ECA3204(ECAORF3166_3_sense) | 0.40482 | 0.010453 |  |  | ECA3204 | putative exported protein |
| ECA3205(ECAORF3167_1_sense) | 0.29188 | 0.00438 |  |  | ECA3205 | putative exported choloylglycine hydrolase |
| ECA3207(ECAORF3169_1_sense) | 1.580274 | 0.005794 |  |  | ECA3207 | conserved hypothetical protein |
| ECA3208(ECAORF3170_1_sense) | 2.216927 | 0.012446 |  |  | guaA | GMP synthase [glutamine-hydrolyzing] |
| ECA3209(ECAORF3171_1_sense) | 1.817656 | 0.017323 |  |  | guaB | inosine-5'-monophosphate dehydrogenase |
| ECA3216(ECAORF3178_1_sense) | 3.153454 | 0.004478 |  |  | engA | probable GTP-binding protein |
| ECA3217(ECAORF3179_1_sense) | 2.076839 | 0.034105 |  |  | ECA3217 | putative lipoprotein |
| ECA3218(ECAORF3180_1_sense) |  |  | 0.591953 | 0.010538 | ECA3218 | putative membrane protein |
| ECA3219(ECAORF3181_1_sense) | 1.483823 | 2.72E-04 |  |  | hisS | histidyl-tRNA synthetase |
| ECA3220(ECAORF3182_1_sense) | 1.68138 | 0.013989 |  |  | ispG | 1-hydroxy-2-methyl-2-(e)-butenyl 4-diphosphate synthase |
| ECA3222(ECAORF3184_1_sense) | 2.529965 | 0.008247 |  |  | ECA3222 | conserved hypothetical protein |
| ECA3223(ECAORF3185_1_sense) | 1.857842 | 0.001201 |  |  | ECA3223 | conserved hypothetical protein |
| ECA3237(ECAORF3199_1_sense) |  |  | 1.646355 | 0.049899 | iscS | cysteine desulfurase |
| ECA3238(ECAORF3200_1_sense) | 2.246782 | 0.036203 |  |  | ECA3238 | putative transcriptional regulator |
| ECA3239(ECAORF3201_1_sense) | 2.486415 | 6.02E-04 |  |  | ECA3239 | putative RNA methyltransferase |
| ECA3241(ECAORF3203_1_sense) | 2.665365 | 0.024006 |  |  | ECA3241 | ABC transporter, substrate binding protein |
| ECA3241(ECAORF3203_3_sense) | 4.336903 | 0.020916 |  |  | ECA3241 | ABC transporter, substrate binding protein |
| ECA3245(ECAORF3207_1_sense) | 0.548658 | 0.036778 |  |  | ECA3245 | methyl-accepting chemotaxis protein |
| ECA3251(ECAORF3213_1_sense) |  |  | 0.4349 | 0.037956 | hmpX | flavohemoprotein |
| ECA3254(ECAORF3216_1_sense) | 0.454161 | 3.73E-04 |  |  | glnB | nitrogen regulatory protein P-II |
| ECA3254(ECAORF3216_3_sense) | 0.484892 | 0.032003 |  |  | glnB | nitrogen regulatory protein P-II |
| ECA3262(ECAORF3224_1_sense) |  |  | 1.711354 | 0.006213 | ECA3262 | putative phophosugar-binding protein |
| ECA3263(ECAORF3225_1_sense) | 1.913762 | 0.01578 | 1.998647 | 0.041386 | ECA3263 | putative exported protein |
| ECA3266(ECAORF3228_1_sense) | 2.597786 | 0.006734 |  |  | ECA3266 | putative hemagglutinin/hemolysin-related protein |
| ECA3267(ECAORF3229_1_sense) |  |  | 0.430101 | 0.018423 | aggA | agglutination protein |
| ECA3270(ECAORF3232_1_sense) |  |  | 0.603285 | 0.030518 | ECA3270 | putative exported protein |
| ECA3272(ECAORF3234_1_sense) | 0.426465 | 0.016223 |  |  | ECA3272 | hypothetical protein |
| ECA3274(ECAORF3236_1_sense) |  |  | 1.39537 | 0.030369 | acpS | holo-[acyl-carrier protein] synthase |
| ECA3275(ECAORF3237_1_sense) | 0.551634 | 8.32E-05 |  |  | pdxJ | pyridoxal phosphate biosynthetic protein |
| ECA3276(ECAORF3238_1_sense) | 3.923571 | 0.033444 |  |  | recO | DNA repair protein |
| ECA3277(ECAORF3239_1_sense) | 2.378337 | 0.008122 |  |  | era | GTP-binding protein |
| ECA3278(ECAORF3240_1_sense) | 2.17795 | 0.020774 |  |  | rnc | ribonuclease III |
| ECA3279(ECAORF3241_1_sense) | 2.100807 | 0.042566 |  |  | lepB | signal peptidase I |
| ECA3280(ECAORF3242_1_sense) | 2.370633 | 0.026766 |  |  | lepA | GTP-binding protein |
| ECA3281(ECAORF3243_1_sense) | 2.30878 | 0.041686 |  |  | rseC | sigma-E factor regulatory protein |
| ECA3282(ECAORF3244_1_sense) |  |  | 1.743764 | 0.02721 | rseB | sigma-E factor regulatory protein |
| ECA3285(ECAORF3247_1_sense) |  |  | 1.925433 | 0.005233 | nadB | L-aspartate oxidase |
| ECA3300(ECAORF3262_1_sense) | 0.29095 | 0.004596 |  |  | cdaR | carbohydrate diacid regulator |
| ECA3300(ECAORF3262_3_sense) | 0.260802 | 0.01752 |  |  | cdaR | carbohydrate diacid regulator |
| ECA3301(ECAORF3263_1_sense) | 0.496014 | 0.026031 |  |  | degP | protease Do |
| ECA3303(ECAORF3265_1_sense) | 1.550931 | 0.002465 |  |  | mtn | Mta/Sah nucleosidase (P46) [includes: 5'-methylthioadenosine nucleosidase and S-adenosylhomocysteine nucleosidase] |
| ECA3312(ECAORF3274_1_sense) | 2.793416 | 1.31E-04 |  |  | fhuC | ferrichrome transport ATP-binding protein |
| ECA3315(ECAORF3277_1_sense) | 0.605881 | 0.049798 |  |  | ligT | 2'-5' RNA ligase |
| ECA3316(ECAORF3278_1_sense) | 0.273474 | 0.001406 |  |  | sfsA | sugar fermentation stimulation protein A |
| ECA3318(ECAORF3280_1_sense) | 2.280012 | 0.003461 |  |  | ECA3318 | putative glutamyl-tRNA synthetase |
| ECA3321(ECAORF3283_1_sense) | 1.710023 | 0.001128 |  |  | panB | 3-methyl-2-oxobutanoate hydroxymethyltransferase |
| ECA3322(ECAORF3284_1_sense) |  |  | 0.610073 | 0.013925 | panC | pantoate--beta-alanine ligase |
| ECA3330(ECAORF3292_1_sense) | 3.596733 | 0.030988 |  |  | ECA3330 | ATP transporter ATP-binding protein |
| ECA3343(ECAORF3305_1_sense) | 2.897542 | 0.044228 |  |  | mltD | membrane-bound lytic murein transglycosylase D precursor |
| ECA3346(ECAORF3308_1_sense) | 1.976613 | 0.020069 |  |  | ECA3346 | conserved hypothetical protein |
| ECA3347(ECAORF3309_1_sense) | 2.52056 | 2.60E-05 |  |  | rluD | ribosomal large subunit pseudouridine synthase D |
| ECA3348(ECAORF3310_1_sense) |  |  | 1.523863 | 0.043787 | ECA3348 | putative lipoprotein |
| ECA3349(ECAORF3311_1_sense) | 0.071298 | 0.001725 | 2.106156 | 0.007435 | ECA3349 | putative sigma(54) modulation protein |
| ECA3353(ECAORF3315_1_sense) |  |  | 1.687848 | 0.014968 | ECA3353 | putative two-component system response regulator |
| ECA3355(ECAORF3317_1_sense) | 2.221995 | 0.010868 | 2.017971 | 0.031345 | ECA3355 | conserved hypothetical protein |
| ECA3357(ECAORF3319_1_sense) | 4.093834 | 0.04955 |  |  | trmD | tRNA(guanine-N1)methyltransferase |
| ECA3358(ECAORF3320_1_sense) | 2.624723 | 0.047104 |  |  | rimM | 16S rRNA processing protein |
| ECA3360(ECAORF3322_1_sense) | 1.527202 | 0.01589 | 1.519524 | 0.031936 | ffh | signal recognition particle protein |
| ECA3362(ECAORF3324_1_sense) | 0.42682 | 0.005087 |  |  | luxS | autoinducer-2 production protein |
| ECA3366(ECAORF3328_1_sense) | 1.888043 | 0.004904 |  |  | csrA | carbon storage regulator |
| ECA3366(ECAORF3328_2_sense) | 1.820242 | 0.01428 |  |  | csrA | carbon storage regulator |
| ECA3366(ECAORF3328_3_sense) | 2.117902 | 0.010095 |  |  | csrA | carbon storage regulator |
| ECA3368(ECAORF3330_1_sense) | 2.092566 | 5.76E-04 |  |  | recX | regulatory protein Recx |
| ECA3378(ECAORF3340_1_sense) | 0.42518 | 0.024455 |  |  | ECA3378 | putative bacteriocin immunity protein |
| ECA3379(ECAORF3341_1_sense) | 0.486006 | 0.047611 |  |  | ECA3379 | putative glycosyltransferase |
| ECA3380(ECAORF3342_1_sense) | 0.063844 | 7.55E-04 | 0.042789 | 0.004554 | pvcB | pyoverdine biosynthesis protein |
| ECA3381(ECAORF3343_1_sense) | 0.234161 | 0.035695 |  |  | pvcA | pyoverdine biosynthesis protein |
| ECA3383(ECAORF3347_1_sense) |  |  | 0.643736 | 0.003 | ECA3383 | conserved hypothetical protein |
| ECA3384(ECAORF3348_1_sense) | 0.218707 | 0.003903 |  |  | ECA3384 | hypothetical protein |
| ECA3385(ECAORF3349_1_sense) | 0.437931 | 5.98E-06 |  |  | ECA3385 | hypothetical protein |
| ECA3385(ECAORF3349_2_sense) | 0.524633 | 0.035596 |  |  | ECA3385 | hypothetical protein |
| ECA3392(ECAORF3356_1_sense) | 1.605852 | 0.003246 |  |  | ECA3392 | conserved hypothetical protein |
| ECA3393(ECAORF3357_2_sense) |  |  | 1.432668 | 0.044685 | ECA3393 | conserved hypothetical protein |
| ECA3394(ECAORF3358_1_sense) | 0.209683 | 3.14E-04 |  |  | ECA3394 | hypothetical protein |
| ECA3395(ECAORF3359_1_sense) | 0.344203 | 0.003051 |  |  | ECA3395 | hypothetical protein |
| ECA3395(ECAORF3359_3_sense) | 0.382487 | 0.020049 |  |  | ECA3395 | hypothetical protein |
| ECA3401(ECAORF3365_1_sense) | 1.567357 | 0.033366 |  |  | ECA3401 | putative phage regulatory protein |
| ECA3402(ECAORF3366_1_sense) | 2.254713 | 0.017825 |  |  | ECA3402 | conserved hypothetical protein |
| ECA3405(ECAORF3369_1_sense) | 2.18381 | 0.002322 |  |  | ECA3405 | hypothetical protein |
| ECA3408(ECAORF3372_1_sense) | 1.532772 | 0.027673 |  |  | ECA3408 | putative phage regulatory protein |
| ECA3408(ECAORF3372_2_sense) | 1.989478 | 0.019484 |  |  | ECA3408 | putative phage regulatory protein |
| ECA3412(ECAORF3376_1_sense) | 0.63261 | 0.027787 |  |  | ECA3412 | hypothetical protein |
| ECA3414(ECAORF3378_1_sense) | 1.878895 | 0.030249 |  |  | ECA3414 | hypothetical protein |
| ECA3417(ECAORF3381_3_sense) | 1.561594 | 0.021322 |  |  | ECA3417 | putative phage regulatory protein |
| ECA3420(ECAORF3384_1_sense) | 0.126517 | 0.001367 |  |  | ECA3420 | conserved hypothetical protein |
| ECA3421(ECAORF3385_1_sense) | 0.093614 | 0.00377 | 0.186547 | 0.010157 | ECA3421 | Rhs protein |
| ECA3423(ECAORF3387_1_sense) | 0.211683 | 0.005298 | 0.209516 | 0.012483 | ECA3423 | hypothetical protein |
| ECA3424(ECAORF3388_1_sense) | 0.440734 | 0.004182 | 0.363846 | 0.003742 | ECA3424 | putative lipoprotein |
| ECA3425(ECAORF3389_1_sense) | 0.356119 | 0.001707 | 0.384247 | 0.007093 | ECA3425 | putative lipoprotein |
| ECA3426(ECAORF3390_1_sense) | 0.317414 | 0.01866 | 0.326032 | 0.01208 | ECA3426 | putative phospholipase |
| ECA3426(ECAORF3390_3_sense) | 0.250498 | 0.004862 | 0.222637 | 0.013863 | ECA3426 | putative phospholipase |
| ECA3427(ECAORF3391_1_sense) | 0.213777 | 0.001525 |  |  | ECA3427 | VgrG-like protein |
| ECA3430(ECAORF3393_1_sense) | 0.261528 | 0.014731 |  |  | ECA3430 | putative lipoprotein |
| ECA3431(ECAORF3394_1_sense) | 0.137896 | 2.90E-04 | 0.326518 | 0.001489 | ECA3431 | putative membrane protein |
| ECA3432(ECAORF3395_1_sense) | 0.236708 | 0.001661 | 0.386097 | 0.024915 | *vasK* | putative virulence-associated protein |
| ECA3433(ECAORF3396_1_sense) | 0.379778 | 0.030813 |  |  | ECA3433 | conserved hypothetical protein |
| ECA3436(ECAORF3399_1_sense) | 0.226273 | 0.018832 | 0.306898 | 0.022647 | *vasG* | putative chaperone |
| ECA3440(ECAORF3403_1_sense) | 0.093697 | 1.31E-04 | 0.180265 | 0.016231 | ECA3440 | conserved hypothetical protein |
| ECA3442(ECAORF3405_1_sense) | 0.265307 | 0.007934 | 0.235062 | 0.014964 | *vasA* | conserved hypothetical protein |
| ECA3443(ECAORF3406_1_sense) | 0.20377 | 0.043396 | 0.291824 | 0.016367 | ECA3443 | conserved hypothetical protein |
| ECA3444(ECAORF3407_1_sense) | 0.012153 | 8.17E-05 | 0.022644 | 4.66E-04 | ECA3444 | conserved hypothetical protein |
| ECA3445(ECAORF3408_1_sense) | 0.397761 | 0.033408 |  |  | ECA3445 | conserved hypothetical protein |
| ECA3455(ECAORF3418_1_sense) | 0.368031 | 1.92E-05 |  |  | ECA3455 | conserved hypothetical protein |
| ECA3455(ECAORF3418_2_sense) | 0.402694 | 0.020672 |  |  | ECA3455 | conserved hypothetical protein |
| ECA3455(ECAORF3418_3_sense) | 0.42725 | 0.001462 |  |  | ECA3455 | conserved hypothetical protein |
| ECA3456(ECAORF3419_1_sense) | 0.341416 | 0.004905 |  |  | ECA3456 | hypothetical protein |
| ECA3457(ECAORF3420_1_sense) | 0.493526 | 0.003919 | 0.468011 | 0.019234 | ECA3457 | putative membrane protein |
| ECA3458(ECAORF3421_1_sense) | 0.612697 | 0.028399 | 0.575035 | 0.039167 | ECA3458 | conserved hypothetical protein |
| ECA3458(ECAORF3421_3_sense) | 0.601358 | 0.044878 |  |  | ECA3458 | conserved hypothetical protein |
| ECA3459(ECAORF3422_1_sense) | 0.369872 | 0.035326 |  |  | ECA3459 | conserved hypothetical protein |
| ECA3462(ECAORF3425_1_sense) |  |  | 1.450193 | 0.013038 | proA | gamma-glutamyl phosphate reductase |
| ECA3464(ECAORF3427_1_sense) | 0.483837 | 4.74E-04 |  |  | crl | curlin genes transcriptional activator |
| ECA3477(ECAORF3440_1_sense) | 1.639648 | 0.003707 |  |  | ECA3477 | putative translation initiation factor EIF-2B |
| ECA3480(ECAORF3443_1_sense) | 0.491981 | 0.007043 |  |  | ECA3480 | conserved hypothetical protein |
| ECA3481(ECAORF3444_1_sense) | 0.565558 | 0.001765 |  |  | ECA3481 | putative membrane protein |
| ECA3509(ECAORF3470_1_sense) | 1.534894 | 0.022674 |  |  | ECA3509 | putative amino acid transporter |
| ECA3510(ECAORF3471_1_sense) | 1.884876 | 0.02412 |  |  | ECA3510 | putative membrane protein |
| ECA3512(ECAORF3473_1_sense) | 2.054468 | 0.029361 | 2.442239 | 0.004502 | emrA | multidrug resistance protein A |
| ECA3513(ECAORF3474_1_sense) | 4.049681 | 0.036898 |  |  | emrB | multidrug resistance protein B |
| ECA3515(ECAORF3476_1_sense) | 2.167341 | 0.027592 |  |  | ECA3515 | putative lipoprotein |
| ECA3518(ECAORF3479_1_sense) | 2.318315 | 0.005272 |  |  | ECA3518 | conserved hypothetical protein |
| ECA3526(ECAORF3487_1_sense) | 0.521943 | 0.004582 |  |  | metQ | D-methionine-binding lipoprotein |
| ECA3528(ECAORF3489_1_sense) | 2.71999 | 0.048459 |  |  | ECA3528 | conserved hypothetical protein |
| ECA3535(ECAORF3496_1_sense) | 2.589034 | 0.003898 |  |  | ispD | 2-C-methyl-D-erythritol 4-phosphate cytidylyltransferase |
| ECA3536(ECAORF3497_1_sense) | 1.477447 | 0.046885 |  |  | ftsB | cell division protein |
| ECA3546(ECAORF3507_1_sense) | 0.391438 | 5.45E-04 |  |  | cysI | sulfite reductase [NADPH] hemoprotein beta-component |
| ECA3547(ECAORF3508_1_sense) | 0.315592 | 0.014304 |  |  | cysJ | sulfite reductase [NADPH] flavoprotein alpha-component |
| ECA3548(ECAORF3509_1_sense) |  |  | 0.609712 | 0.003147 | ECA3548 | conserved hypothetical protein |
| ECA3554(ECAORF3515_1_sense) | 2.090737 | 8.62E-05 |  |  | ECA3554 | conserved hypothetical protein |
| ECA3554(ECAORF3515_3_sense) |  |  | 2.317787 | 0.045489 | ECA3554 | conserved hypothetical protein |
| ECA3562(ECAORF3523_1_sense) |  |  | 5.458466 | 0.041565 | calB | coniferyl aldehyde dehydrogenase |
| ECA3564(ECAORF3525_1_sense) | 1.936528 | 0.033675 |  |  | ECA3564 | iron-chelating periplasmic-binding protein |
| ECA3567(ECAORF3528_1_sense) | 2.31696 | 0.025242 |  |  | pyrG | CTP synthase |
| ECA3568(ECAORF3529_1_sense) | 2.107247 | 0.008794 |  |  | mazG | conserved hypothetical protein |
| ECA3568(ECAORF3529_3_sense) | 2.396201 | 0.007163 |  |  | mazG | conserved hypothetical protein |
| ECA3573(ECAORF3534_1_sense) | 0.476163 | 0.017748 |  |  | garR | 2-hydroxy-3-oxopropionate reductase |
| ECA3574(ECAORF3535_1_sense) | 1.590809 | 0.033045 |  |  | garL | 2-dehydro-3-deoxyglucarate aldolase |
| ECA3581(ECAORF3542_1_sense) | 0.194815 | 0.001047 |  |  | ECA3581 | putative exported protein |
| ECA3582(ECAORF3543_1_sense) | 2.145688 | 0.030795 | 2.207761 | 0.010207 | ECA3582 | conserved hypothetical protein |
| ECA3584(ECAORF3545_1_sense) | 1.791555 | 0.032186 |  |  | rfaE | ADP-heptose synthase |
| ECA3589(ECAORF3550_1_sense) | 1.846974 | 1.12E-04 |  |  | bacA | bacitracin resistance protein |
| ECA3590(ECAORF3551_1_sense) |  |  | 2.923222 | 0.006748 | folB | dihydroneopterin aldolase |
| ECA3591(ECAORF3552_1_sense) |  |  | 2.115965 | 0.007874 | ECA3591 | putative membrane protein |
| ECA3592(ECAORF3553_1_sense) | 1.607172 | 8.13E-04 |  |  | ECA3592 | methyl-accepting chemotaxis protein |
| ECA3598(ECAORF3559_1_sense) | 2.283666 | 0.02176 |  |  | ECA3598 | putative membrane protein |
| ECA3607(ECAORF3568_1_sense) |  |  | 0.639441 | 0.042159 | fklB | FkbP-type peptidyl-prolyl cis-trans isomerase |
| ECA3608(ECAORF3569_1_sense) | 1.597864 | 7.92E-04 |  |  | ECA3608 | conserved hypothetical protein |
| ECA3610(ECAORF3571_1_sense) | 2.720432 | 0.020639 |  |  | rplI | 50S ribosomal protein L9 |
| ECA3611(ECAORF3572_1_sense) |  |  | 0.466122 | 0.006571 | rpsR | 30S ribosomal protein S18 |
| ECA3612(ECAORF3573_1_sense) |  |  | 0.430946 | 0.023336 | priB | primosomal replication protein N |
| ECA3616(ECAORF3577_1_sense) | 1.966967 | 0.038225 |  |  | ECA3616 | conserved hypothetical protein |
| ECA3620(ECAORF3581_1_sense) | 2.426121 | 0.009149 |  |  | ECA3620 | putative membrane protein |
| ECA3621(ECAORF3582_1_sense) | 1.920963 | 9.11E-04 | 2.012024 | 0.006229 | ECA3621 | probable tRNA/rRNA methyltransferase |
| ECA3622(ECAORF3583_1_sense) | 2.159121 | 0.001083 |  |  | rnr | ribonuclease R |
| ECA3623(ECAORF3584_1_sense) | 1.421021 | 0.03053 |  |  | ECA3623 | conserved hypothetical protein |
| ECA3631(ECAORF3592_1_sense) | 1.721955 | 0.017248 |  |  | ECA3631 | Ham1 protein homolog |
| ECA3631(ECAORF3592_2_sense) | 2.987733 | 0.040669 |  |  | ECA3631 | Ham1 protein homolog |
| ECA3636(ECAORF3597_1_sense) | 3.933852 | 3.96E-04 |  |  | ECA3636 | putative exported protein |
| ECA3638(ECAORF3599_3_sense) |  |  | 2.474544 | 0.049389 | ecnA | entericidin A precursor |
| ECA3655(ECAORF3616_1_sense) | 0.516697 | 0.044388 |  |  | ECA3655 | putative exported protein |
| ECA3655(ECAORF3616_3_sense) | 0.425523 | 0.025776 |  |  | ECA3655 | putative exported protein |
| ECA3657(ECAORF3618_1_sense) | 0.309279 | 0.025272 |  |  | ECA3657 | putative exported protein |
| ECA3659(ECAORF3620_1_sense) | 0.372962 | 0.03648 |  |  | ECA3659 | conserved hypothetical protein |
| ECA3663(ECAORF3624_1_sense) |  |  | 2.324843 | 0.037858 | qor | quinone oxidoreductase |
| ECA3672(ECAORF3633_1_sense) | 0.145285 | 1.09E-04 | 0.245755 | 0.045206 | ECA3672 | HcpA homologue |
| ECA3673(ECAORF3634_1_sense) | 0.269261 | 3.63E-04 |  |  | ECA3673 | putative membrane protein |
| ECA3674(ECAORF3635_1_sense) | 0.206413 | 1.37E-04 | 0.279755 | 0.030414 | ECA3674 | putative membrane protein |
| ECA3674(ECAORF3635_3_sense) | 0.221291 | 0.013011 | 0.246396 | 0.035778 | ECA3674 | putative membrane protein |
| ECA3675(ECAORF3636_1_sense) | 0.155561 | 0.00267 | 0.264424 | 0.02705 | ECA3675 | putative membrane protein |
| ECA3688(ECAORF3649_1_sense) | 2.315307 | 0.005567 |  |  | ECA3688 | conserved hypothetical protein |
| ECA3689(ECAORF3650_1_sense) | 2.123143 | 0.027909 |  |  | uvrA | excision nuclease subunit A |
| ECA3692(ECAORF3653_1_sense) | 0.595295 | 0.016226 |  |  | ECA3692 | probable acetyltransferase |
| ECA3695(ECAORF3656_3_sense) | 0.516704 | 0.04492 |  |  | ECA3695 | phage regulatory protein protein |
| ECA3699(ECAORF3660_1_sense) | 0.423506 | 0.017685 |  |  | ECA3699 | conserved hypothetical phage-related protein |
| ECA3709(ECAORF3670_1_sense) | 0.497568 | 0.013547 |  |  | ECA3709 | putative phage-related membrane protein |
| ECA3710(ECAORF3671_1_sense) | 0.400238 | 4.39E-04 | 0.395489 | 0.037846 | ECA3710 | putative phage-related lipoprotein |
| ECA3714(ECAORF3675_1_sense) | 1.650971 | 0.001037 | 0.469385 | 2.54E-04 | ECA3714 | putative phage-related lytic transglycosylase |
| ECA3717(ECAORF3678_1_sense) | 1.451217 | 0.049292 | 0.395893 | 0.005491 | ECA3717 | conserved hypothetical phage-related protein |
| ECA3718(ECAORF3679_1_sense) | 3.487254 | 0.009379 |  |  | ECA3718 | conserved hypothetical phage-related protein |
| ECA3725(ECAORF3686_1_sense) | 3.367266 | 0.048543 |  |  | ECA3725 | putative phage-related exported protein |
| ECA3728(ECAORF3689_1_sense) | 1.823022 | 0.007914 |  |  | ECA3728 | conserved phage-related protein |
| ECA3739(ECAORF3701_1_sense) | 1.569695 | 0.002568 |  |  | ECA3739 | putative bacteriophage baseplate assembly protein |
| ECA3751(ECAORF3713_1_sense) | 0.286597 | 0.011918 |  |  | ECA3751 | extracellular solute-binding protein |
| ECA3776(ECAORF3738_1_sense) | 0.580061 | 0.0379 |  |  | fsaB | fructose-6-phosphate aldolase |
| ECA3777(ECAORF3739_1_sense) | 0.555502 | 0.047506 |  |  | ECA3777 | conserved hypothetical protein |
| ECA3779(ECAORF3741_1_sense) |  |  | 0.445854 | 0.037439 | ECA3779 | putative exported protein |
| ECA3782(ECAORF3744_1_sense) |  |  | 1.893871 | 0.006973 | ECA3782 | hypothetical protein |
| ECA3783(ECAORF3745_1_sense) |  |  | 2.687206 | 0.043905 | ECA3783 | AraC-family transcriptional regulator |
| ECA3784(ECAORF3746_1_sense) | 0.482061 | 0.045051 |  |  | ECA3784 | conserved hypothetical protein |
| ECA3791(ECAORF2388_1_sense) | 2.853852 | 8.49E-04 | 2.450773 | 0.041688 | aroP | aromatic amino acid transport protein |
| ECA3801(ECAORF3763_1_sense) | 0.445148 | 0.001471 |  |  | guaC | GMP reductase |
| ECA3805(ECAORF3767_1_sense) | 1.597359 | 0.001316 | 1.828774 | 0.025942 | mutT | mutator protein (7,8-dihydro-8-oxoguanine-triphosphatase) |
| ECA3805(ECAORF3767_3_sense) | 1.870169 | 0.044582 | 1.890763 | 0.003324 | mutT | mutator protein (7,8-dihydro-8-oxoguanine-triphosphatase) |
| ECA3807(ECAORF3769_1_sense) | 2.431946 | 0.041094 |  |  | secM | secretion monitor precursor |
| ECA3810(ECAORF3772_1_sense) | 1.507227 | 0.043831 |  |  | ftsZ | cell division protein |
| ECA3811(ECAORF3773_1_sense) | 1.926609 | 0.014526 |  |  | ftsA | cell division protein |
| ECA3812(ECAORF3774_1_sense) | 2.018389 | 0.013386 |  |  | ftsQ | cell division protein |
| ECA3813(ECAORF3775_1_sense) | 1.957732 | 0.014221 |  |  | ddlB | D-alanine--D-alanine ligase B |
| ECA3814(ECAORF3776_1_sense) | 2.087355 | 0.002994 |  |  | murC | UDP-N-acetylmuramate--L-alanine ligase |
| ECA3815(ECAORF3777_1_sense) | 1.566862 | 0.001957 |  |  | murG | UDP-N-acetylglucosamine--N-acetylmuramyl- (pentape ptide) pyrophosphoryl-undecaprenol N-acetylglucosamine transferase |
| ECA3816(ECAORF3778_1_sense) | 2.646239 | 0.007166 |  |  | ftsW | cell division protein |
| ECA3818(ECAORF3780_1_sense) | 2.014845 | 7.27E-04 |  |  | mraY | phospho-N-acetylmuramoyl-pentapeptide- transferase |
| ECA3831(ECAORF3792_1_sense) | 1.482397 | 0.029046 |  |  | leuA | 2-isopropylmalate synthase |
| ECA3834(ECAORF3795_1_sense) | 0.667582 | 0.01069 |  |  | leuD | 3-isopropylmalate dehydratase small subunit |
| ECA3839(ECAORF3800_1_sense) |  |  | 2.519351 | 0.022308 | ECA3839 | putative matrix protein |
| ECA3844(ECAORF3805_1_sense) | 1.75091 | 0.019011 |  |  | tbpA | thiamine-binding periplasmic protein precursor |
| ECA3854(ECAORF3815_1_sense) | 1.869381 | 0.030784 |  |  | rluA | ribosomal large subunit pseudouridine synthase A |
| ECA3860(ECAORF3821_1_sense) | 2.112917 | 0.005812 |  |  | apaG | conserved hypothetical protein |
| ECA3863(ECAORF3824_1_sense) | 2.039077 | 0.032421 |  |  | ECA3863 | LysR-family transcriptional regulator |
| ECA3873(ECAORF3834_1_sense) | 2.234105 | 0.022024 |  |  | ispH | IspH protein (1-hydroxy-2-methyl-2-(E)-butenyl 4-diphosphate reductase) |
| ECA3875(ECAORF3836_1_sense) | 2.530456 | 0.017509 |  |  | lspA | lipoprotein signal peptidase |
| ECA3876(ECAORF3837_1_sense) | 2.109048 | 0.003752 |  |  | ileS | isoleucyl-tRNA synthetase |
| ECA3881(ECAORF3842_1_sense) |  |  | 2.338774 | 0.045434 | dnaJ | chaperone protein DnaJ |
| ECA3887(ECAORF3848_1_sense) |  |  | 1.464605 | 0.04283 | talB | transaldolase B |
| ECA3893(ECAORF3854_1_sense) |  |  | 0.629648 | 0.041478 | arcA | aerobic respiration control protein |
| ECA3894(ECAORF3855_1_sense) | 0.417788 | 0.039756 |  |  | creA | putative exported protein |
| ECA3895(ECAORF3856_1_sense) | 1.76163 | 0.041315 |  |  | ECA3895 | hypothetical protein |
| ECA3904(ECAORF3865_1_sense) | 1.726214 | 0.003042 |  |  | ECA3904 | putative zinc-binding dehydrogenase |
| ECA3913(ECAORF3874_1_sense) |  |  | 2.199048 | 0.029433 | epd | D-erythrose 4-phosphate dehydrogenase |
| ECA3915(ECAORF3876_1_sense) | 0.249023 | 0.025744 | 0.405725 | 0.003039 | ECA3915 | putative peptidase |
| ECA3917(ECAORF3877_1_sense) | 2.18993 | 0.024113 |  |  | ECA3917 | LuxR-family transcriptional regulator |
| ECA3924(ECAORF3884_1_sense) |  |  | 0.597218 | 0.046214 | gshB | glutathione synthetase |
| ECA3928(ECAORF3888_1_sense) |  |  | 2.807794 | 0.046051 | mpl | udp-N-acetylmuramate:L-alanyl-gamma-D-glutamyl- me so-diaminopimelate ligase |
| ECA3931(ECAORF3891_1_sense) | 2.695259 | 0.024799 |  |  | hflC | putative phage-related protein |
| ECA3933(ECAORF3893_1_sense) | 1.779914 | 0.024351 |  |  | hflX | putative GTP-binding phage-related protein |
| ECA3941(ECAORF3901_1_sense) | 0.623804 | 0.008483 |  |  | ahpF | alkyl hydroperoxide reductase subunit F |
| ECA3942(ECAORF3902_1_sense) | 0.266841 | 0.018681 |  |  | ahpC | alkyl hydroperoxide reductase C22 protein |
| ECA3945(ECAORF3905_1_sense) | 0.250582 | 0.009926 |  |  | ECA3945 | hypothetical protein |
| ECA3946(ECAORF3906_1_sense) | 0.217446 | 0.008052 |  |  | ECA3946 | putative exported protein |
| ECA3946(ECAORF3906_3_sense) |  |  | 0.505701 | 0.020739 | ECA3946 | putative exported protein |
| ECA3952(ECAORF3912_1_sense) | 0.486676 | 0.029935 |  |  | ECA3952 | LysR-family transcriptional regulator |
| ECA3957(ECAORF3917_1_sense) | 1.479331 | 0.007939 |  |  | ECA3957 | putative hydrolase |
| ECA3962(ECAORF3922_1_sense) | 0.393392 | 8.12E-04 |  |  | ECA3962 | putative exported protein |
| ECA3964(ECAORF3924_1_sense) |  |  | 2.270826 | 0.001736 | orn | oligoribonuclease |
| ECA3966(ECAORF3926_1_sense) | 1.802198 | 0.007436 |  |  | psd | phosphatidylserine decarboxylase proenzyme |
| ECA3968(ECAORF3928_1_sense) | 1.697906 | 0.003383 |  |  | poxA | putative lysyl-tRNA synthetase |
| ECA3969(ECAORF3929_1_sense) |  |  | 6.239788 | 0.033745 | frdA | fumarate reductase flavoprotein subunit |
| ECA3977(ECAORF3937_1_sense) | 2.191383 | 0.009799 |  |  | ECA3977 | conserved hypothetical protein |
| ECA3978(ECAORF3938_1_sense) |  |  | 1.997884 | 0.047756 | ECA3978 | putative lipoprotein |
| ECA3989(ECAORF3949_1_sense) |  |  | 1.374162 | 0.03519 | aceK | isocitrate dehydrogenase kinase/phosphatase |
| ECA3993(ECAORF3953_1_sense) | 0.550212 | 0.008684 | 1.580288 | 0.016134 | ECA3993 | putative transferase |
| ECA3997(ECAORF3957_1_sense) |  |  | 1.939536 | 7.70E-04 | smg | conserved hypothetical protein |
| ECA3999(ECAORF3959_1_sense) | 1.99887 | 7.90E-04 |  |  | def | peptide deformylase |
| ECA399A(ECAORF0392_2_sense) | 1.540583 | 0.005188 |  |  | ECA399A | None |
| ECA4001(ECAORF3961_1_sense) | 2.367078 | 0.005656 |  |  | sun | Sun protein |
| ECA4005(ECAORF3965_1_sense) | 1.996545 | 0.004099 |  |  | rplQ | 50S ribosomal protein L17 |
| ECA4008(ECAORF3968_1_sense) |  |  | 0.463289 | 0.040082 | rpsK | 30S ribosomal subunit protein S11 |
| ECA4011(ECAORF3971_1_sense) | 3.712278 | 0.001055 |  |  | secY | preprotein translocase subunit |
| ECA4014(ECAORF3974_1_sense) | 3.102923 | 0.020637 |  |  | rpsE | 30S ribosomal subunit protein S5 |
| ECA4016(ECAORF3976_1_sense) | 3.761217 | 0.039348 |  |  | rplF | 50S ribosomal subunit protein L6 |
| ECA4017(ECAORF3977_1_sense) | 3.089697 | 0.043356 | 0.661755 | 2.48E-04 | rpsH | 30S ribosomal subunit protein S8 |
| ECA4018(ECAORF3978_1_sense) | 3.321002 | 0.00349 |  |  | rpsN | 30S ribosomal subunit protein S14 |
| ECA4018(ECAORF3978_3_sense) | 3.383066 | 0.023697 |  |  | rpsN | 30S ribosomal subunit protein S14 |
| ECA4019(ECAORF3979_1_sense) | 4.131367 | 0.032365 |  |  | rplE | 50S ribosomal subunit protein L5 |
| ECA4020(ECAORF3980_1_sense) |  |  | 0.545266 | 0.017737 | rplX | 50S ribosomal subunit protein L24 |
| ECA4024(ECAORF3984_1_sense) |  |  | 0.545124 | 0.002915 | rplP | 50S ribosomal subunit protein L16 |
| ECA4028(ECAORF3988_1_sense) | 2.751074 | 0.028036 |  |  | rplB | 50S ribosomal subunit protein L2 |
| ECA4029(ECAORF3989_1_sense) | 3.218755 | 0.013256 |  |  | rplW | 50S ribosomal subunit protein L23 |
| ECA4030(ECAORF3990_1_sense) | 4.013767 | 0.045164 |  |  | rplD | 50S ribosomal subunit protein L4 |
| ECA4031(ECAORF3991_1_sense) | 3.460797 | 0.025851 |  |  | rplC | 50S ribosomal subunit protein L3 |
| ECA4034(ECAORF3994_1_sense) | 2.434523 | 0.039846 |  |  | bfd | bacterioferritin-associated ferredoxin |
| ECA4035(ECAORF3995_1_sense) |  |  | 0.443576 | 0.023229 | tufB | elongation factor Tu |
| ECA4037(ECAORF3997_1_sense) | 3.264128 | 7.43E-04 |  |  | rpsG | 30S ribosomal subunit protein S7 |
| ECA4038(ECAORF3998_1_sense) | 2.707593 | 0.00498 |  |  | rpsL | 30S ribosomal subunit protein S12 |
| ECA4040(ECAORF4000_1_sense) | 1.801867 | 0.033922 | 1.484189 | 0.021819 | dsrF | putative intracellular sulfur oxidation protein |
| ECA4041(ECAORF4001_1_sense) | 1.981368 | 0.013832 | 1.648809 | 0.004227 | dsrE | putative intracellular sulfur oxidation protein |
| ECA4050(ECAORF4010_1_sense) | 1.672938 | 0.026079 |  |  | slyX | conserved hypothetical protein |
| ECA4063(ECAORF4023_1_sense) | 0.377984 | 0.026331 |  |  | ECA4063 | conserved hypothetical protein |
| ECA4065(ECAORF4025_1_sense) | 0.473924 | 0.029642 |  |  | argD | acetylornithine/succinyldiaminopimelate aminotransferase |
| ECA4065(ECAORF4025_3_sense) | 0.461287 | 0.019086 |  |  | argD | acetylornithine/succinyldiaminopimelate aminotransferase |
| ECA4067(ECAORF4027_1_sense) | 0.063126 | 0.008391 | 0.079998 | 0.001709 | pelA | pectate lyase I |
| ECA4068(ECAORF4028_1_sense) | 0.115727 | 0.043331 |  |  | pelB | pectate lyase II |
| ECA4069(ECAORF4029_1_sense) |  |  | 0.228863 | 0.016611 | pelC | pectate lyase III |
| ECA4070(ECAORF4030_1_sense) | 0.24254 | 0.005807 | 0.159759 | 0.017533 | pelZ | pectate lyase |
| ECA4079(ECAORF4039_1_sense) | 0.179648 | 0.023931 | 0.202117 | 0.007799 | nirB | nitrite reductase [NAD(P)H] large subunit |
| ECA4080(ECAORF4040_1_sense) | 0.183657 | 0.004065 |  |  | nirD | nitrite reductase [NAD(P)H] small subunit |
| ECA4089(ECAORF4049_1_sense) | 1.593333 | 0.015175 |  |  | rpe | ribulose-phosphate 3-epimerase |
| ECA4091(ECAORF4051_1_sense) | 2.882557 | 0.019677 |  |  | damX | DamX protein |
| ECA4092(ECAORF4052_1_sense) | 4.439645 | 2.96E-05 |  |  | aroB | 3-dehydroquinate synthase |
| ECA4092(ECAORF4052_3_sense) | 4.042978 | 0.019323 |  |  | aroB | 3-dehydroquinate synthase |
| ECA4093(ECAORF4053_1_sense) | 2.563956 | 0.022187 |  |  | aroK | shikimate kinase I |
| ECA4099(ECAORF4059_1_sense) | 1.85594 | 0.042243 |  |  | mrcA | penicillin-binding protein 1A |
| ECA4104(ECAORF4064_1_sense) | 1.50731 | 0.001796 |  |  | hslR | heat shock protein |
| ECA4105(ECAORF4065_1_sense) | 1.881174 | 0.045028 |  |  | hslO | heat shock protein (33 kDa chaperonin) |
| ECA4107(ECAORF4067_1_sense) | 1.689893 | 0.007928 |  |  | envZ | two-component osmolarity sensor protein |
| ECA4110(ECAORF4070_1_sense) | 0.53262 | 0.011644 |  |  | ECA4110 | putative periplasmic substrate-binding protein |
| ECA4113(ECAORF4073_1_sense) | 0.116619 | 0.031423 | 0.283988 | 0.013586 | ECA4113 | putative cystathionine beta-synthase |
| ECA4118(ECAORF4078_1_sense) | 0.612677 | 0.007165 |  |  | greB | transcription elongation factor |
| ECA4120(ECAORF4080_1_sense) | 0.467828 | 0.01304 |  |  | ECA4120 | methyl-accepting chemotaxis protein |
| ECA4123(ECAORF4083_1_sense) | 0.303576 | 0.005177 |  |  | rexZ | regulator of exoenzymes |
| ECA4135(ECAORF4095_1_sense) | 1.626494 | 0.004343 |  |  | malQ | 4-alpha-glucanotransferase |
| ECA4137(ECAORF4097_1_sense) |  |  | 2.247371 | 7.75E-04 | glpR | glycerol-3-phosphate regulon repressor |
| ECA4139(ECAORF4099_1_sense) | 1.53763 | 0.024455 |  |  | glpE | thiosulfate sulfurtransferase |
| ECA4145(ECAORF4105_1_sense) | 0.396999 | 0.005754 |  |  | ECA4145 | conserved hypothetical protein |
| ECA4151(ECAORF4111_1_sense) | 0.252069 | 0.002097 |  |  | glgB | 1,4-alpha-glucan branching enzyme |
| ECA4152(ECAORF4112_1_sense) |  |  | 2.030036 | 0.007821 | ECA4152 | putative two-component system sensor kinase |
| ECA4152(ECAORF4112_3_sense) |  |  | 2.543859 | 0.017511 | ECA4152 | putative two-component system sensor kinase |
| ECA4157(ECAORF4117_1_sense) | 1.81465 | 0.006786 |  |  | ECA4157 | putative membrane protein |
| ECA4160(ECAORF1804_1_sense) | 2.543964 | 0.04181 |  |  | gntR | gluconate utilization system Gnt-I transcriptional repressor (partial) |
| ECA4162(ECAORF4122_1_sense) | 2.516226 | 0.043577 | 2.295208 | 0.01502 | ECA4162 | conserved hypothetical protein |
| ECA4163(ECAORF4123_1_sense) | 0.358873 | 0.001565 |  |  | glpC | anaerobic glycerol-3-phosphate dehydrogenase subunit C |
| ECA4164(ECAORF4124_1_sense) | 0.5064 | 0.045983 |  |  | glpB | anaerobic glycerol-3-phosphate dehydrogenase subunit B |
| ECA4165(ECAORF4125_1_sense) | 0.543136 | 0.014676 |  |  | glpA | anaerobic glycerol-3-phosphate dehydrogenase subunit A |
| ECA4167(ECAORF4127_1_sense) | 1.51957 | 0.017966 |  |  | glpQ | glycerophosphoryl diester phosphodiesterase, periplasmic precursor |
| ECA4168(ECAORF4128_1_sense) | 1.758611 | 0.010204 |  |  | ECA4168 | conserved hypothetical protein |
| ECA4175(ECAORF4135_1_sense) | 2.295135 | 3.41E-04 |  |  | ECA4175 | putative membrane protein |
| ECA4177(ECAORF4137_1_sense) | 1.809901 | 0.004254 |  |  | corA | magnesium and cobalt transport protein |
| ECA4185(ECAORF4145_1_sense) |  |  | 1.837077 | 0.0374 | cyaY | CyaY protein |
| ECA4186(ECAORF4146_1_sense) |  |  | 2.012241 | 0.00774 | ECA4186 | putative membrane protein |
| ECA4187(ECAORF4147_1_sense) |  |  | 2.683647 | 7.86E-04 | cyaA | adenylate cyclase |
| ECA4193(ECAORF4153_3_sense) |  |  | 1.612561 | 0.009896 | ECA4193 | putative amino-acid ABC transporter ATP-binding protein |
| ECA4194(ECAORF4154_1_sense) | 2.562858 | 0.036208 | 1.809572 | 0.046214 | ECA4194 | probable amino-acid ABC transporter permease protein |
| ECA4203(ECAORF4163_1_sense) | 1.73177 | 0.022114 |  |  | wzxE | enterobacterial common antigen (ECA) biosynthesis protein |
| ECA4205(ECAORF4165_1_sense) | 1.71376 | 0.003655 |  |  | rffC | lipopolysaccharide biosynthesis protein |
| ECA4208(ECAORF4168_1_sense) | 2.220126 | 0.026841 |  |  | wecB | UDP-N-acetylglucosamine 2-epimerase |
| ECA4211(ECAORF4171_1_sense) | 8.008307 | 0.03767 |  |  | rho | transcription termination factor Rho |
| ECA4213(ECAORF4173_1_sense) | 3.984401 | 3.44E-06 |  |  | rhlB | putative ATP-dependent RNA helicase |
| ECA4213(ECAORF4173_3_sense) | 4.730958 | 6.88E-04 |  |  | rhlB | putative ATP-dependent RNA helicase |
| ECA4215(ECAORF4175_1_sense) | 1.513836 | 0.006096 |  |  | rep | ATP-dependent DNA helicase |
| ECA4218(ECAORF4178_1_sense) | 1.667334 | 0.026361 |  |  | putP | sodium/proline symporter |
| ECA4221(ECAORF4181_1_sense) |  |  | 2.104958 | 0.018081 | ilvC | ketol-acid reductoisomerase |
| ECA4224(ECAORF4184_1_sense) | 0.492913 | 0.036665 |  |  | ECA4224 | conserved hypothetical protein |
| ECA4228(ECAORF4188_1_sense) | 1.964133 | 0.007007 |  |  | ilvM | acetolactate synthase isozyme II small subunit |
| ECA4233(ECAORF4193_1_sense) | 0.627553 | 0.041875 |  |  | ECA4233 | putative ABC transporter permease protein |
| ECA4237(ECAORF4197_1_sense) | 2.014302 | 0.037321 |  |  | murI | glutamate racemase |
| ECA4251(ECAORF4211_1_sense) |  |  | 1.591681 | 0.036369 | metL | bifunctional aspartokinase/homoserine dehydrogenase II |
| ECA4260(ECAORF4220_1_sense) | 3.790108 | 0.006738 |  |  | ftsN | cell division protein |
| ECA4264(ECAORF4224_1_sense) | 0.36987 | 0.038466 |  |  | menG | S-adenosylmethionine:2-demethylmenaquinone methyltransferase |
| ECA4267(ECAORF4227_1_sense) | 0.454672 | 0.010909 |  |  |  | PROBE08199 |
| ECA4274(ECAORF4234_1_sense) | 1.589212 | 0.018322 |  |  | ECA4274 | putative carbon-nitrogen hydrolase |
| ECA4277(ECAORF4237_1_sense) | 0.052617 | 9.42E-04 | 0.056424 | 3.74E-05 | ECA4277 | conserved hypothetical protein |
| ECA4277(ECAORF4237_3_sense) | 0.077868 | 0.002361 | 0.142976 | 0.020734 | ECA4277 | conserved hypothetical protein |
| ECA4278(ECAORF4238_1_sense) | 0.157133 | 0.005711 | 0.123799 | 0.004428 | ECA4278 | Rhs-family protein |
| ECA4279(ECAORF4239_1_sense) | 0.108542 | 0.008931 | 0.381895 | 0.024821 | ECA4279 | hypothetical protein |
| ECA4279(ECAORF4239_2_sense) | 0.095296 | 3.07E-05 | 0.29347 | 0.026849 | ECA4279 | hypothetical protein |
| ECA4279(ECAORF4239_3_sense) | 0.104968 | 0.003115 |  |  | ECA4279 | hypothetical protein |
| ECA4280(ECAORF4240_1_sense) | 1.75203 | 0.003924 |  |  | ECA4280 | conserved hypothetical protein |
| ECA4280(ECAORF4240_2_sense) | 1.810895 | 0.004526 |  |  | ECA4280 | conserved hypothetical protein |
| ECA4281(ECAORF4241_1_sense) | 1.929303 | 5.47E-04 |  |  | ECA4281 | putative phage regulatory protein |
| ECA4281(ECAORF4241_2_sense) | 1.86081 | 0.005194 |  |  | ECA4281 | putative phage regulatory protein |
| ECA4281(ECAORF4241_3_sense) | 2.404983 | 0.005769 |  |  | ECA4281 | putative phage regulatory protein |
| ECA4282(ECAORF4242_2_sense) | 2.085542 | 0.001187 |  |  | ECA4282 | DnaG primase-like protein |
| ECA4287(ECAORF4247_1_sense) | 0.096904 | 1.21E-04 | 0.248702 | 0.016256 | ECA4287 | hypothetical protein |
| ECA4287(ECAORF4247_2_sense) | 0.119044 | 2.45E-04 | 0.260545 | 0.010449 | ECA4287 | hypothetical protein |
| ECA4287(ECAORF4247_3_sense) | 0.129315 | 4.27E-04 | 0.243575 | 0.009197 | ECA4287 | hypothetical protein |
| ECA4288(ECAORF4248_1_sense) | 0.101222 | 0.004333 | 0.40661 | 0.00151 | ECA4288 | hypothetical protein |
| ECA4288(ECAORF4248_2_sense) | 0.097719 | 1.88E-04 | 0.295667 | 0.007487 | ECA4288 | hypothetical protein |
| ECA4288(ECAORF4248_3_sense) | 0.105231 | 0.004094 | 0.346344 | 0.034684 | ECA4288 | hypothetical protein |
| ECA4289(ECAORF4249_1_sense) | 1.661624 | 0.0128 |  |  | ECA4289 | conserved hypothetical protein |
| ECA4289(ECAORF4249_3_sense) | 1.614849 | 0.025184 |  |  | ECA4289 | conserved hypothetical protein |
| ECA4290(ECAORF4250_1_sense) | 2.092207 | 4.04E-04 |  |  | ECA4290 | putative phage regulatory protein |
| ECA4290(ECAORF4250_2_sense) | 1.829501 | 0.001479 |  |  | ECA4290 | putative phage regulatory protein |
| ECA4290(ECAORF4250_3_sense) | 2.433723 | 0.004501 |  |  | ECA4290 | putative phage regulatory protein |
| ECA4291(ECAORF4251_2_sense) | 2.723993 | 0.008476 |  |  | ECA4291 | DnaG primase-like protein |
| ECA4293(ECAORF4253_1_sense) | 0.262197 | 0.004323 |  |  | ECA4293 | hypothetical protein |
| ECA4294(ECAORF4254_1_sense) | 0.343296 | 0.003093 |  |  | ECA4294 | conserved hypothetical protein |
| ECA4297(ECAORF4257_3_sense) |  |  | 0.618422 | 0.03525 | astB | sulfate ester ABC transporter permease protein |
| ECA4305(ECAORF1528_1_sense) | 0.119504 | 0.047066 |  |  | sftR | LysR-family transcriptional regulator |
| ECA4305(ECAORF4265_1_sense) | 0.167106 | 0.01571 |  |  |  | PROBE08248 |
| ECA4307(ECAORF4267_1_sense) | 0.405554 | 0.022899 |  |  | pfkA | 6-phosphofructokinase isozyme I |
| ECA4308(ECAORF4268_1_sense) | 1.887648 | 0.021715 |  |  | cepA | cation efflux pump |
| ECA4318(ECAORF4278_1_sense) |  |  | 1.551369 | 0.038956 | ugpQ | glycerophosphoryl diester phosphodiesterase |
| ECA4324(ECAORF4284_1_sense) | 0.194307 | 0.015401 |  |  | ECA4324 | conserved hypothetical protein |
| ECA4331(ECAORF4291_1_sense) | 6.610162 | 0.022777 |  |  |  | PROBE08296 |
| ECA4333(ECAORF4293_1_sense) | 0.24998 | 4.40E-04 |  |  | ECA4333 | methyl-accepting chemotaxis protein |
| ECA4334(ECAORF4294_1_sense) |  |  | 0.493776 | 0.001645 | ECA4334 | methyl-accepting chemotaxis protein |
| ECA4341(ECAORF4301_1_sense) | 0.515661 | 0.003125 |  |  | livK | leucine-specific binding protein |
| ECA4345(ECAORF4305_1_sense) | 1.356383 | 0.029302 |  |  | ftsE | cell division ATP-binding protein |
| ECA4354(ECAORF4314_1_sense) |  |  | 0.563374 | 0.034363 | ECA4354 | putative membrane protein |
| ECA4356(ECAORF4316_1_sense) | 1.778573 | 0.037036 |  |  | ECA4356 | hypothetical protein |
| ECA4364(ECAORF4324_1_sense) |  |  | 1.813515 | 0.031902 | ECA4364 | putative membrane protein |
| ECA4365(ECAORF4325_1_sense) | 2.514054 | 0.02114 |  |  | ECA4365 | putative membrane protein |
| ECA4367(ECAORF4327_3_sense) | 0.571973 | 0.023171 |  |  | ECA4367 | hypothetical protein |
| ECA4368(ECAORF4328_1_sense) | 0.475976 | 9.52E-04 |  |  | ECA4368 | conserved hypothetical protein |
| ECA4385(ECAORF4343_1_sense) | 0.332533 | 0.027669 |  |  | ECA4385 | putative exported protein |
| ECA4391(ECAORF4350_1_sense) | 1.620686 | 1.14E-05 |  |  | dppD | dipeptide transport ATP-binding protein |
| ECA4392(ECAORF4351_1_sense) | 1.851444 | 0.011284 |  |  | dppC | dipeptide transport system permease protein |
| ECA4393(ECAORF4352_1_sense) | 1.823355 | 0.011427 |  |  | dppB | dipeptide transport system permease protein |
| ECA4393(ECAORF4352_2_sense) | 1.503817 | 0.014899 |  |  | dppB | dipeptide transport system permease protein |
| ECA4393(ECAORF4352_3_sense) | 2.719555 | 0.01542 |  |  | dppB | dipeptide transport system permease protein |
| ECA4393(ECAORF4353_1_sense) | 1.956816 | 0.034017 |  |  |  | PROBE09040 |
| ECA4393(ECAORF4353_2_sense) | 1.824063 | 0.007026 |  |  |  | PROBE09041 |
| ECA4393(ECAORF4353_3_sense) | 2.473964 | 8.81E-04 |  |  |  | PROBE09044 |
| ECA4394(ECAORF4354_1_sense) | 2.370676 | 0.014333 |  |  |  | PROBE08412 |
| ECA4399(ECAORF4359_1_sense) | 2.154977 | 0.034463 | 1.872962 | 8.93E-04 | dcuS | two-component sensor kinase |
| ECA4401(ECAORF4361_1_sense) | 0.381924 | 0.010428 |  |  | ECA4401 | putative membrane protein |
| ECA4405(ECAORF4365_1_sense) | 1.557277 | 0.009455 |  |  | ECA4405 | conserved hypothetical protein |
| ECA4406(ECAORF4366_1_sense) | 10.38533 | 0.036126 |  |  | ECA4406 | putative exported protein |
| ECA4413(ECAORF4374_1_sense) | 2.004505 | 0.043122 |  |  | ssuB | putative aliphatic sulfonates transport ATP-binding protein |
| ECA4414(ECAORF4375_1_sense) | 1.908308 | 0.004408 |  |  | ECA4414 | putative exported protein |
| ECA4414(ECAORF4375_2_sense) | 1.837229 | 0.001518 |  |  | ECA4414 | putative exported protein |
| ECA4428(ECAORF4392_1_sense) |  |  | 1.81704 | 9.46E-05 | ECA4428 | putative haloacid dehalogenase-like hydrolase |
| ECA4429(ECAORF4393_1_sense) | 0.637796 | 0.022652 |  |  | ECA4429 | putative transposase |
| ECA4429(ECAORF4393_3_sense) | 0.632989 | 0.014997 |  |  | ECA4429 | putative transposase |
| ECA4430(ECAORF4394_1_sense) | 0.512706 | 0.025337 |  |  | ECA4430 | putative lipoprotein |
| ECA4431(ECAORF4395_1_sense) | 0.427581 | 5.18E-04 | 0.533081 | 0.005798 | ECA4431 | hypothetical protein |
| ECA4432(ECAORF1424_1_sense) | 0.434974 | 0.048929 |  |  | bglA | 6-phospho-beta-glucosidase |
| ECA4432(ECAORF4396_2_sense) | 4.772351 | 0.048038 |  |  |  | PROBE09090 |
| ECA4432(ECAORF4396_3_sense) | 3.162648 | 0.031514 |  |  |  | PROBE09092 |
| ECA4432(ECAORF4397_2_sense) | 3.142715 | 0.048498 |  |  |  | PROBE09096 |
| ECA4432(ECAORF4397_3_sense) | 3.053829 | 0.039553 |  |  |  | PROBE09098 |
| ECA4432(ECAORF4398_3_sense) | 3.430224 | 0.045133 |  |  |  | PROBE09103 |
| ECA4438(ECAORF4405_1_sense) | 2.238198 | 3.50E-04 |  |  | gyrB | DNA gyrase subunit B |
| ECA4439(ECAORF4406_1_sense) | 3.34507 | 0.003231 |  |  | recF | DNA replication and repair protein |
| ECA4439(ECAORF4406_3_sense) | 3.488891 | 3.72E-04 |  |  | recF | DNA replication and repair protein |
| ECA4443(ECAORF4410_1_sense) | 2.935956 | 3.99E-05 |  |  | ECA4443 | 50S ribosomal protein L34 |
| ECA4443(ECAORF4410_2_sense) | 3.003286 | 0.002364 |  |  | ECA4443 | 50S ribosomal protein L34 |
| ECA4443(ECAORF4410_3_sense) | 2.825005 | 0.005387 |  |  | ECA4443 | 50S ribosomal protein L34 |
| ECA4444(ECAORF4411_1_sense) | 5.343547 | 0.005245 |  |  | rnpA | ribonuclease P protein component |
| ECA4445(ECAORF4412_1_sense) | 2.900783 | 4.39E-04 |  |  | ECA4445 | putative membrane protein |
| ECA4448(ECAORF4415_1_sense) |  |  | 0.361443 | 0.006936 | ECA4448 | conserved hypothetical protein |
| ECA4455(ECAORF4423_1_sense) | 4.028488 | 0.001547 |  |  | ECA4455 | conserved hypothetical protein |
| ECA4458(ECAORF4426_1_sense) | 3.104633 | 0.010592 | 3.022284 | 7.19E-04 | ECA4458 | putative lipoprotein |
| ECA4460(ECAORF4428_1_sense) | 4.225565 | 0.013411 | 4.154501 | 3.77E-05 | ECA4460 | putative lipoprotein |
| ECA4461(ECAORF4429_1_sense) | 0.543548 | 0.042219 |  |  | aegA | anaerobically expressed oxidoreductase |
| ECA4461(ECAORF4429_2_sense) | 0.465512 | 0.027501 |  |  | aegA | anaerobically expressed oxidoreductase |
| ECA4461(ECAORF4429_3_sense) | 0.15348 | 0.00878 | 0.429205 | 0.043905 | aegA | anaerobically expressed oxidoreductase |
| ECA4461(ECAORF4430_3_sense) | 0.222155 | 0.007757 |  |  |  | PROBE09152 |
| ECA4464(ECAORF4433_1_sense) | 2.874633 | 3.09E-04 | 2.15813 | 0.030994 | ECA4464 | putative permease |
| ECA4466(ECAORF4435_1_sense) |  |  | 1.831372 | 0.01104 | ECA4466 | putative haloacid dehalogenase-like hydrolase |
| ECA4468(ECAORF4437_1_sense) | 2.815555 | 0.014132 |  |  | ECA4468 | binding-protein-dependent transport system inner membrane component |
| ECA4470(ECAORF4438_1_sense) | 0.039532 | 0.023017 |  |  | ECA4470 | extracellular solute-binding protein |
| ECA4473(ECAORF4441_1_sense) |  |  | 2.07283 | 8.09E-04 | phoU | phosphate transport system protein |
| ECA4480(ECAORF4448_1_sense) | 1.672289 | 0.024119 |  |  | ECA4480 | probable amino acid ABC transporter, permease protein |
| ECA4483(ECAORF2849_1_sense) |  |  | 2.51725 | 0.023253 | nac | nitrogen assimilation regulatory protein |
| ECA4509(ECAORF4477_1_sense) | 0.537849 | 0.018224 |  |  | glmU | bifunctional GlmU protein |
| ECA4511(ECAORF4479_1_sense) | 2.159954 | 0.043281 | 2.078354 | 0.002257 | atpC | ATP synthase epsilon chain |
| ECA4512(ECAORF4480_1_sense) | 2.300986 | 0.012367 |  |  | atpD | ATP synthase beta chain |
| ECA4514(ECAORF4482_1_sense) | 2.748584 | 3.67E-04 |  |  | atpA | ATP synthase alpha chain |
| ECA4521(ECAORF4489_1_sense) | 3.384835 | 0.011798 |  |  | gidA | glucose inhibited division protein A |
| fiS(ECAORF0250_1_sense) | 2.273117 | 0.035879 |  |  | fiS | None |
| hcpA(ECAORF3392_1_sense) | 0.027421 | 6.71E-04 | 0.016759 | 0.00309 | hcpA | None |
| hcpA(ECAORF3392_2_sense) | 0.014407 | 0.005864 | 0.021535 | 0.005159 | hcpA | None |
| hcpA(ECAORF3392_3_sense) | 0.01251 | 7.71E-05 | 0.016579 | 1.45E-05 | hcpA | None |
| hcpA(ECAORF4235_1_sense) | 0.041465 | 0.001978 | 0.021019 | 0.002184 |  | PROBE08949 |
| hcpA(ECAORF4235_2_sense) | 0.014054 | 0.004298 | 0.018875 | 0.004088 |  | PROBE08952 |
| hcpA(ECAORF4235_3_sense) | 0.017548 | 0.009069 | 0.015151 | 2.22E-05 |  | PROBE08953 |
| hexA(ECAORF2991_1_sense) | 4.422116 | 0.004536 | 2.620934 | 0.001994 | hexA | None |
| hexA(ECAORF2991_2_sense) | 3.144286 | 7.18E-05 | 3.676805 | 0.032452 | hexA | None |
| hexA(ECAORF2991_3_sense) | 2.433012 | 0.002132 |  |  | hexA | None |
| hexA(ECAORF2993_1_sense) | 4.306316 | 2.64E-04 | 2.054157 | 0.005855 |  | PROBE08842 |
| hexA(ECAORF2993_2_sense) | 3.264485 | 9.85E-06 |  |  |  | PROBE08843 |
| hexA(ECAORF2993_3_sense) | 2.235016 | 0.034749 | 2.34828 | 0.001091 |  | PROBE08845 |
| hns(ECAORF1632_1_sense) | 0.515537 | 2.44E-05 |  |  | hns | None |
| hns(ECAORF2854_1_sense) |  |  | 1.64057 | 0.024918 |  | PROBE05572 |
| HpaC(ECAORF2124_1_sense) | 0.428665 | 0.042832 |  |  | HpaC | None |
| licB(ECAORF3606_1_sense) | 2.34278 | 0.018542 |  |  | licB | None |
| oppA(ECAORF2290_1_sense) | 2.119216 | 4.63E-05 |  |  |  | PROBE04459 |
| pel-3(ECAORF1067_1_sense) | 0.018443 | 2.14E-05 | 0.023157 | 0.002885 | pel-3 | None |
| uxaB(ECAORF4341_1_sense) |  |  | 1.492435 | 0.017929 |  | PROBE08391 |

**Supporting Table 2.** *P. atrosepticum* coding sequences in an *expI* mutant showing a statistically significant (P<0.05) change in transcript abundance (1.5 fold) compared to the wild type strain following microarray analysis of RNA from potato tubers at 12 and 20 hour post inoculation.
